# T-cell transcriptomics from peripheral blood highlights differences between polymyositis and dermatomyositis patients

**DOI:** 10.1186/s13075-018-1688-7

**Published:** 2018-08-29

**Authors:** Miranda Houtman, Louise Ekholm, Espen Hesselberg, Karine Chemin, Vivianne Malmström, Ann M. Reed, Ingrid E. Lundberg, Leonid Padyukov

**Affiliations:** 1 Division of Rheumatology, Department of Medicine, Karolinska Institutet, Karolinska University Hospital, Stockholm, Sweden; 2 Department of Pediatrics, Duke Children’s Hospital, Duke University Medical Center, Durham, USA

**Keywords:** Idiopathic inflammatory myopathies, Polymyositis, Dermatomyositis, T cells, CD4+ T cells, CD8+ T cells, Differential gene expression, RNA sequencing

## Abstract

**Background:**

Polymyositis (PM) and dermatomyositis (DM) are two distinct subgroups of idiopathic inflammatory myopathies, a chronic inflammatory disorder clinically characterized by muscle weakness and inflammatory cell infiltrates in muscle tissue. In PM, a major component of inflammatory cell infiltrates is CD8+ T cells, whereas in DM, CD4+ T cells, plasmacytoid dendritic cells, and B cells predominate. In this study, with the aim to differentiate involvement of CD4+ and CD8+ T-cell subpopulations in myositis subgroups, we investigated transcriptomic profiles of T cells from peripheral blood of patients with myositis.

**Methods:**

Total RNA was extracted from CD4+ T cells (PM = 8 and DM = 7) and CD8+ T cells (PM = 4 and DM = 5) that were isolated from peripheral blood mononuclear cells via positive selection using microbeads. Sequencing libraries were generated using the Illumina TruSeq Stranded Total RNA Kit and sequenced on an Illumina HiSeq 2500 platform, yielding about 50 million paired-end reads per sample. Differential gene expression analyses were conducted using DESeq2.

**Results:**

In CD4+ T cells, only two genes, *ANKRD55* and *S100B*, were expressed significantly higher in patients with PM than in patients with DM (false discovery rate [FDR] < 0.05, model adjusted for age, sex, HLA-DRB1*03 status, and RNA integrity number [RIN]). On the contrary, in CD8+ T cells, 176 genes were differentially expressed in patients with PM compared with patients with DM. Of these, 44 genes were expressed significantly higher in CD8+ T cells from patients with PM, and 132 genes were expressed significantly higher in CD8+ T cells from patients with DM (FDR < 0.05, model adjusted for age, sex, and RIN). Gene Ontology analysis showed that genes differentially expressed in CD8+ T cells are involved in lymphocyte migration and regulation of T-cell differentiation.

**Conclusions:**

Our data strongly suggest that CD8+ T cells represent a major divergence between PM and DM patients compared with CD4+ T cells. These alterations in the gene expression in T cells from PM and DM patients might advocate for distinct immune mechanisms in these subphenotypes of myositis.

**Electronic supplementary material:**

The online version of this article (10.1186/s13075-018-1688-7) contains supplementary material, which is available to authorized users.

## Background

Polymyositis (PM) and dermatomyositis (DM) are chronic inflammatory disorders clinically characterized by skeletal muscle weakness and muscle inflammation [1]. Other organs, such as the skin, joints, and lungs, are frequently involved in these disorders. Although the etiology of PM and DM is unknown, certain environmental and genetic factors are important. The major risk factor for these disorders in Caucasian populations is HLA-DRB1*03:01 [2–4]. In addition, autoantibodies are found in more than 80% of the PM and DM patients, supporting a role for the adaptive immune system in the pathogenesis of these disorders [5].

In both PM and DM patients, inflammatory cell infiltrates are commonly found in the affected tissues [6, 7]. In PM, the cellular infiltrates are located mainly in the endomysium surrounding muscle fibers and typically dominated by CD8+ T cells [8, 9]. In contrast, in patients with DM, the inflammatory cell infiltrates are located mainly in the perimysium and in perivascular areas, and the infiltrates are predominated by CD4+ T cells with occasional plasmacytoid dendritic cells and B cells [6]. Further phenotyping of T cells in muscle tissue has led to the observation that the muscle-infiltrating T cells in both PM and DM are predominantly of the CD8+CD28^null^ and CD4+CD28^null^ phenotypes, which both have cytotoxic properties [10, 11]. Interestingly, these subpopulations of T cells can also be detected in peripheral blood of patients with myositis [10, 12]. Still, the differences in the tissue location of inflammatory cell infiltrates suggest that the underlying immune mechanisms may vary between PM and DM.

In this study, we aimed to investigate whole-genome transcriptomes of CD4+ and CD8+ T cells from peripheral blood in different subsets of patients with idiopathic inflammatory myopathies (IIMs). We used RNA sequencing to identify differentially expressed genes between PM and DM, as well as in patients with both types of IIM, considering HLA-DRB1*03 alleles.

## Methods

### Patient recruitment

Initially, 33 consecutive adult individuals with PM or DM (not drug-free) from the Karolinska Hospital Rheumatology Clinic were selected for the study on the basis of diagnosis (PM and DM) and HLA-DRB1*03 status (positive and negative). Patients with myositis visited the clinic between January 21 and April 23, 2014, and were fully validated according to the new European League Against Rheumatism/American College of Rheumatology classification criteria [13]. Thirty-one of the 33 patients also satisfied the Bohan and Peter criteria [14, 15]. Extensive clinical data, including disease phenotypes and treatment regimen, were collected from clinical records by experienced rheumatologists. All patients gave written consent for their participation in the study. The study was approved by the Stockholm regional ethics board.

### Autoantibody detection

Patient sera were analyzed by RNA and protein immunoprecipitation for the presence of autoantibodies against Jo1, PL12, PL7, OJ, EJ, KS, Mi-2, MDA5, TIF-1γ, SRP, PM-Scl, Ro52, Ro60, U1RNP, and Ku. Sera collected after 2013 were screened using a validated line immunoassay system (EUROLINE myositis panel 4; EUROIMMUN AG, Lübeck, Germany) according to the manufacturer’s instructions or by enzyme-linked immunosorbent assays for the presence of myositis-specific autoantibodies or myositis-associated autoantibodies.

### *HLA* typing

*HLA* typing was performed by sequence-specific primer PCR (HLA-DR low-resolution kit; Olerup SSP, Stockholm, Sweden) and analyzed by agarose gel electrophoresis [16]. An interpretation table was used to determine the specific genotype according to the manufacturer’s instructions.

### Blood sampling and cell sorting

Patients’ blood was collected in heparin tubes (40–50 ml in total), and peripheral blood mononuclear cells (PBMCs) were isolated by density gradient centrifugation using Ficoll density gradient medium (GE Healthcare Bio-Sciences AB, Uppsala, Sweden). CD4+ cells and CD8+ cells were isolated from the PBMCs via positive selection using CD4 or CD8 MicroBeads on an autoMACS® Pro Separator (Miltenyi Biotec Norden AB, Lund, Sweden). Flow cytometry was used to determine the purity of some of the sorted T-cell samples, and over 90% of CD45+ cells expressed CD4 (*n* = 5) or CD8 (*n* = 5). The following antibodies were used: CD45 (HI30; BioLegend, San Diego, CA, USA), CD4 (OKT4; BioLegend), and CD8 (SK1; BD Biosciences, Stockholm, Sweden).

### RNA sequencing

Total RNA was extracted with the RNeasy Mini Kit (Qiagen AB, Sollentuna, Sweden) according to the manufacturer’s instructions. Samples were treated with DNase (Qiagen) for 20 minutes at room temperature to avoid contamination with genomic DNA. The quality of each RNA sample was characterized using a RNA 6000 Nano Chip (Agilent Technologies Sweden AB, Kista, Sweden) on the Agilent Bioanalyzer 2100. Fifteen CD4+ T-cell samples and nine CD8+ T-cell samples met the RNA quality criteria (RNA integrity number [RIN] > 4) and were sequenced. The RNA was fragmented and prepared into sequencing libraries using the TruSeq Stranded Total RNA Sample Preparation Kit (Illumina, San Diego, CA, USA) with ribosomal depletion using Ribo-Zero (2 × 125 bp; Illumina) and analyzed on an Illumina HiSeq 2500 sequencer (SNP&SEQ Technology Platform, Uppsala, Sweden). On average, 50 million reads were produced per sample. Raw read quality was evaluated using FastQC. Prefiltering on quality of reads using cutadapt (version 1.9.1) was applied (−q 30 -a AGATCGGAAGAGCACACGTCTGAACTCCAGTCAC -A AGATCGGAAGAGCGTCGTGTAGGGAAAGAGTGTAGATCTCGGTGGTCGCCGTATCATT -m 40). Filtered reads were aligned to the hg38 assembly and quantified using STAR (version 2.5.1b) [17] with default settings.

### Cell-type enrichment analysis

The xCell tool [18] was used to identify cellular heterogeneity in the CD4+ and CD8+ T-cell subsets from gene expression data. xCell uses the expression levels ranking (transcripts per million), and these were obtained using Salmon (version 0.8.2) [19].

### Differential gene expression analysis

Raw expression counts were adjusted for library size using the R package DESeq2 (version 1.16.1) [20]. Prefiltering of low-count genes was performed to keep only genes that have at least 50 reads in total. Principal component analysis (PCA) was used to identify outliers. For each sample, the first five principal components (PCs) were extracted and correlated with available clinical and technical data. For the CD4+ T-cell subset, age group (< 60 and ≥ 60 years), sex, HLA-DRB1*03 status (positive and negative), and RIN value were used as covariates in the analyses. The formula used for defining the model was the following:

$$ \mathrm{Geneexpression}\sim \mathrm{agegroup}+\mathrm{sex}+ HLA- DRB 1\ast 03\mathrm{status}+\mathrm{RINvalue}+\mathrm{diagnosis} $$

For the CD8+ T-cell subset, sex, age group (< 60 and ≥ 60 years), and RIN value were used as covariates in the analyses. The formula used for defining the model was the following:

$$ \mathrm{Geneexpression}\sim \mathrm{agegroup}+\mathrm{sex}+\mathrm{RINvalue}+\mathrm{diagnosis} $$

Owing to possible contamination of samples with other cell types, differential gene expression analyses were performed in two stages. The analyses were performed first on all samples and second on a subset of samples excluding potential outliers. The differentially expressed genes that overlapped between the two analyses were taken as robust evidence for significant findings in order to exclude false-positive findings due to heterogeneity in the CD4+ and CD8+ T-cell subsets.

The default DESeq2 options were used, including log fold change shrinkage. We considered differentially expressed genes when the Benjamini-Hochberg adjusted *p* value (false discovery rate [FDR]) was < 0.05.

### Functional enrichment analysis

To further understand the biological relevance and associated pathways of the differentially expressed genes, functional enrichment analysis was performed using the Gene Ontology (GO) database (released on 2 February 2018). Fisher’s exact test with FDR correction (< 0.05) was used to determine significantly enriched GO biological processes.

## Results

### Differential gene expression in CD4+ T cells of PM and DM patients

The clinical characteristics of patients with myositis in the CD4+ T-cell subset are summarized in Table 1. No significant differences were found in patients with PM compared with patients with DM regarding disease activity measures and laboratory data (Additional file 1: Table S1 and S2).

**Table 1:** Clinical characteristics of patients with myositis at time of blood sampling

Patient	CD4	CD8	Age (years)	Sex	Diagnosis	Autoantibodies	HLA-DRB1*03 status (genotype)	Prednisone	Other treatment
CEL-004	×		76	Female	PM, prob	PM-Scl	Positive (*03/*07)	Yes	MTX
CEL-005	×		48	Male	DM, def	SSA	Negative (*11/*11)	Yes	AZA, TAC
CEL-006	×		60	Male	PM, pos	SRP	Positive (*03/*15)	Yes	–
CEL-008	×	×	47	Male	DM, prob	MDA5	Negative (*11/*16)	Yes	AZA, ABT
CEL-009		×	80	Male	PM, def	Jo1	Positive (*03/*13)	Yes	–
CEL-010	×		74	Female	PM, prob	None	Positive (*03/*10)	No	IVIg
CEL-011	×	×	55	Female	DM, def	Mi-2	Negative (*07/*16)	Yes	MTX
CEL-014		×	74	Male	DM, def	SSA	Negative (*07/*11)	No	–
CEL-016	×		78	Male	DM, def	None	Positive (*03/*11)	Yes	MMF
CEL-017	×	×	80	Female	PM, def	None	Positive (*03/*13)	Yes	MTX
CEL-019	×		46	Female	DM, def	PM-Scl	Positive (*03/*04)	No	MTX
CEL-020	×		61	Female	PM, def	PM-Scl	Positive (*01/*03)	Yes	MMF
CEL-023	×		58	Male	DM, def	TIF1γ	Negative (*04/*07)	Yes	AZA, RIX
CEL-024	×	×	63	Female	PM, def	SSA	Positive (*03/*08)	Yes	AZA, CsA
CEL-027		×	65	Male	PM, def	Jo1	Positive (*03/*08)	Yes	–
CEL-030	×		40	Female	PM, def	Jo1	Positive (*03/*03)	Yes	–
CEL-031	×	×	68	Female	DM, def	TIF1γ	Negative (*01/*11)	Yes	–
CEL-033		×	42	Male	DM, def	MDA5	Negative (*04/*07)	Yes	MTX
CEL-034	×		56	Female	PM, def	Jo1, SSA, SSB	Negative (*11/*16)	Yes	MTX

*Abbreviations: ABT* Abatacept, *AZA* Azathioprine, *CsA* Cyclosporine A, *Def* Definite, *DM* Dermatomyositis, *IVIg* Intravenous immunoglobulin, *MMF* Mycophenolate mofetil, *MTX* Methotrexate, *PM* Polymyositis, *Pos* Possible, *Prob* Probable, *RIX* Rituximab, *SRP* Signal recognition particle, *TAC* Tacrolimus

Demographic and clinical characteristics of patients with myositis in the CD4+ (*n* = 15) and CD8+ T-cell subsets (*n* = 9) at time of blood sampling. The patients were classified according to the new European League Against Rheumatism/American College of Rheumatology classification criteria [13]

**Fig. 1:**
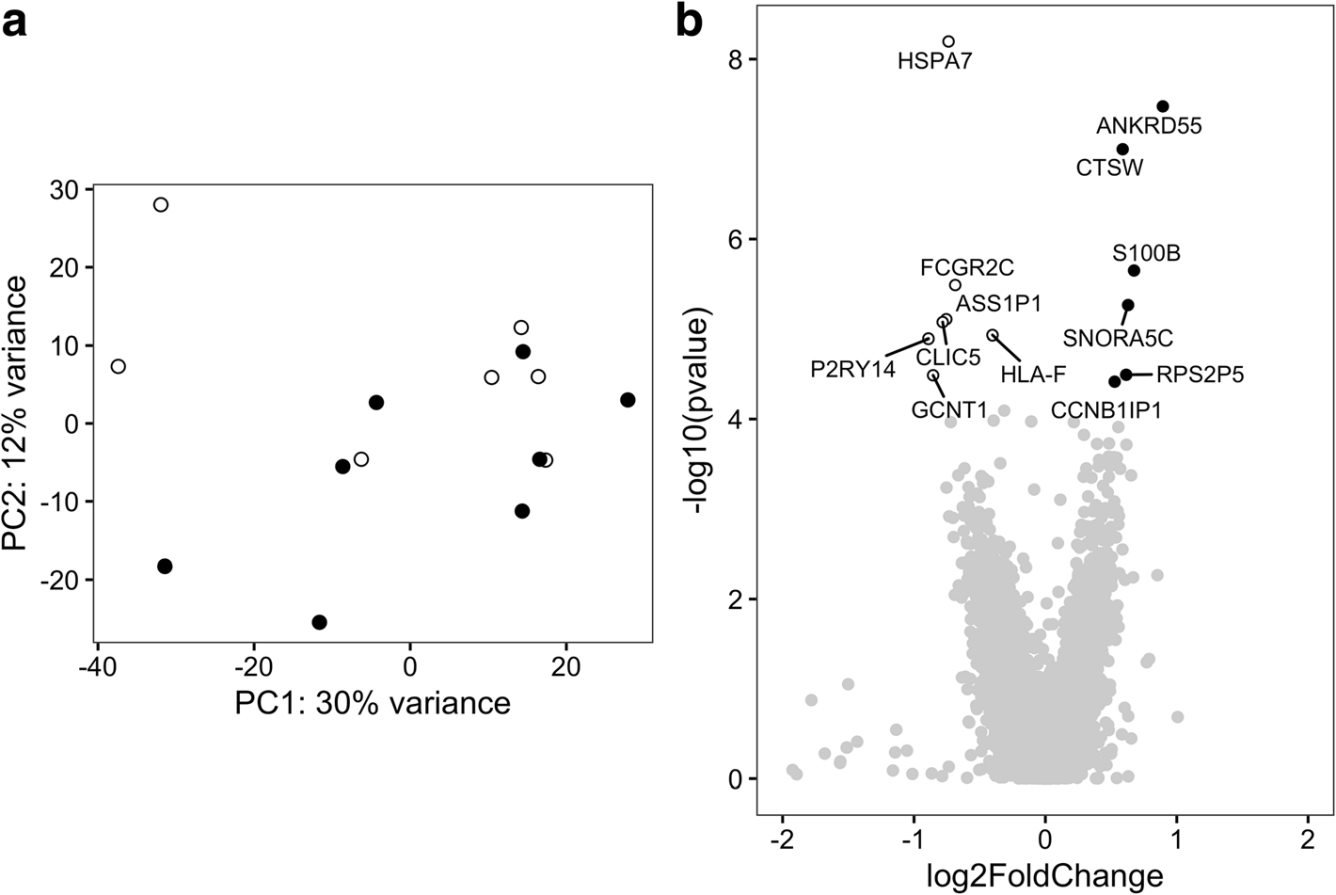
Gene expression profile in CD4+ T cells of polymyositis (PM) and dermatomyositis (DM) patients. **a** Principal components (PCs) 1 and 2 plotted according to the diagnosis of the patients in a dataset of 21,008 genes (*n* = 15). Samples from patients with PM are represented by *filled circles*, and those from DM patients are represented by *open circles*. **b** Differential genome-wide transcriptomic profile for the contrast between PM and DM in CD4+ T cells. The fold changes (log_2_) are shown on the *x*-axis, and the *p* values (−log_10_) are shown on the *y*-axis. The genes that are expressed significantly higher in PM are shown as *filled circles*, and the genes expressed significantly higher in DM are shown as *open circles*. A false discovery rate threshold of 5% based on the method of Benjamini-Hochberg was used to identify significant differentially expressed genes

Because PM and DM represent two similar but clinically distinct diseases, we searched for differentially expressed genes in CD4+ T cells in PM versus DM patients. First, the whole-genome expression pattern in CD4+ T cells of PM and DM patients was examined by PCA. The first two PCs did not significantly separate between PM and DM (Fig. 1a), suggesting that, in general, the overall gene expression in CD4+ T cells from patients with PM or DM is similar. At the first analytical stage, we performed differential gene expression analysis using DESeq2 with sex, age group, HLA-DRB1*03 status, and RIN value as covariates. Based on the cutoff criteria of Benjamini-Hochberg based FDR correction of < 0.05, 13 genes were found to be differentially expressed. Among these genes, six were expressed higher in PM patients and seven were expressed higher in DM patients (Fig. 1b and Additional file 2: Table S6).

**Fig. 2:**
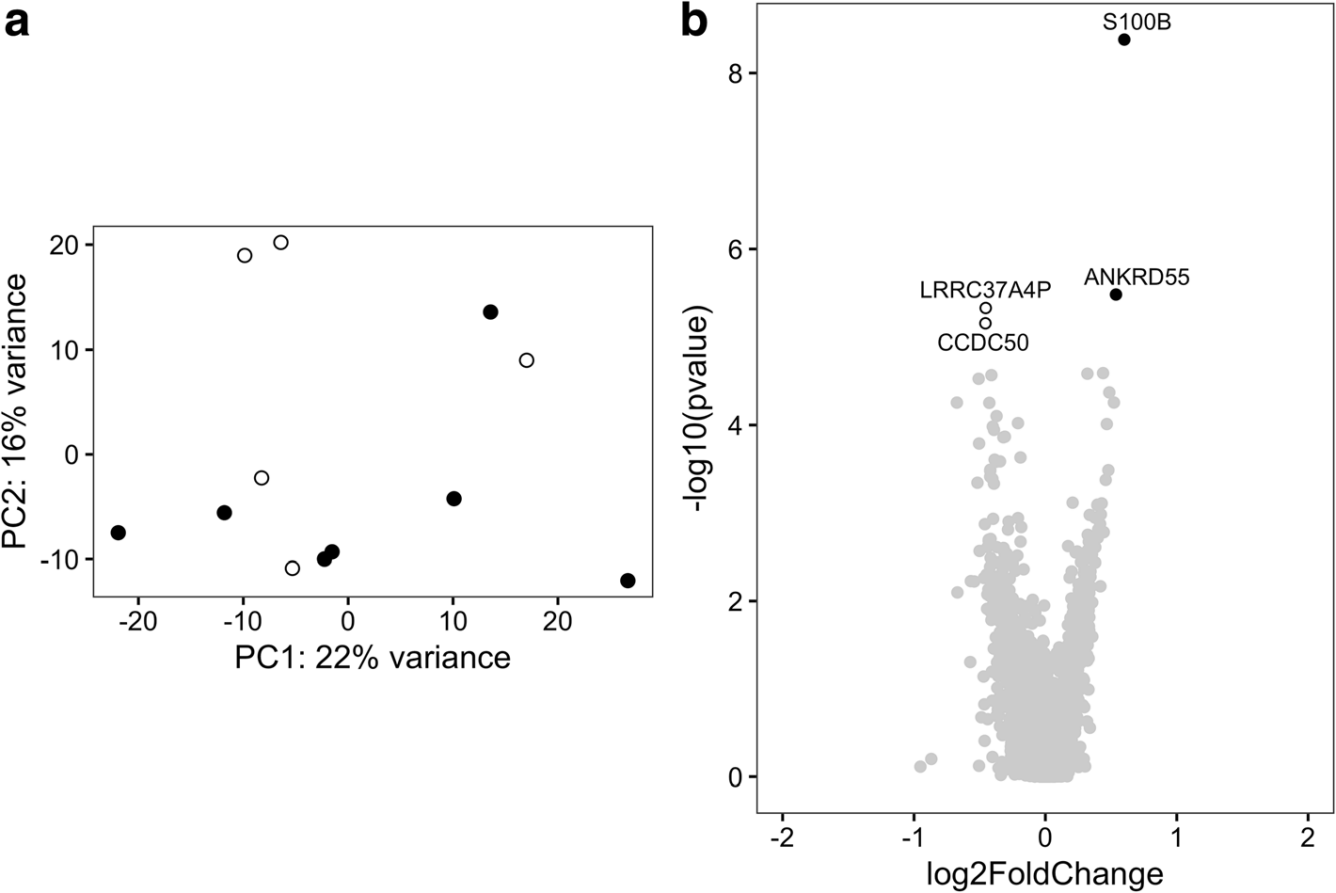
Gene expression profile in CD4+ T cells of polymyositis (PM) and dermatomyositis (DM) patients excluding potential outliers. **a** Principal components (PCs) 1 and 2 plotted according to the diagnosis of the patients in a dataset of 20,091 genes (*n* = 12). Samples from patients with PM are represented by *filled circles*, and those from patients with DM are represented by *open circles*. **b** Differential genome-wide transcriptomic profile for the contrast between PM and DM in CD4+ T cells. The fold changes (log_2_) are shown on the *x*-axis, and the *p* values (−log_10_) are shown on the *y*-axis. The genes that are expressed significantly higher in PM are shown by *filled circles*, and the genes expressed significantly higher in DM are shown by *open circles*. A false discovery rate threshold of 5% based on the method of Benjamini-Hochberg was used to identify significant differentially expressed genes

The PCA plot shown in Fig. 1a indicates three potential outliers with a PC1 score lower than − 20. These three samples represent higher gene expression levels related to monocytes according to the xCell tool (Additional file 3: Figure S1). To exclude the possibility that the differentially expressed genes were obtained because of a difference in cell composition, at the second analytical stage, we removed the three potential outliers from the analysis. This did not affect clustering of PM and DM samples in PCA (Fig. 2a). Using the same covariates as above, four genes were found to be differentially expressed in CD4+ T cells comparing PM patients with DM patients by applying an FDR correction cutoff of < 0.05. Two genes had a higher expression in CD4+ T cells of patients with PM compared with patients with DM, and two genes had a higher expression in CD4+ T cells of patients with DM compared with patients with PM (Fig. 2b and Additional file 2: Table S7). Thus, after accounting for possible contamination of the CD4+ T-cell population with monocytes, we considered only the genes that were found to be differentially expressed at both analytical stages. These analyses indicate that in CD4+ T cells, *ANKRD55* and *S100B* had a higher expression in CD4+ T cells of patients with PM compared with patients with DM.

### Differential gene expression in CD8+ T cells of PM and DM patients

The clinical characteristics of patients with myositis in the CD8+ T-cell subset are summarized in Table 1. No significant differences were found in patients with PM compared with patients with DM regarding disease activity measures and laboratory data (Additional file 1: Table S1 and S3).

**Fig. 3:**
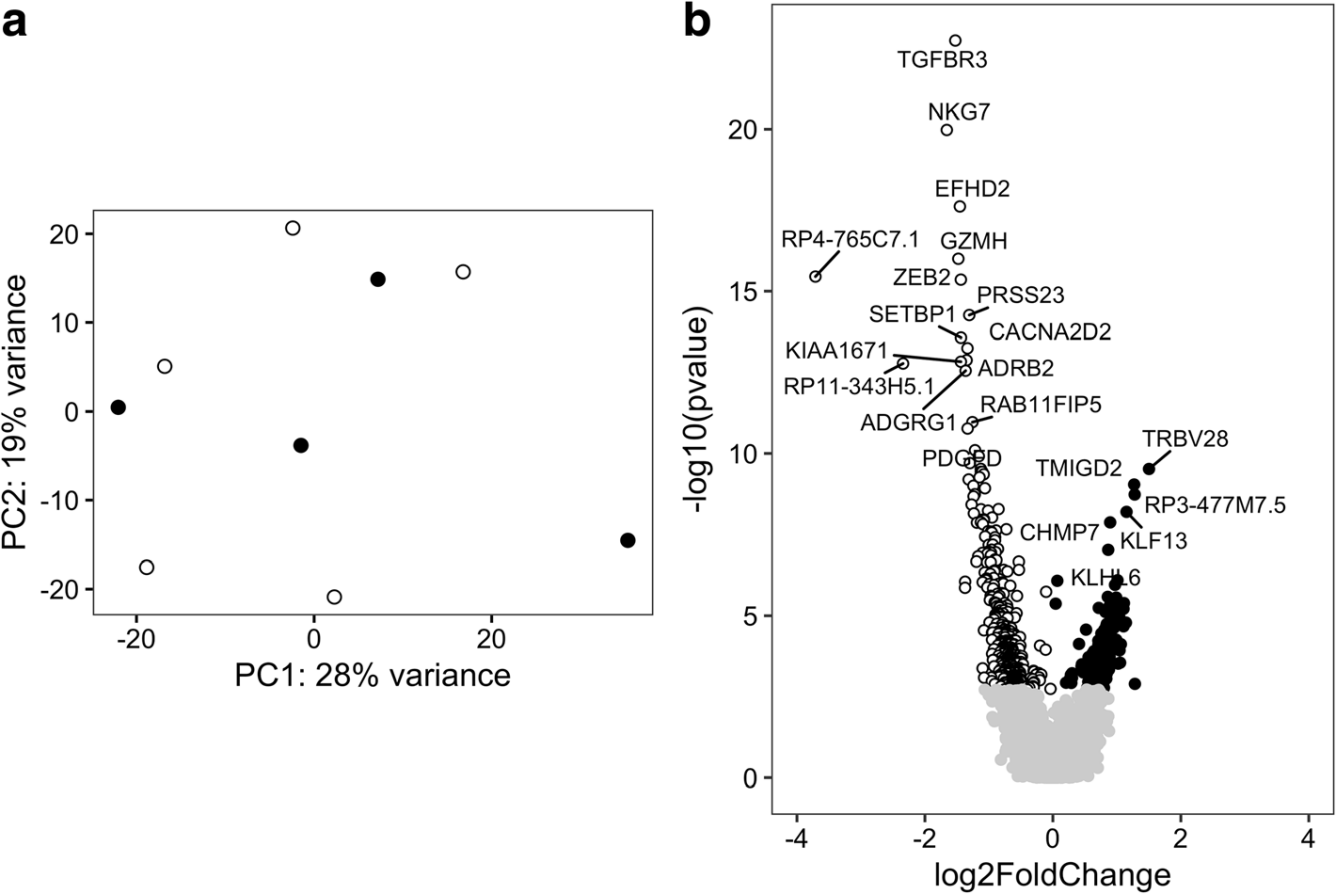
Gene expression profile in CD8+ T cells of polymyositis (PM) and dermatomyositis (DM) patients. **a** Principal components (PCs) 1 and 2 plotted according to the diagnosis of the patients in a dataset of 18,696 genes (*n* = 9). Samples from patients with PM are represented by *filled circles*, and those from patients with DM are represented by *open circles*. **b** Differential genome-wide transcriptomic profile for the contrast between PM and DM in CD8+ T cells. The fold changes (log_2_) are shown on the *x*-axis, and the *p* values (−log_10_) are shown on the *y*-axis. The genes that are expressed significantly higher in PM are shown by *filled circles*, and the genes expressed significantly higher in DM are shown by *open circles*. A false discovery rate threshold of 5% based on the method of Benjamini-Hochberg was used to identify significant differentially expressed genes. The symbols of the differentially expressed genes with an adjusted *p* value < 5 × 10^5^ and < 5 × 10^8^ are shown for PM and DM, respectively

Another cell type that is commonly found in affected tissues of patients with myositis are CD8+ T cells. Therefore, we also searched for differentially expressed genes between PM and DM in CD8+ T cells. First, the gene expression pattern in PM and DM was examined by PCA. PCA showed no clustering of PM and DM (Fig. 3a), which suggests that the overall gene expression in PM and DM is similar in CD8+ T cells. To identify genes that are differentially expressed between PM and DM, DESeq2 was used with sex, age group, and RIN value as covariates at the first analytical stage. Upon applying Benjamini-Hochberg-based FDR correction of < 0.05, we found that 588 genes were differentially expressed between PM and DM. Among these genes, 182 had a higher expression in CD8+ T cells of patients with PM patients compared with DM patients, and 406 had a higher expression in CD8+ T cells of patients with DM compared with patients with PM (Fig. 3b and Additional file 4: Table S8).

**Fig. 4:**
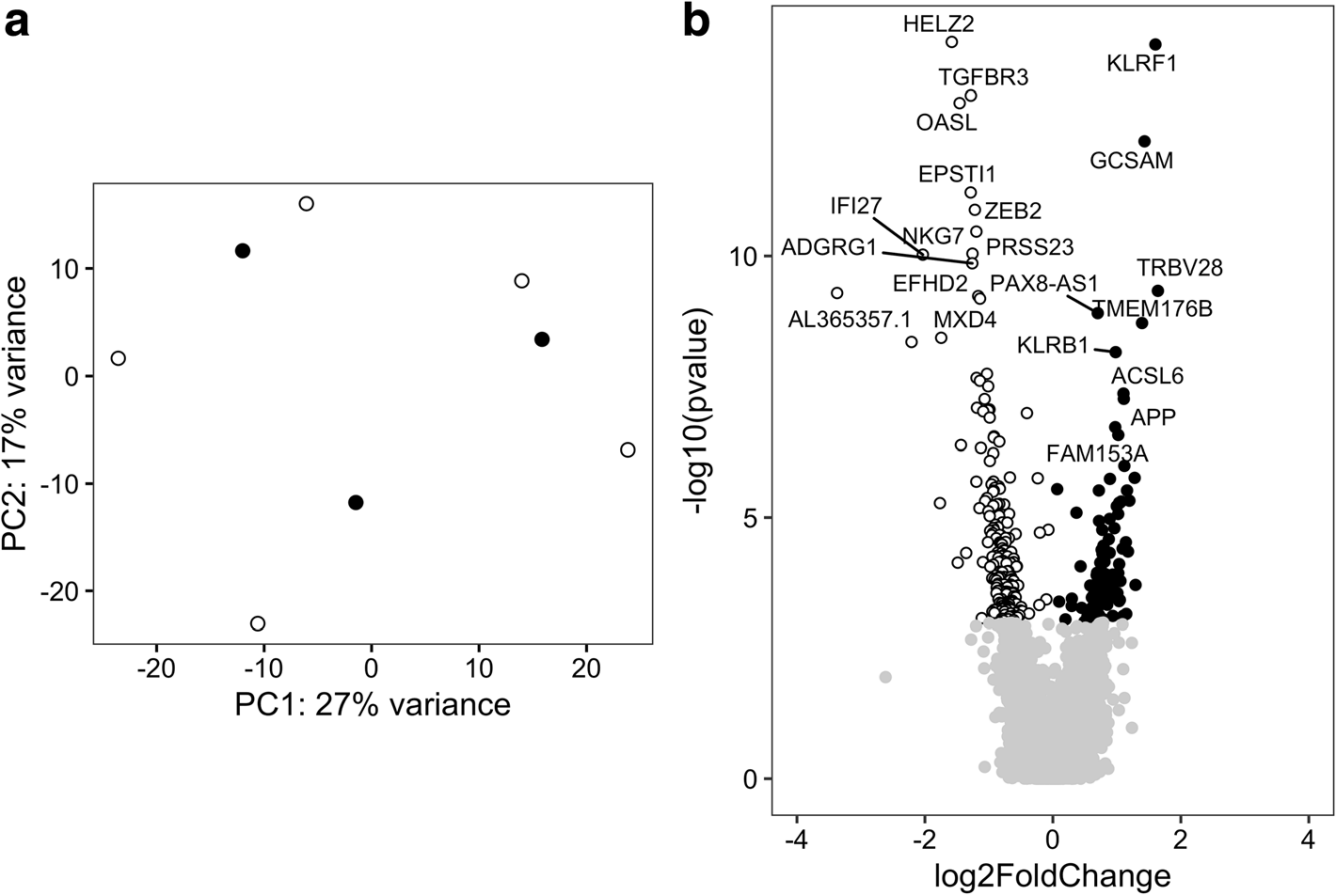
Gene expression profile in CD8+ T cells of polymyositis (PM) and dermatomyositis (DM) patients excluding potential outliers. **a** Principal components (PCs) 1 and 2 plotted according to the diagnosis of the patients in a dataset of 18,289 genes (*n* = 8). Samples from patients with PM are represented by *filled circles*, and those from patients with DM are represented by *open circles*. **b** Differential genome-wide transcriptomic profile for the contrast between PM and DM in CD8+ T cells. The fold changes (log_2_) are shown on the *x*-axis, and the *p* values (−log_10_) are shown on the *y*-axis. The genes that are expressed significantly higher in PM are shown by *filled circles*, and the genes expressed significantly higher in DM are shown by *open circles*. A false discovery rate threshold of 5% based on the method of Benjamini-Hochberg was used to identify significant differentially expressed genes. The symbols of the differentially expressed genes with an adjusted *p* value < 1 × 10^4^ and < 1 × 10^6^ are shown for PM and DM, respectively

The PCA plot shown in Fig. 3a indicates one potential outlier with a PC1 score > 20. This sample clustered together with the CD4+ T-cell samples (data not shown) and was removed from the analysis at the second analytical stage. After removing this sample from the analysis, the PCA showed that the overall gene expression remained similar in PM and DM (Fig. 4a). Based on the cutoff criteria of FDR < 0.05, 308 genes were found to be differentially expressed in CD8+ T cells comparing PM patients with DM patients. Among these genes, 107 genes had a higher expression in CD8+ T cells of PM patients compared with patients with DM, and 201 genes had a higher expression in CD8+ T cells of DM patients compared with PM patients (Fig. 4b and Additional file 4: Table S9).

Thus, after accounting for heterogeneity in the CD8+ T-cell subset, we considered only the genes that were found to be differentially expressed at both analytical stages. Together, a total of 44 genes were commonly expressed higher in CD8+ T cells of patients with PM compared with patients with DM (Table 2), and 132 genes were commonly expressed higher in CD8+ T cells of patients with DM compared with patients with PM (Table 3).

**Table 2:** Genes expressed significantly higher in CD8+ T cells of patients with polymyositis than in those with dermatomyositis

Gene symbol	Gene name
*TRBV28*	T cell receptor beta variable 28
*RP3-477M7.5*	
*TMIGD2*	Transmembrane and immunoglobulin domain containing 2
*KLF13*	Kruppel like factor 13
*CA6*	Carbonic anhydrase 6
*TMTC1*	Transmembrane and tetratricopeptide repeat containing 1
*LINC00402*	Long intergenic non-protein coding RNA 402
*TRBV30*	T cell receptor beta variable 30 (gene/pseudogene)
*IL6R*	Interleukin 6 receptor
*EPHA1*	EPH receptor A1
*XKR9*	XK related 9
*GABPB1-AS1*	GABPB1 antisense RNA 1
*LAPTM4B*	Lysosomal protein transmembrane 4 beta
*EPHA1-AS1*	EPHA1 antisense RNA 1
*CAMSAP2*	Calmodulin regulated spectrin associated protein family member 2
*AC012636.1*	Uncharacterized LOC101929215
*RP11-28F1.2*	
*CHMP7*	Charged multivesicular body protein 7
*SYNJ2*	Synaptojanin 2
*KLHL6*	Kelch like family member 6
*PRKCQ-AS1*	PRKCQ antisense RNA 1
*CASP10*	Caspase 10
*TXK*	TXK tyrosine kinase
*CD27*	CD27 molecule
*TBC1D4*	TBC1 domain family member 4
*CLN5*	CLN5, intracellular trafficking protein
*JAML*	Junction adhesion molecule like
*FAM153A*	Family with sequence similarity 153 member A
*TNFRSF10D*	TNF receptor superfamily member 10d
*DHX32*	DEAH-box helicase 32 (putative)
*STRBP*	Spermatid perinuclear RNA binding protein
*AL034550.2*	Uncharacterized LOC101929698
*DGKA*	Diacylglycerol kinase alpha
*COX10-AS1*	COX10 antisense RNA 1
*GCSAM*	Germinal center associated signaling and motility
*SLC7A6*	Solute carrier family 7 member 6
*ACSL6*	Acyl-CoA synthetase long chain family member 6
*AKAP7*	A-kinase anchoring protein 7
*AP005131.6*	
*UXS1*	UDP-glucuronate decarboxylase 1
*PAX8-AS1*	PAX8 antisense RNA 1
*C21orf33*	Chromosome 21 open reading frame 33
*RP11-65I12.1*	
*GSTM1*	Glutathione *S*-transferase mu 1

The table demonstrates the genes that overlap between the two analytical stages and have a significantly higher expression in CD8+ T cells of patients with polymyositis than in those with dermatomyositis. A false discovery rate threshold of 5% based on the method of Benjamini-Hochberg was used to identify significant differentially expressed genes

**Table 3:** Genes expressed significantly higher in CD8+ T cells of patients with dermatomyositis than in those with polymyositis

Gene symbol	Gene name
*AL365357.1*	Ribosomal protein S14 pseudogene 2
*AL591846.1*	Ribosomal protein S14 pseudogene 1
*NKG7*	Natural killer cell granule protein 7
*TGFBR3*	Transforming growth factor beta receptor 3
*GZMH*	Granzyme H
*EFHD2*	EF-hand domain family member D2
*ZEB2*	Zinc finger E-box binding homeobox 2
*KIAA1671*	KIAA1671
*SETBP1*	SET binding protein 1
*FAM118A*	Family with sequence similarity 118 member A
*ADGRG1*	Adhesion G protein-coupled receptor G1
*ADRB2*	Adrenoceptor beta 2
*CACNA2D2*	Calcium voltage-gated channel auxiliary subunit alpha2delta 2
*PDGFD*	Platelet-derived growth factor D
*SH3TC1*	SH3 domain and tetratricopeptide repeats 1
*PRSS23*	Serine protease 23
*TBKBP1*	TBK1 binding protein 1
*AC009951.1*	
*RAB11FIP5*	RAB11 family interacting protein 5
*GNAO1*	G protein subunit alpha o1
*MUC16*	Mucin 16, cell surface associated
*RP11-107E5.2*	
*KIF19*	Kinesin family member 19
*CST7*	Cystatin F
*SMAD7*	SMAD family member 7
*LINC02086*	Long intergenic non-protein coding RNA 2086
*AC040970.1*	Uncharacterized LOC101927963
*LLGL2*	LLGL2, scribble cell polarity complex component
*SYNE1*	Spectrin repeat containing nuclear envelope protein 1
*RAP1GAP2*	RAP1 GTPase activating protein 2
*FAM53B*	Family with sequence similarity 53 member B
*TOGARAM2*	TOG array regulator of axonemal microtubules 2
*FRMPD3*	FERM and PDZ domain containing 3
*TBX21*	T-box 21
*SESN2*	Sestrin 2
*PAX5*	Paired box 5
*MIDN*	Midnolin
*CCL5*	C-C motif chemokine ligand 5
*SYTL3*	Synaptotagmin like 3
*GAB3*	GRB2 associated binding protein 3
*TTC38*	Tetratricopeptide repeat domain 38
*LDLR*	Low density lipoprotein receptor
*CCL4*	C-C motif chemokine ligand 4
*DMWD*	DM1 locus, WD repeat containing
*CASZ1*	Castor zinc finger 1
*LAG3*	Lymphocyte activating 3
*DYRK1B*	Dual specificity tyrosine phosphorylation regulated kinase 1B
*GPR153*	G protein-coupled receptor 153
*MATK*	Megakaryocyte-associated tyrosine kinase
*SH2D2A*	SH2 domain containing 2A
*RHBDF2*	Rhomboid 5 homolog 2
*ADGRG5*	Adhesion G protein-coupled receptor G5
*UBE2Q2P1*	Ubiquitin conjugating enzyme E2 Q2 pseudogene 1
*GALNT3*	Polypeptide N-acetylgalactosaminyltransferase 3
*RUNX3*	Runt related transcription factor 3
*PLA2G16*	Phospholipase A2 group XVI
*SLC15A4*	Solute carrier family 15 member 4
*PPP2R2B*	Protein phosphatase 2 regulatory subunit beta
*RGS9*	Regulator of G protein signaling 9
*PATL2*	PAT1 homolog 2
*C1orf21*	Chromosome 1 open reading frame 21
*S1PR5*	Sphingosine-1-phosphate receptor 5
*TMCC3*	Transmembrane and coiled-coil domain family 3
*TLR3*	Toll like receptor 3
*GLB1L2*	Galactosidase beta 1 like 2
*PRELID2*	PRELI domain containing 2
*ADAP1*	ArfGAP with dual PH domains 1
*TRGJ2*	T cell receptor gamma joining 2
*DENND3*	DENN domain containing 3
*SOX13*	SRY-box 13
*GZMB*	Granzyme B
*FGFBP2*	Fibroblast growth factor binding protein 2
*RAP2A*	RAP2A, member of RAS oncogene family
*FCRL6*	Fc receptor like 6
*ITGAL*	Integrin subunit alpha L
*ABHD17A*	Abhydrolase domain containing 17A
*CHST12*	Carbohydrate sulfotransferase 12
*NBEAL2*	Neurobeachin like 2
*ADAM8*	ADAM metallopeptidase domain 8
*SLC1A7*	Solute carrier family 1 member 7
*LTBP4*	Latent transforming growth factor beta binding protein 4
*CRIP1*	Cysteine rich protein 1
*RNF166*	Ring finger protein 166
*MXD4*	MAX dimerization protein 4
*TNFSF9*	TNF superfamily member 9
*ZNF683*	Zinc finger protein 683
*CTD-2377D24.8*	
*HNRNPLL*	Heterogeneous nuclear ribonucleoprotein L like
*MPST*	mercaptopyruvate sulfurtransferase
*ATP1A3*	ATPase Na^+^/K^+^ transporting subunit alpha 3
*IFNLR1*	Interferon lambda receptor 1
*PTMS*	Parathymosin
*SLC20A1*	Solute carrier family 20 member 1
*MVD*	Mevalonate diphosphate decarboxylase
*SH3RF2*	SH3 domain containing ring finger 2
*RAPGEF1*	Rap guanine nucleotide exchange factor 1
*TGFB1*	Transforming growth factor beta 1
*AL928654.3*	
*BHLHE40*	Basic helix-loop-helix family member e40
*MAPKAPK2*	Mitogen-activated protein kinase-activated protein kinase 2
*PTPRJ*	Protein tyrosine phosphatase, receptor type J
*DGKQ*	Diacylglycerol kinase theta
*MYO3B*	Myosin IIIB
*DUSP8*	Dual specificity phosphatase 8
*FLNA*	Filamin A
*NOP14-AS1*	NOP14 antisense RNA 1
*ITGB2*	Integrin subunit beta 2
*GNG2*	G protein subunit gamma 2
*MSC*	Musculin
*ARHGAP10*	Rho GTPase activating protein 10
*DNMBP*	Dynamin binding protein
*MYO1G*	Myosin IG
*DDN-AS1*	DDN and PRKAG1 antisense RNA 1
*SIPA1*	Signal-induced proliferation-associated 1
*AC093616.1*	Anaphase-promoting complex subunit 1-like
*CTSW*	Cathepsin W
*PXN*	Paxillin
*SSBP3*	Single-stranded DNA binding protein 3
*SLC2A1*	Solute carrier family 2 member 1
*MCOLN2*	Mucolipin 2
*NAA50*	N(alpha)-acetyltransferase 50, NatE catalytic subunit
*RDH10*	Retinol dehydrogenase 10
*NFATC2*	Nuclear factor of activated T cells 2
*KDM4B*	Lysine demethylase 4B
*GALNT10*	Polypeptide N-acetylgalactosaminyltransferase 10
*DPY19L1P1*	DPY19L1 pseudogene 1
*INSIG1*	Insulin induced gene 1
*PLEKHA2*	Pleckstrin homology domain containing A2
*PROK2*	Prokineticin 2
*PTP4A2*	Protein tyrosine phosphatase type IVA, member 2
*GPR27*	G protein-coupled receptor 27
*LINC00355*	Long intergenic non-protein coding RNA 355

The table demonstrates the genes that overlap between the two analytical stages and have a significantly higher expression in CD8+ T cells of patients with dermatomyositis than in those with polymyositis. A false discovery rate threshold of 5% based on the method of Benjamini-Hochberg was used to identify significant differentially expressed genes

To identify enriched biological processes and pathways for the 176 genes that were differentially expressed between PM and DM, the GO database was used. Using Fisher’s exact test with FDR correction (< 0.05), the enriched GO biological processes included lymphocyte migration and regulation of T-cell differentiation (Table 4 and Additional file 5: Table S10).

**Table 4:** Significant Gene Ontology (GO) biological processes in CD8+ T cells of polymyositis and dermatomyositis patients

GO biological process complete	Fold enrichment	*p* value	FDR
Lymphocyte migration (GO:0072676)	11.50	1.05E-04	4.19E-02
Regulation of T-cell differentiation (GO:0045580)	10.10	4.82E-07	2.50E-03
Regulation of lymphocyte differentiation (GO:0045619)	8.31	2.24E-06	3.88E-03
Myeloid leukocyte migration (GO:0097529)	8.17	1.26E-04	4.69E-02
Response to transforming growth factor beta (GO:0071559)	6.73	1.11E-04	4.31E-02
Regulation of leukocyte differentiation (GO:1902105)	5.48	2.05E-05	1.68E-02
Regulation of T cell activation (GO:0050863)	5.26	4.48E-06	6.34E-03
Leukocyte migration (GO:0050900)	5.04	2.79E-06	4.35E-03
Positive regulation of GTPase activity (GO:0043547)	4.42	1.10E-05	1.15E-02
Positive regulation of cell adhesion (GO:0045785)	4.26	3.41E-05	2.41E-02
Positive regulation of MAPK cascade (GO:0043410)	3.85	1.11E-05	1.08E-02
Regulation of GTPase activity (GO:0043087)	3.75	5.71E-05	3.18E-02
Regulation of cell activation (GO:0050865)	3.54	2.88E-05	2.24E-02
Regulation of leukocyte activation (GO:0002694)	3.51	5.87E-05	3.05E-02
Transmembrane receptor protein tyrosine kinase signaling pathway (GO:0007169)	3.47	1.21E-04	4.61E-02
Regulation of MAPK cascade (GO:0043408)	3.42	6.96E-06	8.34E-03
Regulation of cell adhesion (GO:0030155)	3.27	6.93E-05	3.27E-02
Enzyme linked receptor protein signaling pathway (GO:0007167)	3.08	7.94E-05	3.53E-02
Cell migration (GO:0016477)	2.91	3.48E-05	2.35E-02
Positive regulation of immune system process (GO:0002684)	2.88	9.42E-06	1.05E-02
Regulation of immune system process (GO:0002682)	2.75	2.77E-07	2.16E-03
Positive regulation of intracellular signal transduction (GO:1902533)	2.75	4.75E-05	2.84E-02
Regulation of immune response (GO:0050776)	2.70	3.87E-05	2.51E-02
Positive regulation of catalytic activity (GO:0043085)	2.45	3.19E-05	2.36E-02
Immune response (GO:0006955)	2.41	5.32E-06	6.91E-03
Cell surface receptor signaling pathway (GO:0007166)	2.31	5.02E-07	1.95E-03
Positive regulation of signal transduction (GO:0009967)	2.31	6.66E-05	3.24E-02
Positive regulation of molecular function (GO:0044093)	2.24	7.60E-05	3.48E-02
Regulation of intracellular signal transduction (GO:1902531)	2.22	3.92E-05	2.44E-02
Immune system process (GO:0002376)	2.21	8.12E-07	2.11E-03
Regulation of multicellular organismal development (GO:2000026)	2.15	8.34E-05	3.61E-02
Positive regulation of response to stimulus (GO:0048584)	2.14	1.65E-05	1.51E-02
Regulation of catalytic activity (GO:0050790)	2.12	1.83E-05	1.59E-02
Regulation of signal transduction (GO:0009966)	2.01	2.08E-06	4.05E-03
Regulation of developmental process (GO:0050793)	2.00	5.79E-05	3.11E-02
Regulation of signaling (GO:0023051)	2.00	5.40E-07	1.68E-03
Regulation of cell communication (GO:0010646)	1.99	8.44E-07	1.88E-03
Regulation of response to stimulus (GO:0048583)	1.91	2.14E-07	3.33E-03
Regulation of multicellular organismal process (GO:0051239)	1.91	6.12E-05	3.08E-02
Regulation of molecular function (GO:0065009)	1.84	8.55E-05	3.60E-02
Signaling (GO:0023052)	1.58	5.65E-05	3.26E-02
Cell communication (GO:0007154)	1.55	9.36E-05	3.84E-02

*GO* Gene ontology

Significant GO biological processes for the differentially expressed genes in CD8+ T cells of patients with dermatomyositis and patients with polymyositis. Fisher’s exact test with false discovery rate correction (< 0.05) was used to determine significant biological processes. The genes mapped to each GO can be found in Additional file 5: Table S10

### Differential gene expression in CD4+ T cells of HLA-DRB1*03-positive and -negative myositis patients

**Fig. 5:**
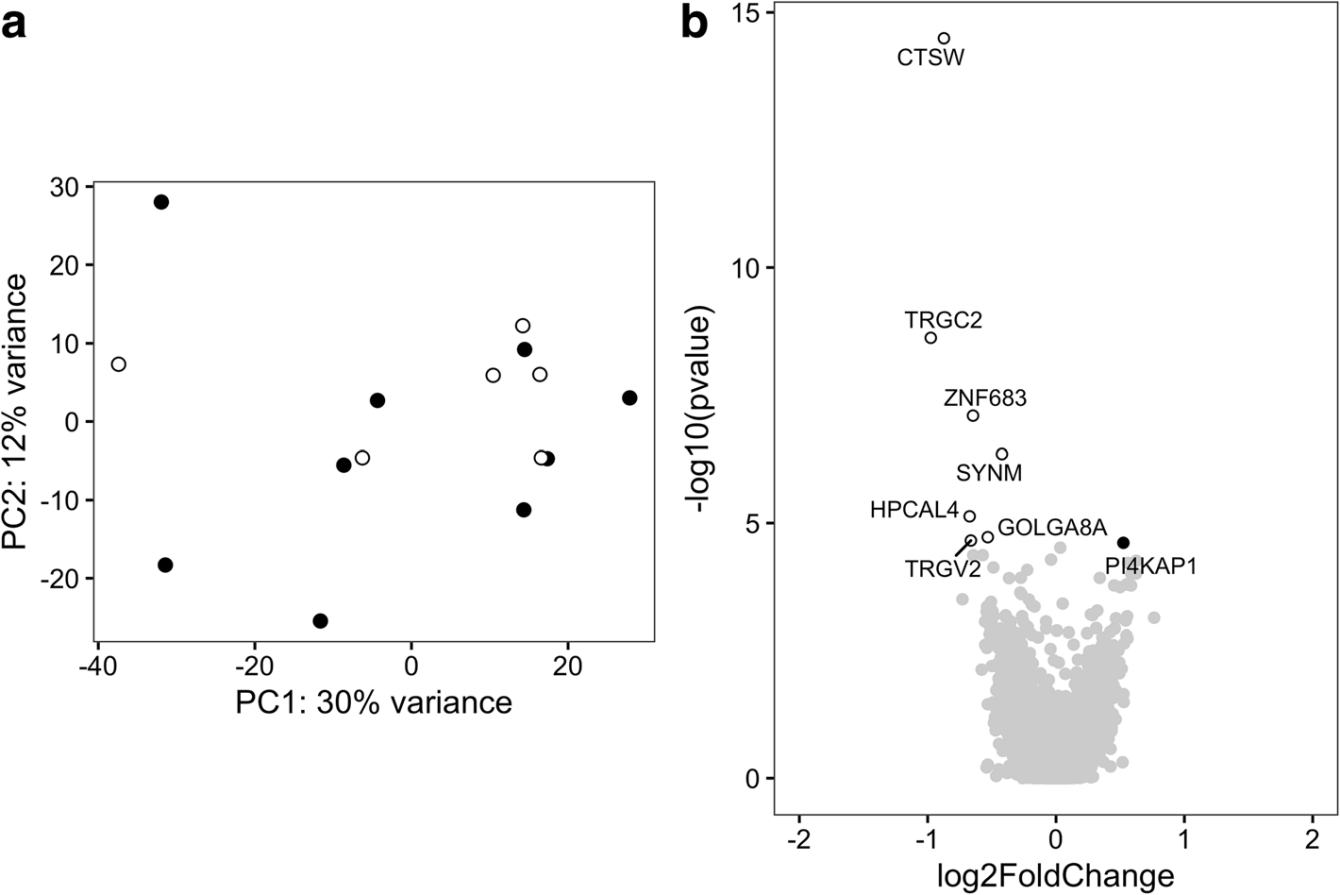
Gene expression profile in CD4+ T cells of HLA-DRB1*03-positive and -negative patients with myositis. **a** Principal components (PCs) 1 and 2 plotted according to the HLA-DRB1*03 status of the patients in a dataset of 21,008 genes (*n* = 15). Samples from HLA-DRB1*03-positive patients with myositis are represented by *filled circles*, and those from HLA-DRB1*03-negative patients with myositis are represented by *open circles*. **b** Differential genome-wide transcriptomic profile in CD4+ T cells for the contrast between HLA-DRB1*03-positive and -negative patients with myositis. The fold changes (log_2_) are shown on the *x*-axis, and the *p* values (−log_10_) are shown on the *y*-axis. The genes that are expressed significantly higher in HLA-DRB1*03-positive myositis are shown by *filled circles*, and the genes expressed significantly higher in HLA-DRB1*03-negative myositis are shown by *open circles*. A false discovery rate threshold of 5% based on the method of Benjamini-Hochberg was used to identify significant differentially expressed genes

HLA-DRB1*03 haplotype is the major genetic risk factor for myositis. Therefore, we searched for differentially expressed genes between HLA-DRB1*03-positive and -negative myositis patients in CD4+ T cells. No significant differences were found in patients with HLA-DRB1*03-positive and -negative myositis regarding disease activity measures and laboratory data (Table 1 and Additional file 1: Tables S4 and S5). The gene expression pattern in HLA-DRB1*03-positive and -negative myositis was examined by PCA, but the first PCs did not separate HLA-DRB1*03-positive from HLA-DRB1*03-negative myositis patients (Fig. 5a), suggesting that these patients are similar on a high genomic level. At the first analytical stage, we performed differential expression analysis using DESeq2 with sex, age group, diagnosis, and RIN value as covariates. This resulted in eight genes that were differentially expressed between HLA-DRB1*03-positive and -negative myositis (FDR < 0.05). Of these genes, one had a higher expression in HLA-DRB1*03-positive patients with myositis, and seven had a higher expression in HLA-DRB1*03-negative patients with myositis (Fig. 5b and Additional file 6: Table S11).

**Fig. 6:**
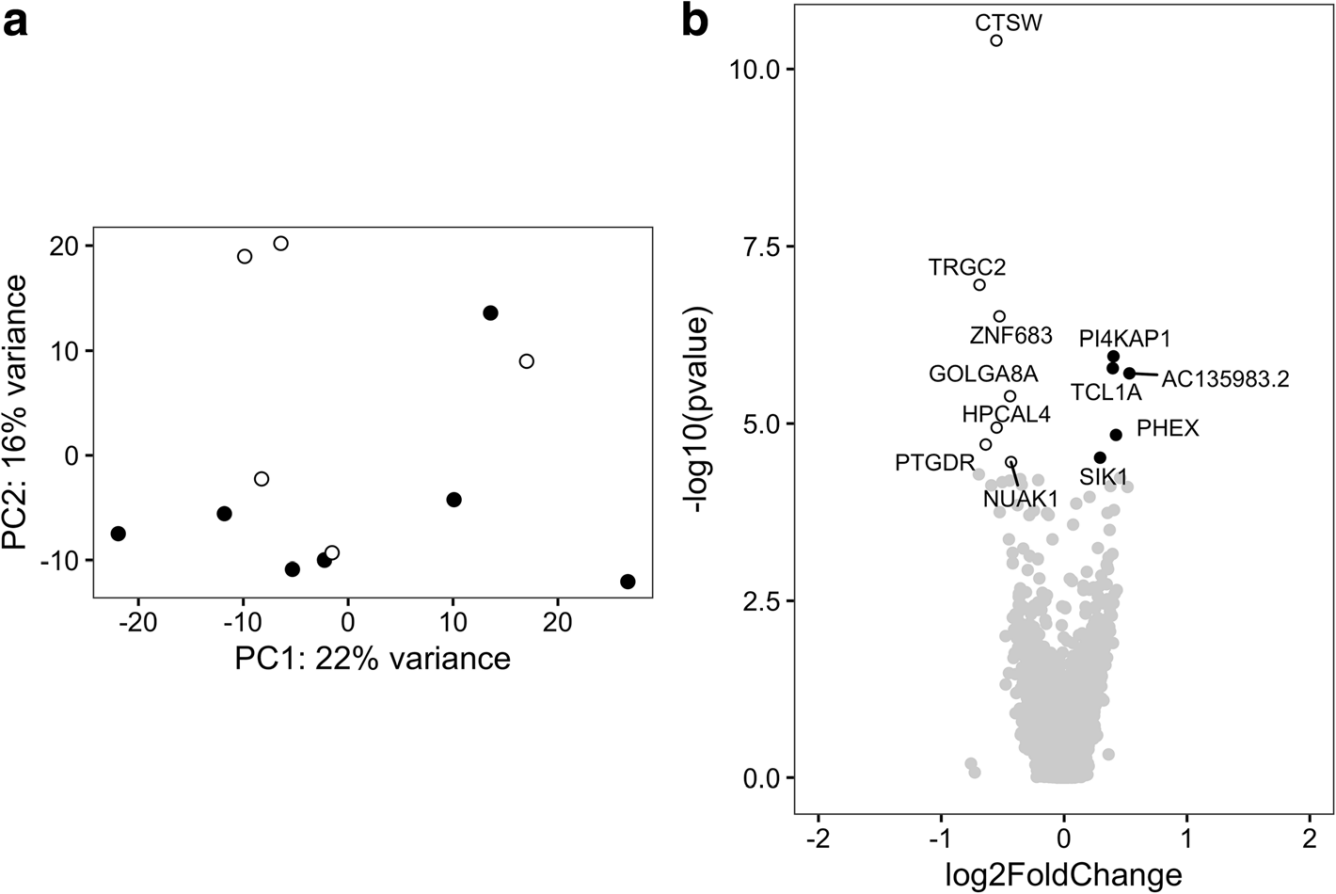
Gene expression profile in CD4+ T cells of HLA-DRB1*03-positive and -negative patients with myositis excluding potential outliers. **a** Principal components (PCs) 1 and 2 plotted according to the HLA-DRB1*03 status of the patients in a dataset of 20,091 genes (*n* = 12). Samples from HLA-DRB1*03-positive patients with myositis are represented by *filled circles*, and those from HLA-DRB1*03-negative patients with myositis are represented by *open circles*. **b** Differential genome-wide transcriptomic profile in CD4+ T cells for the contrast between HLA-DRB1*03-positive and -negative patients with myositis. The fold changes (log_2_) are shown on the *x*-axis, and the *p* values (−log_10_) are shown on the *y*-axis. The genes that are expressed significantly higher in HLA-DRB1*03-positive patients with myositis are shown by *filled circles*, and the genes expressed significantly higher in HLA-DRB1*03-negative patients with myositis are shown by *dark open circles*. A false discovery rate threshold of 5% based on the method of Benjamini-Hochberg was used to identify significant differentially expressed genes

Excluding the potential outliers, which represent higher gene expression levels linked to monocytes, did not affect the PCA (Fig. 6a). At the second analytical stage, we found that although HLA-DRB1*03-positive and -negative myositis are not separated by the first PCs, 12 genes were differentially expressed in CD4+ T cells in comparison of myositis patients with different genotypes. Among these, five genes had a higher expression in CD4+ T cells from HLA-DRB1*03-positive patients with myositis, and eight genes had a higher expression in CD4+ T cells from HLA-DRB1*03-negative patients with myositis (Fig. 6b and Additional file 6: Table S12). Finally, we considered only the genes that were found to be differentially expressed at both analytical stages. *PI4KAP1* was found to have a higher expression in CD4+ T cells of HLA-DRB1*03-positive patients with myositis, and *TRGC2*, *CTSW*, *HPCAL4*, *ZNF683*, and *GOLGA8B* were found to have a higher expression in CD4+ T cells of HLA-DRB1*03-negative patients with myositis.

## Discussion

In our study, we observed significantly more differentially expressed genes in the CD8+ T-cell subset than in the CD4+ T-cell subset when comparing PM and DM patients. In CD8+ T cells, we identified 176 genes that were differentially expressed between PM and DM. In contrast, in CD4+ T cells, only two genes, *ANKRD55* and *S100B*, were found to be differentially expressed between PM and DM. To our knowledge, this is the first study comparing transcriptomic profiles of CD4+ and CD8+ T cells between DM and PM patients. Our data align with the understanding that PM and DM have many common features but differ in genetic architecture and immunohistopathological characteristics. Our findings, together with previous observations, suggest that immune mechanisms related to subpopulations of T cells may significantly vary between these subphenotypes of myositis and also emphasize CD8+ T cells as being of interest both in patients with PM and in those with DM [10, 21, 22].

PM and DM have been modeled as subgroups of myositis in which muscle tissues are infiltrated by T cells, mainly CD8+ T cells in PM and CD4+ T cells in DM [6–9]. More recently, we have demonstrated an overlapping phenotype among the muscle-infiltrating T cells, regardless of their CD4 or CD8 lineage, in that they both display a cytotoxic signature in combination with the absence of the costimulatory CD28 receptor [10, 11]. Such differentiated T cells can also be detected in peripheral blood of patients with PM and DM, reflecting the systemic course of autoimmune disorders [10, 12].

In CD8+ T cells of PM and DM patients, 176 genes were differentially expressed. Interestingly, we noted relatively high expression of *GZMH* and *GZMB* in CD8+ T cells of DM patients compared with CD8+ T cells of PM patients. The protein encoded by *GZMB* is granzyme B and its secretion by CD28^null^ T cells may cause muscle cell damage [11]. Furthermore, granzyme B cleavage sites have been identified in autoantigens, such as FHL1 and HisRS, targeted in autoimmune disorders, including myositis [23, 24]. Moreover, two T-cell receptor (TCR) beta variable genes, *TRBV28* and *TRBV30*, had a higher expression in CD8+ T cells of patients with PM than in patients with DM. TRBV28 has been found to be one of the most common TCR variable segments in muscle tissue of myositis patients carrying the HLA-DRB1*03 allele [25]. This aligns well with the fact that in our analysis of the CD8+ T-cell subset, all PM patients are HLA-DRB1*03-positive and all DM patients are HLA-DRB1*03-negative (Table 1). The TCR beta variable genes are probably differentially expressed due to the HLA status of these patients and might reflect the expansion of pathogenic T-cell clones in this subset of patients. In addition, *TGFB1*, *ZEB2*, and *SMAD7* had a higher expression in CD8+ T cells of patients with DM than in those with PM. This may suggest that transforming growth factor-β signaling [26–29] is upregulated in CD8+ T cells of DM patients compared with PM patients.

In CD4+ T cells, two genes, *ANKRD55* and *S100B*, had a higher expression in PM than in DM. *ANKRD55* encodes ankyrin repeat domain-containing protein 55, which mediates protein-protein interactions [30]. Interestingly, single-nucleotide polymorphisms in this gene have previously been associated with several autoimmune disorders, including rheumatoid arthritis [31–33], Crohn’s disease [34], and multiple sclerosis [35]. A study to reveal the function of this gene in the context of immune function is pending. *S100B* encodes a member of the S100 protein family and is involved in the calcium-dependent regulation of a variety of intracellular activities [36]. S100B is detected in CD8+ T cells and natural killer (NK) cells, but not in CD4+ T cells [37]. This, together with low levels of *S100B* observed in CD4+ T cells in our study, may suggest either that *S100B* expression is evidence of contamination by other cell types or that this expression is characteristic of CD4+ T cells in PM. In any scenario, these data will need replication in an independent group of patients.

The HLA-DRB1*03 haplotype is strongly associated with IIM, especially with PM [2, 38]. We made an effort to investigate how IIM patients with and without this genetic risk factor are different in their transcriptomic profiles in CD4+ T cells. Six genes were differentially expressed in CD4+ T cells of HLA-DRB1*03-positive compared with HLA-DRB1*03-negative myositis patients. We found that *PI4KAP1* had a higher expression in CD4+ T cells of HLA-DRB1*03-positive myositis and that *TRGC2*, *CTSW*, *HPCAL4*, *ZNF683*, and *GOLGA8B* had a higher expression in CD4+ T cells of HLA-DRB1*03-negative myositis patients. Interestingly, we found that *ZNF683* also had a higher expression in CD8+ T cells of HLA-DRB1*03-negative myositis patients when comparing PM and DM, suggesting that the expression of *ZNF683* is common for both subpopulations of T cells. ZNF683 is upregulated in T cells with cytotoxic characteristics [39] and is involved in the transcriptional regulation of effector functions, such as production of interferon-γ and granzyme B [40, 41]. *CTSW* encodes a protein of the cathepsin family, cathepsin W. Cathepsins are found in antigen-presenting cells and are involved in antigen processing [42]. Cathepsin W has been found to be exclusively expressed in CD8+ T cells and NK cells [43]. However, this does not exclude the possibility of differential expression of the transcript in other cell types.

Evidence of differentially expressed genes in whole blood and muscle tissue between various subphenotypes of myositis has been reported previously [22, 44]. These prior investigators found that several type 1 interferon-induced transcripts and proteins were expressed relatively higher in DM patients than in healthy individuals and PM patients. In our study, we did not find type 1 interferon-inducible transcripts to be differentially expressed between PM and DM patients. However, we measured gene expression levels in CD4+ and CD8+ T cells and not in interferon-producing plasmacytoid dendritic cells. In addition, most of the patients in our study were receiving prednisone and additional immunosuppressive drugs that may significantly suppress the type 1 interferon-inducible signature [45].

The limited number of patients with PM and DM, which is the major weakness of our study, did not allow us to consider contribution of autoantibody positivity in the statistical model. The majority of patients in our study had autoantibodies of different specificities (Table 1). It has been shown that anti-MDA5 antibodies are associated with DM complicated by rapidly progressive interstitial lung disease (ILD) [46]. In addition, anti-TIF1-γ antibodies have been associated with cancer-associated DM [47]. Furthermore, anti-Jo-1 antibodies are strongly associated with a clinical phenotype named anti-synthetase syndrome, characterized by ILD, arthritis, mechanic’s hands, and myositis [48]. Further studies with a high number of myositis patients are needed to address correlations between transcriptomic profile and autoantibodies. Moreover, the differences in gene expression levels need to be confirmed at the protein level and in further functional studies.

## Conclusions

In the current study, we analyzed, for the first time to our knowledge, the transcriptomic profiles of different subpopulations of T cells in patients with PM or DM and could demonstrate that these two clinical phenotypes differ regarding T-cell phenotypes related to gene expression. It is evident that these differences are more profound for CD8+ T cells when comparing PM patients with DM patients. Although the differentially expressed genes will need to be confirmed in a larger group of patients, these alterations in the transcriptomes of PM and DM patients suggest different immune mechanisms involved in different subphenotypes of IIM.

## Additional files

Additional file 1: Additional clinical characteristics of patients included in this study. Tables S1–S3 provide additional clinical characteristics of patients included in this study regarding PM and DM. Table S1 includes all patients, whereas, Tables S2 and S3 contain the data for the CD4+ and CD8+ T-cell subsets, respectively. Tables S4 and S5 provide additional clinical characteristics of patients included in this study regarding HLA-DRB1*03 status. Table S4 includes all patients, whereas Table S5 contains the data for the CD4+ T-cell subset. (DOCX 24 kb)

Additional file 2: Differentially expressed genes for CD4+ T cells of PM and DM patients. Table S6 and S7 provide differentially expressed genes for CD4+ T cells of PM and DM patients at analytical stage 1 (including potential outliers) and analytical stage 2 (excluding potential outliers), respectively. (DOCX 18 kb)

Additional file 3: Clustering heat map showing cellular heterogeneity in CD4+ and CD8+ T-cell subsets. Figure S1 shows a clustering heat map indicating cellular heterogeneity in CD4+ and CD8+ T-cell subsets, which indicates minor contamination of other cell types in these subsets. (DOCX 290 kb)

Additional file 4: Differentially expressed genes for CD8+ T cells of PM and DM patients. Tables S8 and S9 provide differentially expressed genes for CD8+ T cells of PM and DM patients at analytical stage 1 (including potential outliers) and analytical stage 2 (excluding potential outliers), respectively. (DOCX 106 kb)

Additional file 5: Gene Ontology biological processes for the differentially expressed genes in CD8+ T cells of PM and DM patients. Table S10 shows the genes mapped to the enriched GO biological processes for the differentially expressed genes in CD8+ T cells of PM and DM patients. (DOCX 17 kb)

Additional file 6: Differentially expressed genes in CD4+ T cells of HLA-DRB1*03-positive and -negative myositis patients. Table S11 and S12 provide differentially expressed genes for CD4+ T cells of HLA-DRB1*03-positive and -negative myositis patients at analytical stage 1 (including potential outliers) and analytical stage 2 (excluding potential outliers), respectively. (DOCX 16 kb)

## Abbreviations

DM: Dermatomyositis
FDR: False discovery rate
GO: Gene Ontology
IIM: Idiopathic inflammatory myopathy
ILD: Interstitial lung disease
NK: Natural killer
PBMC: Peripheral blood mononuclear cell
PC: Principal component
PCA: Principal component analysis
PM: Polymyositis
RIN: RNA integrity number
SNP: Single-nucleotide polymorphism
TCR: T-cell receptor
TGF: Transforming growth factor
