# Supplementary material for: T-cell transcriptomics from peripheral blood highlights differences between polymyositis and dermatomyositis patients
Source: Arthritis Res Ther. 2018 Aug 29;20:188. doi: 10.1186/s13075-018-1688-7 (PMC6116372; doi:10.1186/s13075-018-1688-7)
Supplement: Supplementary file 1 — Additional clinical characteristics of patients included in this study. Tables S1–S3 provide additional clinical characteristics of patients included in this study regarding PM and DM. Table S1 includes all patients, whereas, Tables S2 and S3 contain the data for the CD4+ and CD8+ T-cell subsets, respectively. Tables S4 and S5 provide additional clinical characteristics of patients included in this study regarding HLA-DRB1*03 status. Table S4 includes all patients, whereas Table S5 contains the data for the CD4+ T-cell subset. (DOCX 24 kb) [file 13075_2018_1688_MOESM1_ESM.docx]

**Table S1**

Additional clinical characteristics of all patients with myositis enrolled in the study at time of blood sampling. Values are presented as median (IQR). P-values were estimated using the Mann-Whitney U test. P-values of <0.05 were considered as significant. Abbreviations: ALT: Alanine aminotransferase (normal levels: 0-0.58 μkat/L), AST: Aspartate aminotransferase (normal levels: 0-0.58 μkat/L), CK: Creatine kinase (normal level: 0.6-3.5 μkat/L), CRP: C reactive protein, DM: Dermatomyositis, ESR: Erythrocyte sedimentation rate, HAQ: Health assessment questionnaire, IQR: Interquartile range, LDH: Lactate dehydrogenase (normal level: <3.5 μkat/L), MMT-8: Manual muscle test-8, PM: Polymyositis, VAS: visual analogue scale.

| **Variable** | **PM (n=20)** | **DM (n=13)** | **p-value** |
| --- | --- | --- | --- |
| Disease duration (years) | 16 (10.75) | 12 (6) | 0.337 |
| Patient's global disease activity assessment, VAS (100 mm) | 46.50 (40.50) | 18 (58) | 0.561 |
| Physician's global disease activity assessment, VAS (100 mm) | 10 (17.50) | 0 (10) | 0.316 |
| MMT-8 (0-80) | 71 (9.50) | 79 (9) | 0.054 |
| HAQ (0-3) | 1.13 (0.62) | 0.63 (1.37) | 0.284 |
| CK (μkat/L) | 1.50 (1.45) | 1.40 (0.58) | 0.338 |
| LDH (μkat/L) | 3.10 (1.10) | 2.90 (0.85) | 0.376 |
| ALT (μkat/L) | 0.35 (0.21) | 0.29 (0.34) | 0.901 |
| AST (μkat/L) | 0.41 (0.22) | 0.40 (0.06) | 0.655 |
| Extramuscular global assessment, VAS (100 mm) | 0 (10) | 0 (2.50) | 0.624 |
| ESR (mm/hr) | 13 (8) | 30 (49.50) | 0.240 |
| CRP (mg/L) | 2.5 (7.75) | 2 (26) | 0.622 |

**Table S2**

Additional clinical characteristics of patients with myositis in the CD4+ T cell subset at time of blood sampling. Values are presented as median (IQR). P-values were estimated using the Mann-Whitney U test. P-values of <0.05 were considered as significant. Abbreviations: ALT: Alanine aminotransferase (normal levels: 0-0.58 μkat/L), AST: Aspartate aminotransferase (normal levels: 0-0.58 μkat/L), CK: Creatine kinase (normal level: 0.6-3.5 μkat/L), CRP: C reactive protein, DM: Dermatomyositis, ESR: Erythrocyte sedimentation rate, HAQ: Health assessment questionnaire, IQR: Interquartile range, LDH: Lactate dehydrogenase (normal level: <3.5 μkat/L), MMT-8: Manual muscle test-8, PM: Polymyositis, VAS: visual analogue scale.

| **Variable** | **PM (n=8)** | **DM (n=7)** | **p-value** |
| --- | --- | --- | --- |
| Disease duration (years) | 12 (8) | 9 (4.50) | 0.684 |
| Patient's global disease activity assessment, VAS (100 mm) | 52 (26.75) | 39 (50.50) | 0.366 |
| Physician's global disease activity assessment, VAS (100 mm) | 10 (15) | 0 (7.50) | 0.491 |
| MMT-8 (0-80) | 69.50 (9) | 71 (9) | 0.222 |
| HAQ (0-3) | 1.07 (0.41) | 0.88 (1.00) | 0.720 |
| CK (μkat/L) | 1.40 (1) | 1.30 (1.35) | 0.748 |
| LDH (μkat/L) | 3.10 (0.65) | 3.25 (0.78) | 0.773 |
| ALT (μkat/L) | 0.37 (0.13) | 0.29 (0.28) | 1 |
| AST (μkat/L) | 0.37 (0.16) | 0.43 (0.09) | 0.701 |
| Extramuscular global assessment, VAS (100 mm) | 0 (10) | 0 (5) | 0.653 |
| ESR (mm/hr) | 15 (22) | 42 (48.50) | 0.575 |
| CRP (mg/L) | 2 (4.50) | 3 (22) | 0.423 |

**Table S3**

Additional clinical characteristics of patients with myositis in the CD8+ T cell subset at time of blood sampling. Values are presented as median (IQR). P-values were estimated using the Mann-Whitney U test. P-values of <0.05 were considered as significant. Abbreviations: ALT: Alanine aminotransferase (normal levels: 0-0.58 μkat/L), AST: Aspartate aminotransferase (normal levels: 0-0.58 μkat/L), CK: Creatine kinase (normal level: 0.6-3.5 μkat/L), CRP: C reactive protein, DM: Dermatomyositis, ESR: Erythrocyte sedimentation rate, HAQ: Health assessment questionnaire, IQR: Interquartile range, LDH: Lactate dehydrogenase (normal level: <3.5 μkat/L), MMT-8: Manual muscle test-8, PM: Polymyositis, VAS: visual analogue scale.

| **Variable** | **PM (n=4)** | **DM (n=5)** | **p-value** |
| --- | --- | --- | --- |
| Disease duration (years) | 12.50 (11.75) | 12 (3) | 0.905 |
| Patient's global disease activity assessment, VAS (100 mm) | 47.50 (8.75) | 18 (22) | 0.286 |
| Physician's global disease activity assessment, VAS (100 mm) | 5 (12.50) | 0 (5) | 0.590 |
| MMT-8 (0-80) | 72.50 (4.25) | 71 (7) | 0.709 |
| HAQ (0-3) | 1.19 (0.44) | 0.25 (1.13) | 0.389 |
| CK (μkat/L) | 1.50 (0.55) | 1.80 (2.18) | 0.858 |
| LDH (μkat/L) | 3 (0.30) | 2.70 (0.55) | 0.700 |
| ALT (μkat/L) | 0.35 (0.11) | 0.27 (0.36) | 0.786 |
| AST (μkat/L) | 0.28 (0.09) | 0.42 (0.05) | 0.285 |
| Extramuscular global assessment, VAS (100 mm) | 5 (10) | 0 (0) | 0.128 |
| ESR (mm/hr) | 31 (11) | 56.50 (16.75) | 0.400 |
| CRP (mg/L) | 8 (5) | 18 (24) | 0.453 |

**Table S4**

Additional clinical characteristics of all patients with myositis enrolled in the study at time of blood sampling. Values are presented as median (IQR). P-values were estimated using the Mann-Whitney U test. P-values of <0.05 were considered as significant. Abbreviations: ALT: Alanine aminotransferase (normal levels: 0-0.58 μkat/L), AST: Aspartate aminotransferase (normal levels: 0-0.58 μkat/L), CK: Creatine kinase (normal level: 0.6-3.5 μkat/L), CRP: C reactive protein, ESR: Erythrocyte sedimentation rate, HAQ: Health assessment questionnaire, IQR: Interquartile range, LDH: Lactate dehydrogenase (normal level: <3.5 μkat/L), MMT-8: Manual muscle test-8, VAS: visual analogue scale.

| **Variable** | ***HLA-DRB1*03* positive (n=19)** | ***HLA-DRB1*03* negative (n=14)** | **p-value** |
| --- | --- | --- | --- |
| Disease duration (years) | 13 (10) | 15 (9.75) | 0.104 |
| Patient's global disease activity assessment, VAS (100 mm) | 38.50 (41.50) | 46 (61) | 0.483 |
| Physician's global disease activity assessment, VAS (100 mm) | 10 (17.50) | 0 (10) | 0.358 |
| MMT-8 (0-80) | 75 (9) | 71 (8.50) | 0.279 |
| HAQ (0-3) | 1.07 (0.94) | 1.01 (1.28) | 0.882 |
| CK (μkat/L) | 1.50 (1.05) | 1.30 (1) | 0.546 |
| LDH (μkat/L) | 3.05 (1.20) | 3.05 (0.78) | 0.538 |
| ALT (μkat/L) | 0.35 (0.19) | 0.29 (0.34) | 0.970 |
| AST (μkat/L) | 0.39 (0.21) | 0.40 (0.06) | 0.794 |
| Extramuscular global assessment, VAS (100 mm) | 0 (10) | 0 (0) | 0.142 |
| ESR (mm/hr) | 12.50 (24) | 17 (40) | 0.072 |
| CRP (mg/L) | 2 (7) | 4 (15) | 0.329 |

**Table S5**

Additional clinical characteristics of patients with myositis in the CD4+ T cell subset at time of blood sampling. Values are presented as median (IQR). P-values were estimated using the Mann-Whitney U test. P-values of <0.05 were considered as significant. Abbreviations: ALT: Alanine aminotransferase (normal levels: 0-0.58 μkat/L), AST: Aspartate aminotransferase (normal levels: 0-0.58 μkat/L), CK: Creatine kinase (normal level: 0.6-3.5 μkat/L), CRP: C reactive protein, ESR: Erythrocyte sedimentation rate, HAQ: Health assessment questionnaire, IQR: Interquartile range, LDH: Lactate dehydrogenase (normal level: <3.5 μkat/L), MMT-8: Manual muscle test-8, VAS: visual analogue scale.

| **Variable** | ***HLA-DRB1*03* positive (n=9)** | ***HLA-DRB1*03* negative (n=6)** | **p-value** |
| --- | --- | --- | --- |
| Disease duration (years) | 8 (8) | 12 (4.50) | 0.343 |
| Patient's global disease activity assessment, VAS (100 mm) | 46.50 (26.75) | 46 (49) | 0.622 |
| Physician's global disease activity assessment, VAS (100 mm) | 10 (10) | 0 (5) | 0.565 |
| MMT-8 (0-80) | 74 (10) | 70 (3.50) | 0.678 |
| HAQ (0-3) | 0.94 (0.60) | 1.13 (0.87) | 0.462 |
| CK (μkat/L) | 1.70 (1.03) | 1.30 (1.35) | 0.847 |
| LDH (μkat/L) | 3.05 (0.38) | 3.60 (0.80) | 0.883 |
| ALT (μkat/L) | 0.40 (0.18) | 0.33 (0.28) | 1 |
| AST (μkat/L) | 0.39 (0.19) | 0.41 (0.06) | 1 |
| Extramuscular global assessment, VAS (100 mm) | 0 (10) | 0 (0) | 0.532 |
| ESR (mm/hr) | 10 (17) | 55 (37) | 0.074 |
| CRP (mg/L) | 2 (3.50) | 10.50 (24) | 0.145 |
