# Supplementary material for: T-cell transcriptomics from peripheral blood highlights differences between polymyositis and dermatomyositis patients
Source: Arthritis Res Ther. 2018 Aug 29;20:188. doi: 10.1186/s13075-018-1688-7 (PMC6116372; doi:10.1186/s13075-018-1688-7)
Supplement: Supplementary file 2 — Differentially expressed genes for CD4+ T cells of PM and DM patients. Table S6 and S7 provide differentially expressed genes for CD4+ T cells of PM and DM patients at analytical stage 1 (including potential outliers) and analytical stage 2 (excluding potential outliers), respectively. (DOCX 18 kb) [file 13075_2018_1688_MOESM2_ESM.docx]

**Table S6**

Differentially expressed genes in CD4+ T cells of PM and DM patients. Genes with a positive FC are higher expressed in PM patients and genes with a negative FC are higher expressed in DM patients. P-values were estimated by the Wald test in DESeq2 adjusted for gender, age group, *HLA-DRB1**03 status, and RIN value. A FDR threshold of 5% based on the method of Benjamini-Hochberg was used to identify significant differentially expressed genes. Abbreviations: FC: Fold Change.

| **Gene symbol** | **Gene name** | **Log2FC** | **P-value** | **P-adjusted** |
| --- | --- | --- | --- | --- |
| P2RY14 | purinergic receptor P2Y14 | -0.89 | 1.28E-05 | 2.23E-02 |
| GCNT1 | glucosaminyl (N-acetyl) transferase 1, core 2 | -0.85 | 3.25E-05 | 4.33E-02 |
| CLIC5 | chloride intracellular channel 5 | -0.78 | 8.41E-06 | 1.82E-02 |
| ASS1P1 | argininosuccinate synthetase 1 pseudogene 1 | -0.75 | 7.81E-06 | 1.82E-02 |
| HSPA7 | heat shock protein family A (Hsp70) member 7 | -0.74 | 6.36E-09 | 1.10E-04 |
| FCGR2C | Fc fragment of IgG receptor IIc (gene/pseudogene) | -0.69 | 3.25E-06 | 1.13E-02 |
| HLA-F | major histocompatibility complex, class I, F | -0.4 | 1.17E-05 | 2.23E-02 |
| CCNB1IP1 | cyclin B1 interacting protein 1 | 0.53 | 3.83E-05 | 4.74E-02 |
| CTSW | cathepsin W | 0.59 | 9.97E-08 | 5.80E-04 |
| RPS2P5 | ribosomal protein S2 pseudogene 5 | 0.61 | 3.21E-05 | 4.33E-02 |
| SNORA5C | small nucleolar RNA,H/ACA box 5C | 0.63 | 5.43E-06 | 1.57E-02 |
| S100B | S100 calcium binding protein B | 0.67 | 2.24E-06 | 9.71E-03 |
| ANKRD55 | ankyrin repeat domain 55 | 0.89 | 3.36E-08 | 2.90E-04 |

**Table S7**

Differentially expressed genes in CD4+ T cells of PM and DM patients excluding potential outliers. Genes with a positive FC are higher expressed in PM patients and genes with a negative FC are higher expressed in DM patients. P-values were estimated by the Wald test in DESeq2 adjusted for gender, age group, *HLA-DRB1**03 status, and RIN value. A FDR threshold of 5% based on the method of Benjamini-Hochberg was used to identify significant differentially expressed genes. Abbreviations: FC: Fold Change.

| **Gene symbol** | **Gene name** | **Log2FC** | **P-value** | **P-adjusted** |
| --- | --- | --- | --- | --- |
| CCDC50 | coiled-coil domain containing 50 | -0.46 | 7.02E-06 | 2.57E-02 |
| LRRC37A4P | leucine rich repeat containing 37 member A4, pseudogene | -0.45 | 4.70E-06 | 2.29E-02 |
| ANKRD55 | ankyrin repeat domain 55 | 0.54 | 3.30E-06 | 2.29E-02 |
| S100B | S100 calcium binding protein B | 0.6 | 4.16E-09 | 6.00E-05 |
