# Supplementary material for: T-cell transcriptomics from peripheral blood highlights differences between polymyositis and dermatomyositis patients
Source: Arthritis Res Ther. 2018 Aug 29;20:188. doi: 10.1186/s13075-018-1688-7 (PMC6116372; doi:10.1186/s13075-018-1688-7)
Supplement: Supplementary file 3 — Clustering heat map showing cellular heterogeneity in CD4+ and CD8+ T-cell subsets. Figure S1 shows a clustering heat map indicating cellular heterogeneity in CD4+ and CD8+ T-cell subsets, which indicates minor contamination of other cell types in these subsets. (DOCX 290 kb) [file 13075_2018_1688_MOESM3_ESM.docx]

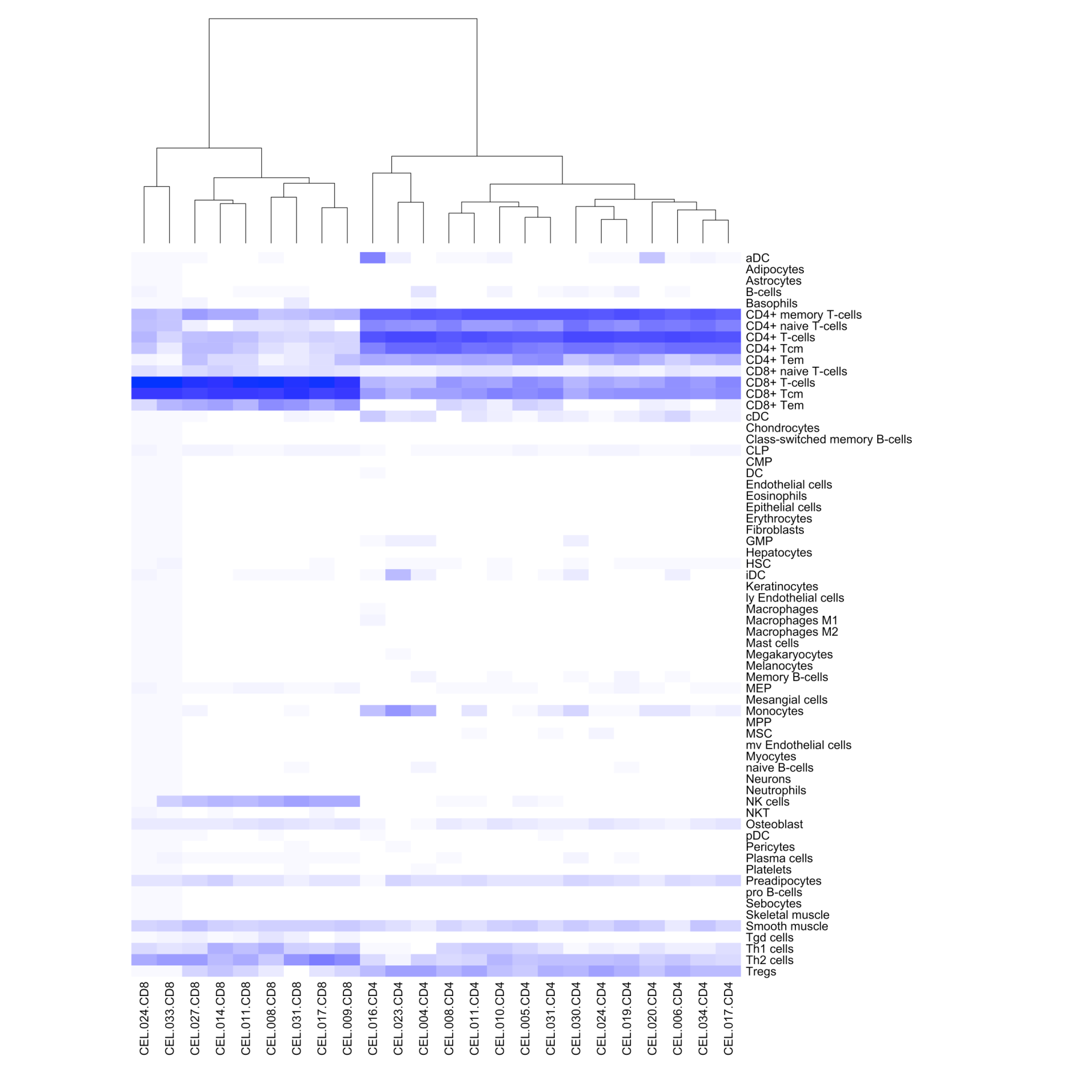


*

*

*

*

**Figure S1**

Clustering heatmap showing cellular heterogeneity in CD4+ and CD8+ T cell subsets. The xCell tool [13] was used to identify cellular heterogeneity in the CD4+ and CD8+ cell subset from gene expression data. Despite evidence of minor contamination of other cell types, CD4+ and CD8+ T cells are clearly discriminated. Color intensity indicates cell type enrichment. The samples marked with an asterisk are the potential outliers which were excluded from the analysis at analytic stage 2.
