# Supplementary material for: T-cell transcriptomics from peripheral blood highlights differences between polymyositis and dermatomyositis patients
Source: Arthritis Res Ther. 2018 Aug 29;20:188. doi: 10.1186/s13075-018-1688-7 (PMC6116372; doi:10.1186/s13075-018-1688-7)
Supplement: Supplementary file 4 — Differentially expressed genes for CD8+ T cells of PM and DM patients. Tables S8 and S9 provide differentially expressed genes for CD8+ T cells of PM and DM patients at analytical stage 1 (including potential outliers) and analytical stage 2 (excluding potential outliers), respectively. (DOCX 106 kb) [file 13075_2018_1688_MOESM4_ESM.docx]

**Table S8**

Differentially expressed genes in CD8+ T cells of PM and DM patients. Genes with a positive FC are higher expressed in PM patients and genes with a negative FC are higher expressed in DM patients. P-values were estimated by the Wald test in DESeq2 adjusted for gender, age group, and RIN value. A FDR threshold of 5% based on the method of Benjamini-Hochberg was used to identify significant differentially expressed genes. Abbreviations: FC: Fold Change.

| **Gene symbol** | **Gene name** | **Log2FC** | **P-value** | **P-adjusted** |
| --- | --- | --- | --- | --- |
| RP4-765C7.1 | ribosomal protein S14 pseudogene 2 | -3.71 | 3.55E-16 | 1.10E-12 |
| RP11-343H5.1 | ribosomal protein S14 pseudogene 14 | -2.34 | 1.67E-13 | 2.15E-10 |
| NKG7 | natural killer cell granule protein 7 | -1.66 | 1.05E-20 | 8.12E-17 |
| TGFBR3 | transforming growth factor beta receptor 3 | -1.52 | 1.87E-23 | 2.89E-19 |
| GZMH | granzyme H | -1.48 | 1.00E-16 | 3.86E-13 |
| EFHD2 | EF-hand domain family member D2 | -1.45 | 2.42E-18 | 1.24E-14 |
| ZEB2 | zinc finger E-box binding homeobox 2 | -1.44 | 4.41E-16 | 1.13E-12 |
| KIAA1671 | KIAA1671 | -1.44 | 1.47E-13 | 2.07E-10 |
| SETBP1 | SET binding protein 1 | -1.44 | 2.70E-14 | 5.22E-11 |
| SLC2A14 | solute carrier family 2 member 14 | -1.37 | 1.37E-06 | 2.22E-04 |
| FAM118A | family with sequence similarity 118 member A | -1.37 | 8.89E-07 | 1.61E-04 |
| ADGRG1 | adhesion G protein-coupled receptor G1 | -1.36 | 2.78E-13 | 3.30E-10 |
| ADRB2 | adrenoceptor beta 2 | -1.35 | 1.32E-13 | 2.03E-10 |
| CACNA2D2 | calcium voltage-gated channel auxiliary subunit alpha2delta 2 | -1.33 | 5.67E-14 | 9.72E-11 |
| PDGFD | platelet derived growth factor D | -1.33 | 1.70E-11 | 1.75E-08 |
| SH3TC1 | SH3 domain and tetratricopeptide repeats 1 | -1.32 | 6.38E-10 | 3.94E-07 |
| PRSS23 | serine protease 23 | -1.31 | 5.46E-15 | 1.20E-11 |
| TBKBP1 | TBK1 binding protein 1 | -1.30 | 1.99E-10 | 1.71E-07 |
| AC009951.1 |  | -1.27 | 3.80E-09 | 1.83E-06 |
| RAB11FIP5 | RAB11 family interacting protein 5 | -1.26 | 1.08E-11 | 1.19E-08 |
| GNAO1 | G protein subunit alpha o1 | -1.24 | 1.00E-09 | 5.73E-07 |
| MUC16 | mucin 16, cell surface associated | -1.24 | 2.13E-09 | 1.06E-06 |
| RP11-107E5.2 |  | -1.23 | 7.14E-09 | 2.98E-06 |
| KIF19 | kinesin family member 19 | -1.22 | 1.82E-09 | 9.59E-07 |
| CST7 | cystatin F | -1.22 | 7.96E-11 | 7.68E-08 |
| SMAD7 | SMAD family member 7 | -1.19 | 2.15E-07 | 4.88E-05 |
| LINC02086 | long intergenic non-protein coding RNA 2086 | -1.19 | 1.38E-08 | 4.95E-06 |
| AC040970.1 | uncharacterized LOC101927963 | -1.17 | 1.50E-07 | 3.56E-05 |
| LLGL2 | LLGL2, scribble cell polarity complex component | -1.16 | 1.22E-10 | 1.11E-07 |
| SYNE1 | spectrin repeat containing nuclear envelope protein 1 | -1.15 | 5.45E-10 | 3.50E-07 |
| RAP1GAP2 | RAP1 GTPase activating protein 2 | -1.13 | 3.00E-10 | 2.33E-07 |
| FAM53B | family with sequence similarity 53 member B | -1.13 | 3.68E-10 | 2.59E-07 |
| TOGARAM2 | TOG array regulator of axonemal microtubules 2 | -1.13 | 1.33E-08 | 4.95E-06 |
| FRMPD3 | FERM and PDZ domain containing 3 | -1.12 | 5.31E-09 | 2.41E-06 |
| TBX21 | T-box 21 | -1.10 | 3.69E-10 | 2.59E-07 |
| SESN2 | sestrin 2 | -1.10 | 1.18E-07 | 2.93E-05 |
| PAX5 | paired box 5 | -1.10 | 4.22E-04 | 1.76E-02 |
| MIDN | midnolin | -1.10 | 9.20E-07 | 1.64E-04 |
| CCL5 | C-C motif chemokine ligand 5 | -1.09 | 1.53E-08 | 5.37E-06 |
| SYTL3 | synaptotagmin like 3 | -1.08 | 4.42E-10 | 2.96E-07 |
| GAB3 | GRB2 associated binding protein 3 | -1.08 | 1.16E-08 | 4.49E-06 |
| PLCG2 | phospholipase C gamma 2 | -1.08 | 2.82E-05 | 2.46E-03 |
| TTC38 | tetratricopeptide repeat domain 38 | -1.08 | 1.09E-08 | 4.30E-06 |
| TRBV7-4 | T cell receptor beta variable 7-4 (gene/pseudogene) | -1.07 | 8.17E-04 | 2.74E-02 |
| LDLR | low density lipoprotein receptor | -1.07 | 4.62E-07 | 9.13E-05 |
| CCL4 | C-C motif chemokine ligand 4 | -1.06 | 1.19E-09 | 6.55E-07 |
| DMWD | DM1 locus, WD repeat containing | -1.06 | 3.66E-08 | 1.13E-05 |
| CASZ1 | castor zinc finger 1 | -1.04 | 8.00E-07 | 1.49E-04 |
| LAG3 | lymphocyte activating 3 | -1.04 | 7.08E-07 | 1.35E-04 |
| DYRK1B | dual specificity tyrosine phosphorylation regulated kinase 1B | -1.03 | 2.29E-07 | 4.98E-05 |
| GPR153 | G protein-coupled receptor 153 | -1.03 | 1.32E-06 | 2.16E-04 |
| MATK | megakaryocyte-associated tyrosine kinase | -1.02 | 1.25E-07 | 3.07E-05 |
| SH2D2A | SH2 domain containing 2A | -1.01 | 5.85E-09 | 2.58E-06 |
| RHBDF2 | rhomboid 5 homolog 2 | -1.01 | 2.52E-08 | 8.26E-06 |
| GFI1 | growth factor independent 1 transcriptional repressor | -1.01 | 1.07E-07 | 2.86E-05 |
| ADGRG5 | adhesion G protein-coupled receptor G5 | -1.00 | 6.65E-08 | 1.90E-05 |
| CCL4L2 | C-C motif chemokine ligand 4 like 2 | -0.99 | 1.61E-05 | 1.62E-03 |
| FASLG | Fas ligand | -0.99 | 1.04E-06 | 1.79E-04 |
| UBE2Q2P1 | ubiquitin conjugating enzyme E2 Q2 pseudogene 1 | -0.99 | 3.26E-06 | 4.66E-04 |
| GALNT3 | polypeptide N-acetylgalactosaminyltransferase 3 | -0.98 | 1.12E-07 | 2.89E-05 |
| RUNX3 | runt related transcription factor 3 | -0.98 | 5.10E-07 | 9.96E-05 |
| PLA2G16 | phospholipase A2 group XVI | -0.97 | 2.42E-06 | 3.66E-04 |
| SLC15A4 | solute carrier family 15 member 4 | -0.97 | 2.28E-07 | 4.98E-05 |
| PPP2R2B | protein phosphatase 2 regulatory subunit Bbeta | -0.97 | 1.40E-07 | 3.37E-05 |
| RGS9 | regulator of G protein signaling 9 | -0.97 | 2.59E-08 | 8.33E-06 |
| PATL2 | PAT1 homolog 2 | -0.97 | 1.17E-07 | 2.93E-05 |
| TEDC1 | tubulin epsilon and delta complex 1 | -0.96 | 8.06E-04 | 2.72E-02 |
| C1orf21 | chromosome 1 open reading frame 21 | -0.96 | 9.41E-08 | 2.57E-05 |
| S1PR5 | sphingosine-1-phosphate receptor 5 | -0.95 | 2.77E-08 | 8.72E-06 |
| AC110771.1 | uncharacterized LOC651430 | -0.95 | 1.04E-03 | 3.24E-02 |
| DMPK | DM1 protein kinase | -0.95 | 3.33E-05 | 2.74E-03 |
| AJM1 | apical junction component 1 homolog | -0.95 | 5.50E-05 | 4.00E-03 |
| TMCC3 | transmembrane and coiled-coil domain family 3 | -0.95 | 9.54E-09 | 3.88E-06 |
| TRGV7 | T cell receptor gamma variable 7 (pseudogene) | -0.95 | 1.50E-04 | 8.19E-03 |
| TLR3 | toll like receptor 3 | -0.95 | 2.61E-06 | 3.84E-04 |
| AC215219.1 | WASH complex subunit 1 pseudogene | -0.94 | 3.30E-05 | 2.74E-03 |
| B3GAT1 | beta-1,3-glucuronyltransferase 1 | -0.94 | 6.54E-08 | 1.90E-05 |
| RPS14P8 | ribosomal protein S14 pseudogene 8 | -0.94 | 2.33E-04 | 1.11E-02 |
| KIF21A | kinesin family member 21A | -0.93 | 1.44E-06 | 2.32E-04 |
| GLB1L2 | galactosidase beta 1 like 2 | -0.93 | 4.18E-07 | 8.59E-05 |
| PRELID2 | PRELI domain containing 2 | -0.92 | 6.80E-07 | 1.31E-04 |
| SLAMF7 | SLAM family member 7 | -0.92 | 1.12E-06 | 1.88E-04 |
| ADAP1 | ArfGAP with dual PH domains 1 | -0.92 | 2.30E-04 | 1.11E-02 |
| PKN3 | protein kinase N3 | -0.92 | 2.42E-05 | 2.20E-03 |
| TRGJ2 | T cell receptor gamma joining 2 | -0.92 | 1.07E-04 | 6.38E-03 |
| WHRN | whirlin | -0.92 | 3.66E-04 | 1.55E-02 |
| DENND3 | DENN domain containing 3 | -0.92 | 1.31E-05 | 1.34E-03 |
| SOX13 | SRY-box 13 | -0.91 | 4.27E-07 | 8.59E-05 |
| CEP78 | centrosomal protein 78 | -0.91 | 3.85E-06 | 5.44E-04 |
| KCNQ5 | potassium voltage-gated channel subfamily Q member 5 | -0.91 | 1.29E-03 | 3.76E-02 |
| PRF1 | perforin 1 | -0.90 | 1.88E-07 | 4.40E-05 |
| HLA-DPB1 | major histocompatibility complex, class II, DP beta 1 | -0.90 | 3.94E-07 | 8.21E-05 |
| GZMB | granzyme B | -0.90 | 4.81E-08 | 1.43E-05 |
| FGFBP2 | fibroblast growth factor binding protein 2 | -0.90 | 3.88E-08 | 1.18E-05 |
| CLDND2 | claudin domain containing 2 | -0.90 | 9.20E-06 | 1.01E-03 |
| RAP2A | RAP2A, member of RAS oncogene family | -0.89 | 1.12E-07 | 2.89E-05 |
| TMEM63C | transmembrane protein 63C | -0.89 | 1.16E-05 | 1.20E-03 |
| PLEKHF1 | pleckstrin homology and FYVE domain containing 1 | -0.89 | 4.17E-06 | 5.69E-04 |
| TRGC2 | T cell receptor gamma constant 2 | -0.89 | 4.83E-05 | 3.57E-03 |
| FCRL6 | Fc receptor like 6 | -0.89 | 2.40E-08 | 8.05E-06 |
| ITGAL | integrin subunit alpha L | -0.88 | 1.96E-07 | 4.51E-05 |
| CACNB3 | calcium voltage-gated channel auxiliary subunit beta 3 | -0.88 | 1.09E-05 | 1.16E-03 |
| LPCAT1 | lysophosphatidylcholine acyltransferase 1 | -0.88 | 1.01E-05 | 1.10E-03 |
| ABHD17A | abhydrolase domain containing 17A | -0.88 | 4.11E-06 | 5.66E-04 |
| CLCF1 | cardiotrophin like cytokine factor 1 | -0.88 | 1.76E-05 | 1.73E-03 |
| SGSM1 | small G protein signaling modulator 1 | -0.87 | 5.03E-06 | 6.49E-04 |
| JAZF1 | JAZF zinc finger 1 | -0.87 | 2.10E-06 | 3.24E-04 |
| CHST12 | carbohydrate sulfotransferase 12 | -0.86 | 8.26E-05 | 5.24E-03 |
| NBEAL2 | neurobeachin like 2 | -0.86 | 8.92E-08 | 2.50E-05 |
| ADAM8 | ADAM metallopeptidase domain 8 | -0.86 | 2.74E-06 | 3.97E-04 |
| ST3GAL4 | ST3 beta-galactoside alpha-2,3-sialyltransferase 4 | -0.86 | 3.06E-05 | 2.61E-03 |
| RASSF1 | Ras association domain family member 1 | -0.85 | 1.04E-06 | 1.79E-04 |
| SLC1A7 | solute carrier family 1 member 7 | -0.85 | 5.24E-09 | 2.41E-06 |
| PDLIM1 | PDZ and LIM domain 1 | -0.85 | 1.71E-04 | 8.97E-03 |
| LTBP4 | latent transforming growth factor beta binding protein 4 | -0.85 | 3.59E-05 | 2.86E-03 |
| SCD5 | stearoyl-CoA desaturase 5 | -0.85 | 8.19E-06 | 9.36E-04 |
| ATXN1 | ataxin 1 | -0.84 | 5.53E-06 | 6.93E-04 |
| CRIP1 | cysteine rich protein 1 | -0.84 | 5.12E-04 | 1.99E-02 |
| KRT17P8 | keratin 17 pseudogene 8 | -0.84 | 7.81E-04 | 2.68E-02 |
| YPEL1 | yippee like 1 | -0.83 | 2.42E-06 | 3.66E-04 |
| CRIM1 | cysteine rich transmembrane BMP regulator 1 | -0.83 | 5.57E-06 | 6.93E-04 |
| GBP5 | guanylate binding protein 5 | -0.83 | 8.98E-06 | 9.97E-04 |
| TOX | thymocyte selection associated high mobility group box | -0.83 | 4.97E-06 | 6.49E-04 |
| KLRA1P | killer cell lectin like receptor A1, pseudogene | -0.83 | 6.78E-04 | 2.44E-02 |
| RNF166 | ring finger protein 166 | -0.83 | 3.18E-05 | 2.68E-03 |
| AC008750.1 |  | -0.82 | 1.32E-04 | 7.39E-03 |
| MXRA7 | matrix remodeling associated 7 | -0.82 | 6.81E-06 | 8.03E-04 |
| MXD4 | MAX dimerization protein 4 | -0.81 | 4.59E-05 | 3.44E-03 |
| BBC3 | BCL2 binding component 3 | -0.81 | 1.52E-03 | 4.23E-02 |
| TNFSF9 | TNF superfamily member 9 | -0.81 | 4.40E-04 | 1.80E-02 |
| AC044797.1 | Occludin pseudogene | -0.81 | 2.89E-04 | 1.30E-02 |
| AGPAT4 | 1-acylglycerol-3-phosphate O-acyltransferase 4 | -0.81 | 6.02E-05 | 4.26E-03 |
| NPC1 | NPC intracellular cholesterol transporter 1 | -0.81 | 2.34E-05 | 2.17E-03 |
| AC092139.3 |  | -0.80 | 1.99E-04 | 1.00E-02 |
| ZFYVE28 | zinc finger FYVE-type containing 28 | -0.80 | 1.06E-05 | 1.14E-03 |
| ZNF683 | zinc finger protein 683 | -0.80 | 7.49E-05 | 4.89E-03 |
| AL078645.1 |  | -0.80 | 1.94E-05 | 1.86E-03 |
| CTD-2377D24.8 |  | -0.80 | 2.96E-04 | 1.33E-02 |
| FCGR2B | Fc fragment of IgG receptor IIb | -0.80 | 5.11E-04 | 1.99E-02 |
| NECTIN1 | nectin cell adhesion molecule 1 | -0.80 | 7.20E-04 | 2.54E-02 |
| CD81 | CD81 molecule | -0.80 | 2.35E-05 | 2.17E-03 |
| HNRNPLL | heterogeneous nuclear ribonucleoprotein L like | -0.80 | 2.00E-06 | 3.16E-04 |
| ACTN4 | actinin alpha 4 | -0.79 | 1.13E-05 | 1.18E-03 |
| UAP1 | UDP-N-acetylglucosamine pyrophosphorylase 1 | -0.79 | 1.06E-04 | 6.35E-03 |
| F2R | coagulation factor II thrombin receptor | -0.79 | 9.24E-07 | 1.64E-04 |
| MYO1F | myosin IF | -0.79 | 1.92E-05 | 1.85E-03 |
| MPST | mercaptopyruvate sulfurtransferase | -0.79 | 3.52E-04 | 1.51E-02 |
| ST7 | suppression of tumorigenicity 7 | -0.79 | 6.45E-05 | 4.46E-03 |
| MYO6 | myosin VI | -0.79 | 1.63E-05 | 1.62E-03 |
| AOAH | acyloxyacyl hydrolase | -0.79 | 5.83E-04 | 2.18E-02 |
| ATP1A3 | ATPase Na+/K+ transporting subunit alpha 3 | -0.79 | 6.89E-04 | 2.46E-02 |
| FOXP4 | forkhead box P4 | -0.79 | 2.64E-04 | 1.23E-02 |
| DLEU1 | deleted in lymphocytic leukemia 1 (non-protein coding) | -0.78 | 6.65E-05 | 4.52E-03 |
| IFNLR1 | interferon lambda receptor 1 | -0.78 | 1.51E-04 | 8.20E-03 |
| LINC02084 | long intergenic non-protein coding RNA 2084 | -0.78 | 9.21E-05 | 5.66E-03 |
| SUSD1 | sushi domain containing 1 | -0.78 | 8.42E-06 | 9.48E-04 |
| ZNF487 | zinc finger protein 487 | -0.78 | 1.14E-04 | 6.56E-03 |
| XPNPEP2 | X-prolyl aminopeptidase 2 | -0.78 | 5.33E-06 | 6.74E-04 |
| CERCAM | cerebral endothelial cell adhesion molecule | -0.77 | 2.76E-05 | 2.43E-03 |
| AMOT | angiomotin | -0.77 | 3.45E-05 | 2.79E-03 |
| PLEKHG3 | pleckstrin homology and RhoGEF domain containing G3 | -0.77 | 1.02E-06 | 1.79E-04 |
| JAKMIP1 | janus kinase and microtubule interacting protein 1 | -0.77 | 6.27E-05 | 4.40E-03 |
| OSBPL5 | oxysterol binding protein like 5 | -0.77 | 8.87E-05 | 5.52E-03 |
| PTMS | parathymosin | -0.77 | 7.38E-05 | 4.89E-03 |
| ATP2B4 | ATPase plasma membrane Ca2+ transporting 4 | -0.77 | 3.18E-05 | 2.68E-03 |
| RABGAP1L | RAB GTPase activating protein 1 like | -0.77 | 2.10E-06 | 3.24E-04 |
| PPP1R16B | protein phosphatase 1 regulatory subunit 16B | -0.77 | 9.74E-05 | 5.92E-03 |
| SLC20A1 | solute carrier family 20 member 1 | -0.77 | 3.82E-07 | 8.08E-05 |
| MVD | mevalonate diphosphate decarboxylase | -0.77 | 8.72E-04 | 2.85E-02 |
| AC073957.3 |  | -0.77 | 5.26E-04 | 2.04E-02 |
| KCNQ1OT1 | KCNQ1 opposite strand/antisense transcript 1 (non-protein coding) | -0.77 | 6.76E-04 | 2.44E-02 |
| BPGM | bisphosphoglycerate mutase | -0.76 | 1.36E-04 | 7.56E-03 |
| SYT11 | synaptotagmin 11 | -0.76 | 6.81E-06 | 8.03E-04 |
| NATD1 | N-acetyltransferase domain containing 1 | -0.76 | 2.12E-04 | 1.05E-02 |
| C4orf50 | chromosome 4 open reading frame 50 | -0.76 | 2.61E-05 | 2.35E-03 |
| WEE1 | WEE1 G2 checkpoint kinase | -0.76 | 6.53E-05 | 4.48E-03 |
| FBXW4P1 | F-box and WD repeat domain containing 4 pseudogene 1 | -0.76 | 1.88E-03 | 4.96E-02 |
| ITGAM | integrin subunit alpha M | -0.75 | 4.04E-06 | 5.65E-04 |
| OSBPL7 | oxysterol binding protein like 7 | -0.75 | 6.47E-05 | 4.46E-03 |
| SNX18P13 | sorting nexin 18 pseudogene 13 | -0.75 | 4.80E-04 | 1.91E-02 |
| ABI3 | ABI family member 3 | -0.75 | 3.11E-04 | 1.37E-02 |
| MIAT | myocardial infarction associated transcript (non-protein coding) | -0.75 | 3.91E-05 | 3.00E-03 |
| AGAP1 | ArfGAP with GTPase domain, ankyrin repeat and PH domain 1 | -0.75 | 3.40E-05 | 2.77E-03 |
| ZNF821 | zinc finger protein 821 | -0.75 | 2.89E-04 | 1.30E-02 |
| FUT11 | fucosyltransferase 11 | -0.75 | 8.76E-05 | 5.47E-03 |
| SH3RF2 | SH3 domain containing ring finger 2 | -0.75 | 3.32E-05 | 2.74E-03 |
| AC004865.2 | uncharacterized LOC105378645 | -0.75 | 4.61E-04 | 1.86E-02 |
| LINC00869 | long intergenic non-protein coding RNA 869 | -0.75 | 1.14E-03 | 3.44E-02 |
| GK5 | glycerol kinase 5 (putative) | -0.75 | 3.44E-05 | 2.79E-03 |
| ENC1 | ectodermal-neural cortex 1 | -0.75 | 2.15E-05 | 2.01E-03 |
| ARHGAP33 | Rho GTPase activating protein 33 | -0.75 | 8.22E-04 | 2.75E-02 |
| RAPGEF1 | Rap guanine nucleotide exchange factor 1 | -0.75 | 4.62E-05 | 3.44E-03 |
| SERTAD1 | SERTA domain containing 1 | -0.75 | 4.58E-04 | 1.86E-02 |
| TGFB1 | transforming growth factor beta 1 | -0.74 | 1.11E-04 | 6.56E-03 |
| RP11-485G4.2 |  | -0.74 | 3.76E-05 | 2.92E-03 |
| AL928654.3 |  | -0.74 | 1.28E-04 | 7.25E-03 |
| BHLHE40 | basic helix-loop-helix family member e40 | -0.74 | 2.32E-04 | 1.11E-02 |
| MAPKAPK2 | mitogen-activated protein kinase-activated protein kinase 2 | -0.74 | 3.84E-05 | 2.97E-03 |
| MVB12B | multivesicular body subunit 12B | -0.73 | 1.23E-03 | 3.64E-02 |
| PTPRJ | protein tyrosine phosphatase, receptor type J | -0.73 | 3.06E-05 | 2.61E-03 |
| DGKQ | diacylglycerol kinase theta | -0.73 | 2.99E-04 | 1.33E-02 |
| DHCR24 | 24-dehydrocholesterol reductase | -0.73 | 2.69E-04 | 1.24E-02 |
| MYO3B | myosin IIIB | -0.73 | 2.20E-08 | 7.55E-06 |
| DUSP8 | dual specificity phosphatase 8 | -0.72 | 1.41E-03 | 4.02E-02 |
| SGCE | sarcoglycan epsilon | -0.72 | 2.58E-04 | 1.21E-02 |
| ARAP2 | ArfGAP with RhoGAP domain, ankyrin repeat and PH domain 2 | -0.72 | 5.79E-05 | 4.15E-03 |
| FLNA | filamin A | -0.72 | 2.99E-04 | 1.33E-02 |
| STOM | stomatin | -0.72 | 7.60E-05 | 4.89E-03 |
| SYNGR3 | synaptogyrin 3 | -0.72 | 3.55E-04 | 1.52E-02 |
| AL078645.2 | TRNA methyltransferase 2 homolog B (S. Cerevisiae) pseudogene | -0.72 | 2.82E-04 | 1.28E-02 |
| FYN | FYN proto-oncogene, Src family tyrosine kinase | -0.72 | 4.77E-06 | 6.35E-04 |
| SLC35G2 | solute carrier family 35 member G2 | -0.71 | 6.45E-05 | 4.46E-03 |
| NUMBL | NUMB like, endocytic adaptor protein | -0.71 | 5.58E-04 | 2.12E-02 |
| NOP14-AS1 | NOP14 antisense RNA 1 | -0.71 | 1.37E-04 | 7.60E-03 |
| MSC-AS1 | MSC antisense RNA 1 | -0.71 | 2.10E-05 | 1.99E-03 |
| CXCR1 | C-X-C motif chemokine receptor 1 | -0.71 | 1.21E-04 | 6.96E-03 |
| C12orf75 | chromosome 12 open reading frame 75 | -0.71 | 1.13E-04 | 6.56E-03 |
| LILRB1 | leukocyte immunoglobulin like receptor B1 | -0.71 | 6.18E-06 | 7.45E-04 |
| AC008750.2 | uncharacterized LOC100129083 | -0.71 | 3.90E-04 | 1.64E-02 |
| ITGB2 | integrin subunit beta 2 | -0.71 | 1.10E-05 | 1.17E-03 |
| GNG2 | G protein subunit gamma 2 | -0.71 | 4.28E-07 | 8.59E-05 |
| MSC | musculin | -0.71 | 1.55E-04 | 8.38E-03 |
| FAM129A | family with sequence similarity 129 member A | -0.71 | 1.17E-05 | 1.21E-03 |
| ARHGAP10 | Rho GTPase activating protein 10 | -0.71 | 6.96E-05 | 4.69E-03 |
| TRPC3 | transient receptor potential cation channel subfamily C member 3 | -0.70 | 6.13E-04 | 2.27E-02 |
| EOMES | eomesodermin | -0.70 | 3.30E-04 | 1.44E-02 |
| DDR2 | discoidin domain receptor tyrosine kinase 2 | -0.70 | 5.33E-04 | 2.05E-02 |
| DAPK2 | death associated protein kinase 2 | -0.70 | 6.14E-06 | 7.45E-04 |
| DOK2 | docking protein 2 | -0.70 | 2.09E-04 | 1.04E-02 |
| PDGFRB | platelet derived growth factor receptor beta | -0.70 | 2.49E-04 | 1.17E-02 |
| ACTA2 | actin, alpha 2, smooth muscle, aorta | -0.70 | 2.77E-05 | 2.43E-03 |
| ARNTL | aryl hydrocarbon receptor nuclear translocator like | -0.70 | 7.62E-06 | 8.82E-04 |
| MRPL10 | mitochondrial ribosomal protein L10 | -0.70 | 4.47E-04 | 1.82E-02 |
| IDS | iduronate 2-sulfatase | -0.70 | 3.59E-05 | 2.86E-03 |
| IL7 | interleukin 7 | -0.70 | 7.39E-05 | 4.89E-03 |
| SNORD17 | small nucleolar RNA, C/D box 17 | -0.70 | 1.83E-03 | 4.86E-02 |
| TFDP2 | transcription factor Dp-2 | -0.69 | 2.04E-04 | 1.02E-02 |
| PLEKHG1 | pleckstrin homology and RhoGEF domain containing G1 | -0.69 | 2.85E-05 | 2.47E-03 |
| GPR137B | G protein-coupled receptor 137B | -0.69 | 7.88E-04 | 2.68E-02 |
| GPR68 | G protein-coupled receptor 68 | -0.69 | 1.87E-03 | 4.93E-02 |
| DNMBP | dynamin binding protein | -0.69 | 2.86E-04 | 1.29E-02 |
| B4GALT5 | beta-1,4-galactosyltransferase 5 | -0.69 | 3.70E-05 | 2.91E-03 |
| ENPP4 | ectonucleotide pyrophosphatase/phosphodiesterase 4 | -0.68 | 1.70E-04 | 8.92E-03 |
| MYO1G | myosin IG | -0.68 | 1.98E-04 | 1.00E-02 |
| TRPS1 | transcriptional repressor GATA binding 1 | -0.68 | 1.59E-03 | 4.41E-02 |
| SERTAD3 | SERTA domain containing 3 | -0.68 | 2.40E-04 | 1.13E-02 |
| FCRLB | Fc receptor like B | -0.68 | 5.40E-04 | 2.07E-02 |
| AC008750.7 |  | -0.68 | 1.70E-03 | 4.58E-02 |
| DNAJC1 | DnaJ heat shock protein family (Hsp40) member C1 | -0.68 | 2.44E-04 | 1.15E-02 |
| TMX4 | thioredoxin related transmembrane protein 4 | -0.68 | 6.48E-05 | 4.46E-03 |
| SPON2 | spondin 2 | -0.68 | 1.72E-04 | 8.97E-03 |
| APMAP | adipocyte plasma membrane associated protein | -0.68 | 1.87E-04 | 9.58E-03 |
| ARPC5L | actin related protein 2/3 complex subunit 5 like | -0.67 | 4.60E-04 | 1.86E-02 |
| DDN-AS1 | DDN and PRKAG1 antisense RNA 1 | -0.67 | 3.33E-04 | 1.45E-02 |
| PRR5L | proline rich 5 like | -0.67 | 4.42E-05 | 3.35E-03 |
| PYHIN1 | pyrin and HIN domain family member 1 | -0.67 | 2.12E-04 | 1.05E-02 |
| EOGT | EGF domain specific O-linked N-acetylglucosamine transferase | -0.67 | 7.66E-05 | 4.91E-03 |
| SIPA1 | signal-induced proliferation-associated 1 | -0.67 | 1.51E-03 | 4.23E-02 |
| AC093616.1 | anaphase-promoting complex subunit 1-like | -0.67 | 1.18E-06 | 1.95E-04 |
| MIR4435-2HG | MIR4435-2 host gene | -0.66 | 7.46E-05 | 4.89E-03 |
| TSEN54 | tRNA splicing endonuclease subunit 54 | -0.66 | 1.50E-04 | 8.19E-03 |
| PPP2R5C | protein phosphatase 2 regulatory subunit B'gamma | -0.66 | 3.02E-05 | 2.60E-03 |
| CTSW | cathepsin W | -0.66 | 1.42E-04 | 7.85E-03 |
| KIF13B | kinesin family member 13B | -0.66 | 9.50E-05 | 5.82E-03 |
| DUSP2 | dual specificity phosphatase 2 | -0.66 | 1.05E-03 | 3.24E-02 |
| CDC14B | cell division cycle 14B | -0.66 | 9.50E-04 | 3.03E-02 |
| PXN | paxillin | -0.66 | 1.13E-04 | 6.56E-03 |
| ABCA2 | ATP binding cassette subfamily A member 2 | -0.66 | 1.03E-03 | 3.23E-02 |
| ARHGAP21 | Rho GTPase activating protein 21 | -0.65 | 1.58E-04 | 8.48E-03 |
| FBXL6 | F-box and leucine rich repeat protein 6 | -0.65 | 1.04E-03 | 3.24E-02 |
| SSBP3 | single stranded DNA binding protein 3 | -0.65 | 5.28E-04 | 2.04E-02 |
| UBXN10 | UBX domain protein 10 | -0.65 | 2.40E-05 | 2.20E-03 |
| BATF | basic leucine zipper ATF-like transcription factor | -0.65 | 1.40E-03 | 4.02E-02 |
| AC007384.1 | uncharacterized LOC101927902 | -0.65 | 1.40E-03 | 4.02E-02 |
| TNFRSF1B | TNF receptor superfamily member 1B | -0.65 | 3.10E-04 | 1.37E-02 |
| USP28 | ubiquitin specific peptidase 28 | -0.65 | 4.84E-04 | 1.92E-02 |
| AGO4 | argonaute 4, RISC catalytic component | -0.64 | 8.51E-04 | 2.80E-02 |
| PEG10 | paternally expressed 10 | -0.64 | 1.28E-03 | 3.74E-02 |
| RF00019 |  | -0.64 | 1.26E-03 | 3.72E-02 |
| SLC2A1 | solute carrier family 2 member 1 | -0.64 | 9.62E-04 | 3.06E-02 |
| ZBTB38 | zinc finger and BTB domain containing 38 | -0.64 | 8.24E-04 | 2.75E-02 |
| MCOLN2 | mucolipin 2 | -0.64 | 1.11E-03 | 3.38E-02 |
| TBC1D19 | TBC1 domain family member 19 | -0.64 | 5.95E-04 | 2.22E-02 |
| GOLM1 | golgi membrane protein 1 | -0.64 | 9.43E-04 | 3.01E-02 |
| HOXC4 | homeobox C4 | -0.64 | 1.04E-03 | 3.24E-02 |
| ZDHHC14 | zinc finger DHHC-type containing 14 | -0.63 | 1.68E-03 | 4.57E-02 |
| GZMA | granzyme A | -0.63 | 1.13E-04 | 6.56E-03 |
| LAX1 | lymphocyte transmembrane adaptor 1 | -0.63 | 4.95E-04 | 1.95E-02 |
| GTDC1 | glycosyltransferase like domain containing 1 | -0.63 | 1.42E-03 | 4.05E-02 |
| CHST7 | carbohydrate sulfotransferase 7 | -0.63 | 1.27E-03 | 3.74E-02 |
| FAS | Fas cell surface death receptor | -0.63 | 2.40E-04 | 1.13E-02 |
| PRKCH | protein kinase C eta | -0.63 | 1.07E-04 | 6.37E-03 |
| NHSL2 | NHS like 2 | -0.63 | 1.02E-04 | 6.17E-03 |
| IFNG | interferon gamma | -0.63 | 6.86E-04 | 2.45E-02 |
| IFFO2 | intermediate filament family orphan 2 | -0.63 | 1.20E-03 | 3.58E-02 |
| EIF5A2 | eukaryotic translation initiation factor 5A2 | -0.63 | 2.37E-04 | 1.13E-02 |
| AHNAK | AHNAK nucleoprotein | -0.63 | 1.24E-04 | 7.04E-03 |
| FHAD1 | forkhead associated phosphopeptide binding domain 1 | -0.62 | 3.72E-05 | 2.92E-03 |
| PAFAH2 | platelet activating factor acetylhydrolase 2 | -0.62 | 8.07E-04 | 2.72E-02 |
| ASCL2 | achaete-scute family bHLH transcription factor 2 | -0.62 | 1.61E-03 | 4.44E-02 |
| ABCA3 | ATP binding cassette subfamily A member 3 | -0.62 | 7.11E-04 | 2.51E-02 |
| SPOPL | speckle type BTB/POZ protein like | -0.62 | 5.61E-05 | 4.05E-03 |
| SPATA20 | spermatogenesis associated 20 | -0.62 | 1.74E-03 | 4.67E-02 |
| NAA50 | N(alpha)-acetyltransferase 50, NatE catalytic subunit | -0.62 | 1.35E-04 | 7.55E-03 |
| RDH10 | retinol dehydrogenase 10 | -0.62 | 1.62E-03 | 4.45E-02 |
| ERBB2 | erb-b2 receptor tyrosine kinase 2 | -0.62 | 3.70E-04 | 1.57E-02 |
| AL138756.1 |  | -0.62 | 9.34E-04 | 2.99E-02 |
| MYBL1 | MYB proto-oncogene like 1 | -0.61 | 4.27E-04 | 1.77E-02 |
| BX322639.1 | zinc finger protein 91 pseudogene | -0.61 | 7.96E-05 | 5.08E-03 |
| RGS3 | regulator of G protein signaling 3 | -0.61 | 1.61E-03 | 4.44E-02 |
| CDCA4 | cell division cycle associated 4 | -0.61 | 1.35E-03 | 3.94E-02 |
| LINC02432 | long intergenic non-protein coding RNA 2432 | -0.61 | 1.86E-03 | 4.92E-02 |
| CD8A | CD8a molecule | -0.61 | 7.65E-04 | 2.64E-02 |
| DLG3 | discs large MAGUK scaffold protein 3 | -0.61 | 4.34E-04 | 1.79E-02 |
| ARHGEF12 | Rho guanine nucleotide exchange factor 12 | -0.61 | 7.58E-05 | 4.89E-03 |
| RAB37 | RAB37, member RAS oncogene family | -0.61 | 9.77E-04 | 3.10E-02 |
| FAM160B1 | family with sequence similarity 160 member B1 | -0.61 | 2.25E-04 | 1.09E-02 |
| SYTL2 | synaptotagmin like 2 | -0.60 | 7.08E-04 | 2.51E-02 |
| PADI6 | peptidyl arginine deiminase 6 | -0.60 | 1.51E-03 | 4.22E-02 |
| TP53I11 | tumor protein p53 inducible protein 11 | -0.60 | 3.68E-05 | 2.91E-03 |
| PCBP4 | poly(rC) binding protein 4 | -0.60 | 1.40E-03 | 4.02E-02 |
| SRPK2 | SRSF protein kinase 2 | -0.60 | 4.25E-05 | 3.24E-03 |
| C1orf216 | chromosome 1 open reading frame 216 | -0.60 | 9.75E-04 | 3.10E-02 |
| ARL8A | ADP ribosylation factor like GTPase 8A | -0.60 | 6.76E-04 | 2.44E-02 |
| NFATC2 | nuclear factor of activated T cells 2 | -0.60 | 2.77E-04 | 1.27E-02 |
| SELENOS | selenoprotein S | -0.60 | 5.44E-04 | 2.08E-02 |
| LGR6 | leucine rich repeat containing G protein-coupled receptor 6 | -0.60 | 4.32E-04 | 1.79E-02 |
| RAP2B | RAP2B, member of RAS oncogene family | -0.60 | 1.57E-03 | 4.36E-02 |
| ARHGEF28 | Rho guanine nucleotide exchange factor 28 | -0.59 | 1.13E-05 | 1.18E-03 |
| KDM4B | lysine demethylase 4B | -0.59 | 1.86E-03 | 4.92E-02 |
| TSPAN2 | tetraspanin 2 | -0.59 | 2.02E-04 | 1.01E-02 |
| MAP3K5 | mitogen-activated protein kinase kinase kinase 5 | -0.59 | 5.36E-04 | 2.06E-02 |
| GALNT10 | polypeptide N-acetylgalactosaminyltransferase 10 | -0.59 | 1.66E-04 | 8.78E-03 |
| GNGT2 | G protein subunit gamma transducin 2 | -0.58 | 1.79E-03 | 4.78E-02 |
| EIF4G3 | eukaryotic translation initiation factor 4 gamma 3 | -0.58 | 1.37E-03 | 3.98E-02 |
| SYNE2 | spectrin repeat containing nuclear envelope protein 2 | -0.58 | 5.83E-05 | 4.16E-03 |
| SAP30 | Sin3A associated protein 30 | -0.58 | 5.80E-04 | 2.18E-02 |
| RHOT1P2 | ras homolog family member T1 pseudogene 2 | -0.58 | 9.97E-04 | 3.13E-02 |
| STARD4 | StAR related lipid transfer domain containing 4 | -0.58 | 1.63E-04 | 8.66E-03 |
| CTNNA1 | catenin alpha 1 | -0.58 | 1.26E-03 | 3.73E-02 |
| PRR29 | proline rich 29 | -0.57 | 8.39E-04 | 2.78E-02 |
| ITPRIPL1 | ITPRIP like 1 | -0.57 | 4.93E-04 | 1.95E-02 |
| KLRD1 | killer cell lectin like receptor D1 | -0.57 | 8.36E-06 | 9.48E-04 |
| CFLAR | CASP8 and FADD like apoptosis regulator | -0.56 | 1.22E-03 | 3.63E-02 |
| SMAD3 | SMAD family member 3 | -0.56 | 1.83E-03 | 4.87E-02 |
| DPY19L1P1 | DPY19L1 pseudogene 1 | -0.56 | 2.44E-06 | 3.66E-04 |
| PHF21A | PHD finger protein 21A | -0.55 | 2.97E-04 | 1.33E-02 |
| INSIG1 | insulin induced gene 1 | -0.55 | 1.41E-03 | 4.02E-02 |
| PLEKHA2 | pleckstrin homology domain containing A2 | -0.55 | 2.78E-04 | 1.27E-02 |
| SIGIRR | single Ig and TIR domain containing | -0.55 | 1.53E-03 | 4.26E-02 |
| ADIPOR2 | adiponectin receptor 2 | -0.55 | 1.04E-03 | 3.24E-02 |
| RFTN1 | raftlin, lipid raft linker 1 | -0.55 | 2.77E-04 | 1.27E-02 |
| AC009495.3 |  | -0.55 | 4.83E-04 | 1.92E-02 |
| FAM102B | family with sequence similarity 102 member B | -0.55 | 1.72E-03 | 4.62E-02 |
| WDR47 | WD repeat domain 47 | -0.54 | 1.14E-03 | 3.44E-02 |
| ZNF335 | zinc finger protein 335 | -0.54 | 1.38E-03 | 3.98E-02 |
| SIRT2 | sirtuin 2 | -0.54 | 1.09E-03 | 3.34E-02 |
| CPD | carboxypeptidase D | -0.54 | 8.16E-04 | 2.74E-02 |
| PDZD4 | PDZ domain containing 4 | -0.54 | 4.37E-04 | 1.79E-02 |
| AC093567.1 | uncharacterized LOC105372105 | -0.53 | 2.65E-04 | 1.23E-02 |
| FTH1P22 | ferritin heavy chain 1 pseudogene 22 | -0.53 | 2.18E-07 | 4.88E-05 |
| PROK2 | prokineticin 2 | -0.53 | 3.82E-07 | 8.08E-05 |
| AUTS2 | AUTS2, activator of transcription and developmental regulator | -0.53 | 8.76E-04 | 2.85E-02 |
| LEXM | lymphocyte expansion molecule | -0.53 | 1.68E-04 | 8.90E-03 |
| DLG5 | discs large MAGUK scaffold protein 5 | -0.53 | 4.05E-04 | 1.70E-02 |
| LINC02384 | long intergenic non-protein coding RNA 2384 | -0.53 | 1.05E-04 | 6.32E-03 |
| TRPV3 | transient receptor potential cation channel subfamily V member 3 | -0.53 | 1.47E-03 | 4.16E-02 |
| AC068491.4 |  | -0.52 | 9.82E-04 | 3.10E-02 |
| PIP4K2C | phosphatidylinositol-5-phosphate 4-kinase type 2 gamma | -0.52 | 1.49E-03 | 4.19E-02 |
| NDRG1 | N-myc downstream regulated 1 | -0.52 | 6.60E-04 | 2.41E-02 |
| NUAK1 | NUAK family kinase 1 | -0.51 | 4.55E-05 | 3.43E-03 |
| AC090152.1 | uncharacterized LOC100505501 | -0.51 | 5.70E-04 | 2.15E-02 |
| NMUR1 | neuromedin U receptor 1 | -0.51 | 8.33E-05 | 5.27E-03 |
| PTP4A2 | protein tyrosine phosphatase type IVA, member 2 | -0.51 | 7.45E-04 | 2.60E-02 |
| RALGDS | ral guanine nucleotide dissociation stimulator | -0.51 | 1.03E-03 | 3.23E-02 |
| HERPUD2 | HERPUD family member 2 | -0.50 | 7.10E-04 | 2.51E-02 |
| PQLC3 | PQ loop repeat containing 3 | -0.50 | 1.85E-03 | 4.89E-02 |
| SYNM | synemin | -0.50 | 2.08E-04 | 1.04E-02 |
| BFSP1 | beaded filament structural protein 1 | -0.50 | 2.67E-04 | 1.24E-02 |
| PLEK | pleckstrin | -0.50 | 9.22E-04 | 2.96E-02 |
| KIF21B | kinesin family member 21B | -0.49 | 7.80E-04 | 2.68E-02 |
| OPTN | optineurin | -0.49 | 9.94E-04 | 3.13E-02 |
| SLCO4C1 | solute carrier organic anion transporter family member 4C1 | -0.48 | 1.77E-04 | 9.19E-03 |
| RAB27B | RAB27B, member RAS oncogene family | -0.48 | 1.48E-03 | 4.18E-02 |
| CD244 | CD244 molecule | -0.47 | 3.14E-04 | 1.38E-02 |
| FADS1 | fatty acid desaturase 1 | -0.45 | 4.69E-04 | 1.88E-02 |
| LINC00944 | long intergenic non-protein coding RNA 944 | -0.42 | 1.60E-03 | 4.42E-02 |
| CD160 | CD160 molecule | -0.40 | 1.90E-03 | 4.99E-02 |
| NGFR | nerve growth factor receptor | -0.37 | 1.69E-03 | 4.58E-02 |
| CMKLR1 | chemerin chemokine-like receptor 1 | -0.35 | 8.51E-04 | 2.80E-02 |
| PALLD | palladin, cytoskeletal associated protein | -0.31 | 1.50E-03 | 4.20E-02 |
| ANKRD20A11P | ankyrin repeat domain 20 family member A11, pseudogene | -0.31 | 9.10E-04 | 2.93E-02 |
| CYP4F29P | cytochrome P450 family 4 subfamily F member 29, pseudogene | -0.30 | 1.19E-03 | 3.57E-02 |
| AL158071.3 |  | -0.30 | 5.04E-04 | 1.98E-02 |
| CDHR1 | cadherin related family member 1 | -0.30 | 1.64E-03 | 4.48E-02 |
| LINC00299 | long intergenic non-protein coding RNA 299 | -0.29 | 5.27E-04 | 2.04E-02 |
| GPR27 | G protein-coupled receptor 27 | -0.29 | 1.12E-03 | 3.41E-02 |
| AL158071.2 | uncharacterized LOC100507103 | -0.26 | 6.60E-04 | 2.41E-02 |
| ATP9A | ATPase phospholipid transporting 9A (putative) | -0.22 | 5.92E-04 | 2.21E-02 |
| HCG4P5 | HLA complex group 4 pseudogene 5 | -0.21 | 8.01E-04 | 2.72E-02 |
| EFNA5 | ephrin A5 | -0.20 | 9.80E-04 | 3.10E-02 |
| GNLY | granulysin | -0.20 | 8.39E-05 | 5.28E-03 |
| SATB2 | SATB homeobox 2 | -0.18 | 6.57E-04 | 2.41E-02 |
| IL5RA | interleukin 5 receptor subunit alpha | -0.11 | 1.12E-04 | 6.56E-03 |
| LINC00355 | long intergenic non-protein coding RNA 355 | -0.11 | 1.82E-06 | 2.90E-04 |
| GFPT2 | glutamine-fructose-6-phosphate transaminase 2 | -0.04 | 1.82E-03 | 4.84E-02 |
| GSTM1 | glutathione S-transferase mu 1 | 0.04 | 4.23E-06 | 5.73E-04 |
| PSPHP1 | phosphoserine phosphatase pseudogene 1 | 0.07 | 8.34E-07 | 1.53E-04 |
| RANBP17 | RAN binding protein 17 | 0.21 | 1.17E-03 | 3.52E-02 |
| RP11-65I12.1 |  | 0.27 | 8.76E-04 | 2.85E-02 |
| C21orf33 | chromosome 21 open reading frame 33 | 0.28 | 6.71E-04 | 2.44E-02 |
| IGKV4-1 | immunoglobulin kappa variable 4-1 | 0.29 | 1.19E-03 | 3.56E-02 |
| IGHV3-23 | immunoglobulin heavy variable 3-23 | 0.30 | 8.20E-04 | 2.74E-02 |
| IGLC3 | immunoglobulin lambda constant 3 (Kern-Oz+ marker) | 0.30 | 5.98E-04 | 2.22E-02 |
| PAX8-AS1 | PAX8 antisense RNA 1 | 0.41 | 7.43E-05 | 4.89E-03 |
| IGKV1-5 | immunoglobulin kappa variable 1-5 | 0.45 | 3.66E-04 | 1.55E-02 |
| IGHG1 | immunoglobulin heavy constant gamma 1 (G1m marker) | 0.45 | 3.16E-04 | 1.38E-02 |
| REG4 | regenerating family member 4 | 0.48 | 5.64E-04 | 2.13E-02 |
| AL031432.2 |  | 0.52 | 2.69E-05 | 2.41E-03 |
| FAM102A | family with sequence similarity 102 member A | 0.54 | 1.67E-03 | 4.55E-02 |
| DOCK9 | dedicator of cytokinesis 9 | 0.54 | 1.75E-03 | 4.70E-02 |
| RGS10 | regulator of G protein signaling 10 | 0.55 | 1.10E-03 | 3.37E-02 |
| IGLV3-19 | immunoglobulin lambda variable 3-19 | 0.56 | 1.91E-04 | 9.72E-03 |
| HSPG2 | heparan sulfate proteoglycan 2 | 0.56 | 2.19E-04 | 1.08E-02 |
| DEPDC7 | DEP domain containing 7 | 0.57 | 1.37E-03 | 3.98E-02 |
| IGHM | immunoglobulin heavy constant mu | 0.57 | 4.19E-04 | 1.75E-02 |
| TRAV39 | T cell receptor alpha variable 39 | 0.58 | 6.11E-04 | 2.27E-02 |
| MCF2L | MCF.2 cell line derived transforming sequence like | 0.58 | 1.44E-03 | 4.09E-02 |
| MFGE8 | milk fat globule-EGF factor 8 protein | 0.58 | 1.84E-03 | 4.87E-02 |
| ANKRD26 | ankyrin repeat domain 26 | 0.59 | 8.65E-04 | 2.83E-02 |
| GRAP | GRB2-related adaptor protein | 0.59 | 1.67E-03 | 4.55E-02 |
| AC124312.3 |  | 0.60 | 1.79E-04 | 9.25E-03 |
| PLA2G12A | phospholipase A2 group XIIA | 0.60 | 1.06E-03 | 3.26E-02 |
| PRKAR1B | protein kinase cAMP-dependent type I regulatory subunit beta | 0.60 | 1.17E-03 | 3.52E-02 |
| DMAC1 | distal membrane arm assembly complex 1 | 0.61 | 6.83E-04 | 2.45E-02 |
| AGPAT5 | 1-acylglycerol-3-phosphate O-acyltransferase 5 | 0.62 | 4.66E-04 | 1.87E-02 |
| UXS1 | UDP-glucuronate decarboxylase 1 | 0.62 | 1.49E-04 | 8.17E-03 |
| ADSL | adenylosuccinate lyase | 0.62 | 7.84E-04 | 2.68E-02 |
| ACTN1 | actinin alpha 1 | 0.62 | 1.20E-03 | 3.58E-02 |
| ORMDL3 | ORMDL sphingolipid biosynthesis regulator 3 | 0.62 | 4.77E-04 | 1.91E-02 |
| AP005131.6 |  | 0.62 | 3.45E-04 | 1.49E-02 |
| AC136475.3 |  | 0.62 | 1.12E-03 | 3.41E-02 |
| ZNF439 | zinc finger protein 439 | 0.63 | 7.66E-04 | 2.64E-02 |
| AKAP7 | A-kinase anchoring protein 7 | 0.63 | 1.76E-03 | 4.71E-02 |
| AC022916.1 | uncharacterized LOC105371932 | 0.63 | 1.66E-03 | 4.54E-02 |
| FAM117B | family with sequence similarity 117 member B | 0.63 | 5.79E-04 | 2.18E-02 |
| ITGA6 | integrin subunit alpha 6 | 0.63 | 1.81E-03 | 4.83E-02 |
| CUBN | cubilin | 0.64 | 8.30E-04 | 2.76E-02 |
| TMEM106B | transmembrane protein 106B | 0.64 | 1.24E-04 | 7.04E-03 |
| TPST1 | tyrosylprotein sulfotransferase 1 | 0.65 | 1.69E-03 | 4.58E-02 |
| ZNF629 | zinc finger protein 629 | 0.65 | 1.30E-03 | 3.79E-02 |
| CHML | CHM like, Rab escort protein 2 | 0.65 | 8.47E-04 | 2.80E-02 |
| DNMT3A | DNA methyltransferase 3 alpha | 0.65 | 5.09E-04 | 1.99E-02 |
| ZNF550 | zinc finger protein 550 | 0.65 | 7.83E-04 | 2.68E-02 |
| RNF157 | ring finger protein 157 | 0.66 | 1.30E-03 | 3.80E-02 |
| CERS6 | ceramide synthase 6 | 0.66 | 3.59E-04 | 1.54E-02 |
| RETREG1 | reticulophagy regulator 1 | 0.67 | 9.12E-04 | 2.93E-02 |
| SUSD3 | sushi domain containing 3 | 0.67 | 2.25E-04 | 1.09E-02 |
| MAN1C1 | mannosidase alpha class 1C member 1 | 0.67 | 8.11E-04 | 2.73E-02 |
| TSGA10 | testis specific 10 | 0.67 | 6.69E-04 | 2.43E-02 |
| MFHAS1 | malignant fibrous histiocytoma amplified sequence 1 | 0.68 | 8.84E-04 | 2.87E-02 |
| KDSR | 3-ketodihydrosphingosine reductase | 0.68 | 8.88E-04 | 2.87E-02 |
| PAICS | phosphoribosylaminoimidazole carboxylase and phosphoribosylaminoimidazolesuccinocarboxamide synthase | 0.68 | 8.52E-04 | 2.80E-02 |
| ACSL6 | acyl-CoA synthetase long chain family member 6 | 0.68 | 1.14E-03 | 3.44E-02 |
| CDCA7L | cell division cycle associated 7 like | 0.69 | 7.56E-04 | 2.63E-02 |
| ABLIM1 | actin binding LIM protein 1 | 0.69 | 2.31E-04 | 1.11E-02 |
| MYB | MYB proto-oncogene, transcription factor | 0.69 | 1.51E-03 | 4.23E-02 |
| CASP6 | caspase 6 | 0.69 | 6.31E-04 | 2.32E-02 |
| SPEG | SPEG complex locus | 0.69 | 7.01E-04 | 2.49E-02 |
| COL6A3 | collagen type VI alpha 3 chain | 0.69 | 8.41E-04 | 2.78E-02 |
| METAP1D | methionyl aminopeptidase type 1D, mitochondrial | 0.69 | 4.30E-04 | 1.78E-02 |
| KCNQ1 | potassium voltage-gated channel subfamily Q member 1 | 0.69 | 1.27E-03 | 3.73E-02 |
| ADTRP | androgen dependent TFPI regulating protein | 0.69 | 1.70E-03 | 4.58E-02 |
| USP6NL | USP6 N-terminal like | 0.69 | 6.86E-04 | 2.45E-02 |
| GRB10 | growth factor receptor bound protein 10 | 0.70 | 7.97E-04 | 2.71E-02 |
| AHCY | adenosylhomocysteinase | 0.70 | 7.42E-04 | 2.60E-02 |
| SLC7A6 | solute carrier family 7 member 6 | 0.70 | 1.69E-04 | 8.92E-03 |
| GLS2 | glutaminase 2 | 0.70 | 1.46E-03 | 4.15E-02 |
| HK2 | hexokinase 2 | 0.70 | 4.47E-04 | 1.82E-02 |
| GPR155 | G protein-coupled receptor 155 | 0.70 | 1.03E-03 | 3.23E-02 |
| GCSAM | germinal center associated signaling and motility | 0.70 | 1.45E-03 | 4.12E-02 |
| ZNF516 | zinc finger protein 516 | 0.71 | 6.59E-04 | 2.41E-02 |
| COX10-AS1 | COX10 antisense RNA 1 | 0.71 | 3.73E-04 | 1.57E-02 |
| DGKA | diacylglycerol kinase alpha | 0.71 | 5.88E-05 | 4.18E-03 |
| DPH5 | diphthamide biosynthesis 5 | 0.71 | 8.53E-05 | 5.35E-03 |
| PCYOX1L | prenylcysteine oxidase 1 like | 0.71 | 7.59E-05 | 4.89E-03 |
| AL034550.2 | uncharacterized LOC101929698 | 0.71 | 1.13E-04 | 6.56E-03 |
| EPHX2 | epoxide hydrolase 2 | 0.71 | 7.46E-04 | 2.60E-02 |
| STRBP | spermatid perinuclear RNA binding protein | 0.72 | 5.73E-06 | 7.07E-04 |
| PLAG1 | PLAG1 zinc finger | 0.72 | 2.22E-04 | 1.09E-02 |
| DHX32 | DEAH-box helicase 32 (putative) | 0.73 | 1.12E-03 | 3.41E-02 |
| PDE9A | phosphodiesterase 9A | 0.73 | 7.81E-04 | 2.68E-02 |
| NELL2 | neural EGFL like 2 | 0.74 | 5.10E-04 | 1.99E-02 |
| TTC9 | tetratricopeptide repeat domain 9 | 0.74 | 4.33E-04 | 1.79E-02 |
| LDHB | lactate dehydrogenase B | 0.74 | 2.25E-04 | 1.09E-02 |
| AC093157.1 | uncharacterized LOC102606465 | 0.74 | 1.39E-03 | 4.01E-02 |
| ZSWIM1 | zinc finger SWIM-type containing 1 | 0.74 | 7.60E-04 | 2.64E-02 |
| FAM153B | family with sequence similarity 153 member B | 0.74 | 2.27E-04 | 1.10E-02 |
| TNFRSF10D | TNF receptor superfamily member 10d | 0.75 | 5.53E-04 | 2.10E-02 |
| FBLN5 | fibulin 5 | 0.75 | 1.32E-03 | 3.83E-02 |
| STXBP1 | syntaxin binding protein 1 | 0.75 | 3.35E-04 | 1.45E-02 |
| AL162457.1 | uncharacterized LOC100128310 | 0.75 | 1.10E-03 | 3.37E-02 |
| FAM153A | family with sequence similarity 153 member A | 0.75 | 1.62E-04 | 8.66E-03 |
| IMPDH2 | inosine monophosphate dehydrogenase 2 | 0.75 | 5.00E-05 | 3.67E-03 |
| TCEA3 | transcription elongation factor A3 | 0.75 | 1.62E-03 | 4.45E-02 |
| CR2 | complement C3d receptor 2 | 0.76 | 7.53E-05 | 4.89E-03 |
| TRBV6-6 | T cell receptor beta variable 6-6 | 0.76 | 1.47E-03 | 4.16E-02 |
| CDH23 | cadherin related 23 | 0.76 | 3.53E-05 | 2.84E-03 |
| AQP3 | aquaporin 3 (Gill blood group) | 0.76 | 6.79E-04 | 2.44E-02 |
| AL357033.4 |  | 0.76 | 4.94E-04 | 1.95E-02 |
| LINC00476 | long intergenic non-protein coding RNA 476 | 0.76 | 1.49E-03 | 4.19E-02 |
| JAML | junction adhesion molecule like | 0.76 | 2.58E-04 | 1.21E-02 |
| CLN5 | CLN5, intracellular trafficking protein | 0.77 | 1.46E-04 | 8.07E-03 |
| USP51 | ubiquitin specific peptidase 51 | 0.78 | 1.07E-03 | 3.30E-02 |
| GP5 | glycoprotein V platelet | 0.79 | 2.99E-04 | 1.33E-02 |
| MINDY1 | MINDY lysine 48 deubiquitinase 1 | 0.79 | 5.50E-04 | 2.09E-02 |
| ACSM3 | acyl-CoA synthetase medium chain family member 3 | 0.79 | 1.98E-04 | 1.00E-02 |
| TBC1D4 | TBC1 domain family member 4 | 0.79 | 4.31E-05 | 3.28E-03 |
| SOX8 | SRY-box 8 | 0.79 | 8.91E-04 | 2.88E-02 |
| RIC3 | RIC3 acetylcholine receptor chaperone | 0.79 | 1.54E-04 | 8.32E-03 |
| LEF1 | lymphoid enhancer binding factor 1 | 0.79 | 6.64E-05 | 4.52E-03 |
| EFHD1 | EF-hand domain family member D1 | 0.80 | 3.67E-04 | 1.56E-02 |
| CSGALNACT1 | chondroitin sulfate N-acetylgalactosaminyltransferase 1 | 0.80 | 1.09E-03 | 3.35E-02 |
| TRAV23DV6 | T cell receptor alpha variable 23/delta variable 6 | 0.80 | 7.30E-04 | 2.57E-02 |
| DCDC1 | doublecortin domain containing 1 | 0.80 | 7.42E-04 | 2.60E-02 |
| SNHG8 | small nucleolar RNA host gene 8 | 0.80 | 1.68E-03 | 4.57E-02 |
| UBASH3B | ubiquitin associated and SH3 domain containing B | 0.80 | 2.72E-05 | 2.41E-03 |
| CD27 | CD27 molecule | 0.82 | 7.66E-06 | 8.82E-04 |
| PCSK5 | proprotein convertase subtilisin/kexin type 5 | 0.82 | 7.12E-05 | 4.78E-03 |
| AGMAT | agmatinase | 0.83 | 8.94E-05 | 5.54E-03 |
| DENND5A | DENN domain containing 5A | 0.83 | 1.82E-04 | 9.37E-03 |
| AC007342.8 |  | 0.83 | 8.78E-04 | 2.85E-02 |
| TXK | TXK tyrosine kinase | 0.83 | 2.05E-05 | 1.95E-03 |
| CNKSR2 | connector enhancer of kinase suppressor of Ras 2 | 0.84 | 1.81E-04 | 9.35E-03 |
| TMEM14C | transmembrane protein 14C | 0.84 | 3.42E-04 | 1.48E-02 |
| S100B | S100 calcium binding protein B | 0.84 | 6.20E-04 | 2.29E-02 |
| CASP10 | caspase 10 | 0.84 | 1.80E-05 | 1.75E-03 |
| FAM153C | family with sequence similarity 153 member C | 0.85 | 1.60E-04 | 8.58E-03 |
| PRKCQ-AS1 | PRKCQ antisense RNA 1 | 0.86 | 2.61E-06 | 3.84E-04 |
| TRABD2A | TraB domain containing 2A | 0.86 | 3.30E-05 | 2.74E-03 |
| KLHL6 | kelch like family member 6 | 0.86 | 9.51E-08 | 2.57E-05 |
| VIPR1 | vasoactive intestinal peptide receptor 1 | 0.87 | 1.11E-04 | 6.56E-03 |
| PDE6G | phosphodiesterase 6G | 0.87 | 1.30E-04 | 7.30E-03 |
| TRAV29DV5 | T cell receptor alpha variable 29/delta variable 5 (gene/pseudogene) | 0.88 | 4.62E-04 | 1.86E-02 |
| CHRM3-AS2 | CHRM3 antisense RNA 2 | 0.88 | 2.70E-05 | 2.41E-03 |
| SOCS2-AS1 | SOCS2 antisense RNA 1 | 0.88 | 2.24E-04 | 1.09E-02 |
| ALPK1 | alpha kinase 1 | 0.88 | 3.36E-05 | 2.76E-03 |
| SYNJ2 | synaptojanin 2 | 0.89 | 5.01E-06 | 6.49E-04 |
| RCAN3 | RCAN family member 3 | 0.89 | 3.75E-05 | 2.92E-03 |
| SLC5A5 | solute carrier family 5 member 5 | 0.89 | 1.84E-04 | 9.42E-03 |
| ARHGEF11 | Rho guanine nucleotide exchange factor 11 | 0.89 | 6.15E-05 | 4.33E-03 |
| AC007342.9 |  | 0.89 | 2.73E-04 | 1.26E-02 |
| STRADB | STE20-related kinase adaptor beta | 0.89 | 1.23E-04 | 7.04E-03 |
| CHMP7 | charged multivesicular body protein 7 | 0.89 | 1.35E-08 | 4.95E-06 |
| SERINC5 | serine incorporator 5 | 0.90 | 1.85E-05 | 1.79E-03 |
| RP11-28F1.2 |  | 0.91 | 5.09E-06 | 6.49E-04 |
| IGF1R | insulin like growth factor 1 receptor | 0.92 | 9.90E-06 | 1.08E-03 |
| TBC1D8B | TBC1 domain family member 8B | 0.92 | 9.70E-05 | 5.92E-03 |
| AC012636.1 | uncharacterized LOC101929215 | 0.92 | 4.77E-05 | 3.54E-03 |
| CAMSAP2 | calmodulin regulated spectrin associated protein family member 2 | 0.95 | 2.36E-05 | 2.17E-03 |
| EPHA1-AS1 | EPHA1 antisense RNA 1 | 0.96 | 6.64E-06 | 7.94E-04 |
| LAPTM4B | lysosomal protein transmembrane 4 beta | 0.96 | 7.36E-06 | 8.60E-04 |
| GABPB1-AS1 | GABPB1 antisense RNA 1 | 0.96 | 1.44E-05 | 1.46E-03 |
| LEF1-AS1 | LEF1 antisense RNA 1 | 0.97 | 1.11E-06 | 1.88E-04 |
| XKR9 | XK related 9 | 0.98 | 5.46E-05 | 3.99E-03 |
| SOCS2 | suppressor of cytokine signaling 2 | 0.99 | 6.93E-05 | 4.69E-03 |
| EPHA1 | EPH receptor A1 | 0.99 | 2.75E-06 | 3.97E-04 |
| DPP4 | dipeptidyl peptidase 4 | 0.99 | 5.62E-05 | 4.05E-03 |
| NPM3 | nucleophosmin/nucleoplasmin 3 | 1.01 | 8.59E-06 | 9.60E-04 |
| NLN | neurolysin | 1.01 | 4.30E-06 | 5.78E-04 |
| IL6R | interleukin 6 receptor | 1.01 | 8.02E-07 | 1.49E-04 |
| TRBV30 | T cell receptor beta variable 30 (gene/pseudogene) | 1.02 | 3.01E-04 | 1.33E-02 |
| IFNGR2 | interferon gamma receptor 2 | 1.02 | 9.19E-05 | 5.66E-03 |
| LINC00402 | long intergenic non-protein coding RNA 402 | 1.04 | 1.04E-05 | 1.13E-03 |
| STAP1 | signal transducing adaptor family member 1 | 1.04 | 1.17E-04 | 6.71E-03 |
| SLC40A1 | solute carrier family 40 member 1 | 1.05 | 2.82E-04 | 1.28E-02 |
| TMTC1 | transmembrane and tetratricopeptide repeat containing 1 | 1.06 | 7.58E-05 | 4.89E-03 |
| NRN1 | neuritin 1 | 1.08 | 5.07E-06 | 6.49E-04 |
| CA6 | carbonic anhydrase 6 | 1.10 | 2.12E-05 | 2.00E-03 |
| CEACAM1 | carcinoembryonic antigen related cell adhesion molecule 1 | 1.10 | 6.09E-06 | 7.45E-04 |
| ITM2C | integral membrane protein 2C | 1.11 | 4.07E-06 | 5.65E-04 |
| EFHC2 | EF-hand domain containing 2 | 1.11 | 1.65E-05 | 1.63E-03 |
| TRAV26-1 | T cell receptor alpha variable 26-1 | 1.14 | 1.63E-05 | 1.62E-03 |
| KLF13 | Kruppel like factor 13 | 1.15 | 6.39E-09 | 2.74E-06 |
| TMIGD2 | transmembrane and immunoglobulin domain containing 2 | 1.27 | 9.12E-10 | 5.41E-07 |
| RP3-477M7.5 |  | 1.28 | 1.86E-09 | 9.59E-07 |
| EIF2S3B | eukaryotic translation initiation factor 2 subunit gamma B | 1.28 | 1.27E-03 | 3.74E-02 |
| TRBV28 | T cell receptor beta variable 28 | 1.50 | 3.02E-10 | 2.33E-07 |

**Table S9**

Differentially expressed genes in CD8+ T cells of PM and DM patients excluding potential outliers. Genes with a positive FC are higher expressed in PM patients and genes with a negative FC are higher expressed in DM patients. P-values were estimated by the Wald test in DESeq2 adjusted for gender, age group, and RIN value. A FDR threshold of 5% based on the method of Benjamini-Hochberg was used to identify significant differentially expressed genes. Abbreviations: FC: Fold Change.

| **Gene symbol** | **Gene name** | **Log2FC** | **P-value** | **P-adjusted** |
| --- | --- | --- | --- | --- |
| AL365357.1 | ribosomal protein S14 pseudogene 2 | -3.37 | 5.02E-10 | 5.83E-07 |
| AL591846.1 | ribosomal protein S14 pseudogene 1 | -2.21 | 4.36E-09 | 3.46E-06 |
| IFI27 | interferon alpha inducible protein 27 | -2.03 | 9.42E-11 | 1.42E-07 |
| NLRP2 | NLR family pyrin domain containing 2 | -1.76 | 5.28E-06 | 1.23E-03 |
| FAM118A | family with sequence similarity 118 member A | -1.75 | 3.63E-09 | 3.04E-06 |
| HELZ2 | helicase with zinc finger 2 | -1.58 | 7.97E-15 | 6.73E-11 |
| PAX5 | paired box 5 | -1.49 | 7.32E-05 | 7.84E-03 |
| OASL | 2'-5'-oligoadenylate synthetase like | -1.46 | 1.17E-13 | 4.42E-10 |
| SH3BGRL2 | SH3 domain binding glutamate rich protein like 2 | -1.44 | 4.12E-07 | 1.59E-04 |
| TRAV2 | T cell receptor alpha variable 2 | -1.36 | 4.77E-05 | 5.90E-03 |
| EPSTI1 | epithelial stromal interaction 1 | -1.28 | 6.04E-12 | 1.52E-08 |
| TGFBR3 | transforming growth factor beta receptor 3 | -1.28 | 8.40E-14 | 4.23E-10 |
| ADGRG1 | adhesion G protein-coupled receptor G1 | -1.26 | 1.38E-10 | 1.89E-07 |
| NKG7 | natural killer cell granule protein 7 | -1.26 | 9.01E-11 | 1.42E-07 |
| ZEB2 | zinc finger E-box binding homeobox 2 | -1.22 | 1.30E-11 | 2.81E-08 |
| SH3TC1 | SH3 domain and tetratricopeptide repeats 1 | -1.20 | 2.05E-06 | 6.33E-04 |
| PRSS23 | serine protease 23 | -1.20 | 3.40E-11 | 6.42E-08 |
| ATP1A3 | ATPase Na+/K+ transporting subunit alpha 3 | -1.19 | 2.13E-08 | 1.46E-05 |
| CAPN15 | calpain 15 | -1.19 | 7.98E-08 | 4.30E-05 |
| EFHD2 | EF-hand domain family member D2 | -1.17 | 5.75E-10 | 6.20E-07 |
| MIDN | midnolin | -1.15 | 6.59E-06 | 1.40E-03 |
| PRKCQ-AS1 | PRKCQ antisense RNA 1 | -1.14 | 2.42E-08 | 1.59E-05 |
| MXD4 | MAX dimerization protein 4 | -1.14 | 6.44E-10 | 6.49E-07 |
| CASZ1 | castor zinc finger 1 | -1.13 | 4.65E-07 | 1.75E-04 |
| TRBV12-3 | T cell receptor beta variable 12-3 | -1.11 | 8.49E-04 | 4.34E-02 |
| SLC7A5P1 | solute carrier family 7 member 5 pseudogene 1 | -1.09 | 7.14E-05 | 7.77E-03 |
| LAG3 | lymphocyte activating 3 | -1.09 | 9.34E-08 | 4.55E-05 |
| FAM53B | family with sequence similarity 53 member B | -1.07 | 5.42E-08 | 3.03E-05 |
| SMAD7 | SMAD family member 7 | -1.06 | 4.82E-06 | 1.19E-03 |
| GZMH | granzyme H | -1.03 | 1.78E-08 | 1.28E-05 |
| AC040970.1 | uncharacterized LOC101927963 | -1.03 | 4.22E-06 | 1.08E-03 |
| ATG2A | autophagy related 2A | -1.02 | 2.96E-05 | 4.34E-03 |
| SLC2A1 | solute carrier family 2 member 1 | -1.01 | 3.09E-08 | 1.94E-05 |
| SETBP1 | SET binding protein 1 | -1.01 | 8.93E-08 | 4.49E-05 |
| OTOF | otoferlin | -1.01 | 7.56E-06 | 1.59E-03 |
| SLC16A6 | solute carrier family 16 member 6 | -0.99 | 8.60E-08 | 4.48E-05 |
| PDGFD | platelet derived growth factor D | -0.99 | 9.34E-06 | 1.83E-03 |
| B4GALT1 | beta-1,4-galactosyltransferase 1 | -0.99 | 1.23E-07 | 5.64E-05 |
| KIAA1671 | KIAA1671 | -0.99 | 8.22E-07 | 2.96E-04 |
| TBKBP1 | TBK1 binding protein 1 | -0.98 | 1.80E-05 | 3.05E-03 |
| AC009951.1 |  | -0.98 | 8.76E-05 | 8.87E-03 |
| SH2D2A | SH2 domain containing 2A | -0.96 | 2.31E-06 | 6.98E-04 |
| GNAO1 | G protein subunit alpha o1 | -0.96 | 2.17E-05 | 3.44E-03 |
| RP11-107E5.2 |  | -0.95 | 1.46E-04 | 1.26E-02 |
| ZNF628 | zinc finger protein 628 | -0.95 | 6.43E-04 | 3.54E-02 |
| IFI6 | interferon alpha inducible protein 6 | -0.94 | 5.64E-05 | 6.55E-03 |
| LTBP4 | latent transforming growth factor beta binding protein 4 | -0.94 | 1.99E-05 | 3.30E-03 |
| LINC02086 | long intergenic non-protein coding RNA 2086 | -0.93 | 3.19E-06 | 8.30E-04 |
| ADRB2 | adrenoceptor beta 2 | -0.93 | 5.91E-07 | 2.18E-04 |
| DYRK1B | dual specificity tyrosine phosphorylation regulated kinase 1B | -0.93 | 7.99E-05 | 8.31E-03 |
| LLGL2 | LLGL2, scribble cell polarity complex component | -0.93 | 5.93E-06 | 1.30E-03 |
| TNRC18 | trinucleotide repeat containing 18 | -0.93 | 1.58E-05 | 2.84E-03 |
| TGFB1 | transforming growth factor beta 1 | -0.93 | 2.04E-06 | 6.33E-04 |
| MYO1G | myosin IG | -0.92 | 2.95E-07 | 1.20E-04 |
| CST7 | cystatin F | -0.92 | 3.03E-06 | 8.03E-04 |
| CACNA2D2 | calcium voltage-gated channel auxiliary subunit alpha2delta 2 | -0.92 | 2.82E-07 | 1.18E-04 |
| ABHD17A | abhydrolase domain containing 17A | -0.92 | 1.74E-05 | 2.99E-03 |
| USP18 | ubiquitin specific peptidase 18 | -0.92 | 5.89E-04 | 3.32E-02 |
| SPATA2L | spermatogenesis associated 2 like | -0.91 | 6.70E-04 | 3.68E-02 |
| IER5L | immediate early response 5 like | -0.91 | 1.53E-04 | 1.31E-02 |
| ADAM8 | ADAM metallopeptidase domain 8 | -0.91 | 2.68E-06 | 7.79E-04 |
| FOSL2 | FOS like 2, AP-1 transcription factor subunit | -0.90 | 1.37E-05 | 2.51E-03 |
| MUC16 | mucin 16, cell surface associated | -0.89 | 1.29E-04 | 1.18E-02 |
| TOGARAM2 | TOG array regulator of axonemal microtubules 2 | -0.89 | 5.43E-05 | 6.47E-03 |
| MARK4 | microtubule affinity regulating kinase 4 | -0.88 | 2.49E-05 | 3.79E-03 |
| MVD | mevalonate diphosphate decarboxylase | -0.88 | 2.81E-04 | 1.99E-02 |
| MEF2D | myocyte enhancer factor 2D | -0.88 | 1.61E-04 | 1.35E-02 |
| VPS37B | VPS37B, ESCRT-I subunit | -0.88 | 2.21E-04 | 1.69E-02 |
| KIF19 | kinesin family member 19 | -0.87 | 5.52E-06 | 1.24E-03 |
| IRF7 | interferon regulatory factor 7 | -0.87 | 1.97E-04 | 1.57E-02 |
| TNFSF9 | TNF superfamily member 9 | -0.87 | 4.70E-05 | 5.90E-03 |
| TRGJ2 | T cell receptor gamma joining 2 | -0.86 | 2.54E-04 | 1.87E-02 |
| RHBDF2 | rhomboid 5 homolog 2 | -0.86 | 4.76E-05 | 5.90E-03 |
| PLA2G16 | phospholipase A2 group XVI | -0.86 | 4.76E-05 | 5.90E-03 |
| SYNE1 | spectrin repeat containing nuclear envelope protein 1 | -0.86 | 1.53E-05 | 2.78E-03 |
| DUSP8 | dual specificity phosphatase 8 | -0.86 | 1.85E-04 | 1.51E-02 |
| LDLR | low density lipoprotein receptor | -0.85 | 5.92E-05 | 6.82E-03 |
| PRR12 | proline rich 12 | -0.85 | 3.68E-04 | 2.45E-02 |
| NSMF | NMDA receptor synaptonuclear signaling and neuronal migration factor | -0.85 | 1.43E-04 | 1.24E-02 |
| RAB11FIP5 | RAB11 family interacting protein 5 | -0.85 | 2.52E-06 | 7.45E-04 |
| CHST12 | carbohydrate sulfotransferase 12 | -0.84 | 7.12E-05 | 7.77E-03 |
| RAP1GAP2 | RAP1 GTPase activating protein 2 | -0.84 | 9.20E-06 | 1.83E-03 |
| TBX21 | T-box 21 | -0.84 | 5.44E-06 | 1.24E-03 |
| ADAP1 | ArfGAP with dual PH domains 1 | -0.84 | 1.02E-03 | 5.00E-02 |
| LITAF | lipopolysaccharide induced TNF factor | -0.84 | 3.54E-07 | 1.41E-04 |
| DMWD | DM1 locus, WD repeat containing | -0.84 | 1.09E-04 | 1.08E-02 |
| RAB15 | RAB15, member RAS oncogene family | -0.84 | 1.37E-04 | 1.22E-02 |
| SESN2 | sestrin 2 | -0.83 | 4.30E-04 | 2.71E-02 |
| FLNA | filamin A | -0.83 | 2.15E-05 | 3.44E-03 |
| VPS18 | VPS18, CORVET/HOPS core subunit | -0.83 | 2.99E-05 | 4.34E-03 |
| CTSW | cathepsin W | -0.83 | 2.79E-06 | 7.95E-04 |
| RUNX3 | runt related transcription factor 3 | -0.83 | 1.10E-04 | 1.08E-02 |
| ZBTB7A | zinc finger and BTB domain containing 7A | -0.83 | 1.89E-04 | 1.53E-02 |
| SCAMP4 | secretory carrier membrane protein 4 | -0.82 | 2.49E-04 | 1.86E-02 |
| RNF166 | ring finger protein 166 | -0.82 | 1.38E-04 | 1.22E-02 |
| SYTL3 | synaptotagmin like 3 | -0.82 | 2.16E-05 | 3.44E-03 |
| ZNF668 | zinc finger protein 668 | -0.82 | 6.25E-04 | 3.47E-02 |
| GPR153 | G protein-coupled receptor 153 | -0.82 | 3.19E-05 | 4.59E-03 |
| UBE2Q2P1 | ubiquitin conjugating enzyme E2 Q2 pseudogene 1 | -0.82 | 5.49E-04 | 3.16E-02 |
| RPTOR | regulatory associated protein of MTOR complex 1 | -0.81 | 3.62E-04 | 2.42E-02 |
| PPP2R2B | protein phosphatase 2 regulatory subunit Bbeta | -0.81 | 4.83E-05 | 5.93E-03 |
| RAPGEF1 | Rap guanine nucleotide exchange factor 1 | -0.80 | 5.51E-05 | 6.50E-03 |
| DENND3 | DENN domain containing 3 | -0.80 | 4.48E-04 | 2.77E-02 |
| PDE1B | phosphodiesterase 1B | -0.80 | 8.68E-04 | 4.42E-02 |
| AC144652.1 | uncharacterized LOC105375591 | -0.79 | 3.28E-05 | 4.67E-03 |
| ARHGAP10 | Rho GTPase activating protein 10 | -0.79 | 1.24E-05 | 2.34E-03 |
| SBK1 | SH3 domain binding kinase 1 | -0.79 | 4.93E-04 | 2.95E-02 |
| TAPBP | TAP binding protein | -0.79 | 1.11E-04 | 1.08E-02 |
| S1PR5 | sphingosine-1-phosphate receptor 5 | -0.79 | 3.87E-05 | 5.36E-03 |
| CRIP1 | cysteine rich protein 1 | -0.78 | 5.69E-04 | 3.24E-02 |
| TTC38 | tetratricopeptide repeat domain 38 | -0.78 | 6.70E-05 | 7.61E-03 |
| SIPA1 | signal-induced proliferation-associated 1 | -0.78 | 8.75E-04 | 4.43E-02 |
| SH3BGRL3 | SH3 domain binding glutamate rich protein like 3 | -0.77 | 5.44E-05 | 6.47E-03 |
| DGKQ | diacylglycerol kinase theta | -0.77 | 9.49E-04 | 4.73E-02 |
| ATP2A3 | ATPase sarcoplasmic/endoplasmic reticulum Ca2+ transporting 3 | -0.77 | 4.25E-05 | 5.67E-03 |
| INTS1 | integrator complex subunit 1 | -0.77 | 5.23E-05 | 6.32E-03 |
| MAPKAPK2 | mitogen-activated protein kinase-activated protein kinase 2 | -0.76 | 1.37E-04 | 1.22E-02 |
| DNMBP | dynamin binding protein | -0.76 | 1.92E-04 | 1.54E-02 |
| PATL2 | PAT1 homolog 2 | -0.76 | 5.60E-06 | 1.24E-03 |
| IFNLR1 | interferon lambda receptor 1 | -0.75 | 4.29E-05 | 5.68E-03 |
| TLR3 | toll like receptor 3 | -0.75 | 6.37E-04 | 3.52E-02 |
| NCOR2 | nuclear receptor corepressor 2 | -0.75 | 5.60E-04 | 3.20E-02 |
| EZR | ezrin | -0.75 | 7.32E-04 | 3.91E-02 |
| SSBP3 | single stranded DNA binding protein 3 | -0.75 | 7.05E-05 | 7.77E-03 |
| MCOLN2 | mucolipin 2 | -0.75 | 4.43E-04 | 2.76E-02 |
| SSBP4 | single stranded DNA binding protein 4 | -0.74 | 5.27E-04 | 3.07E-02 |
| SREBF2 | sterol regulatory element binding transcription factor 2 | -0.74 | 5.39E-04 | 3.12E-02 |
| MATK | megakaryocyte-associated tyrosine kinase | -0.74 | 4.72E-04 | 2.88E-02 |
| C1orf21 | chromosome 1 open reading frame 21 | -0.74 | 5.07E-04 | 2.99E-02 |
| ITGB2 | integrin subunit beta 2 | -0.74 | 2.45E-05 | 3.79E-03 |
| RGS9 | regulator of G protein signaling 9 | -0.74 | 2.94E-05 | 4.34E-03 |
| NOP14-AS1 | NOP14 antisense RNA 1 | -0.73 | 4.88E-04 | 2.94E-02 |
| FRMPD3 | FERM and PDZ domain containing 3 | -0.73 | 2.62E-04 | 1.92E-02 |
| LRRC8A | leucine rich repeat containing 8 VRAC subunit A | -0.72 | 7.58E-05 | 8.00E-03 |
| GAB3 | GRB2 associated binding protein 3 | -0.72 | 2.93E-04 | 2.06E-02 |
| EPN2 | epsin 2 | -0.72 | 5.96E-04 | 3.35E-02 |
| KDM4B | lysine demethylase 4B | -0.72 | 4.13E-04 | 2.66E-02 |
| NBEAL2 | neurobeachin like 2 | -0.71 | 1.07E-04 | 1.07E-02 |
| CTD-2377D24.8 |  | -0.71 | 4.44E-04 | 2.76E-02 |
| PREX1 | phosphatidylinositol-3,4,5-trisphosphate dependent Rac exchange factor 1 | -0.71 | 1.25E-05 | 2.34E-03 |
| ORAI1 | ORAI calcium release-activated calcium modulator 1 | -0.71 | 2.15E-04 | 1.65E-02 |
| ATL1 | atlastin GTPase 1 | -0.71 | 5.56E-05 | 6.51E-03 |
| HNRNPLL | heterogeneous nuclear ribonucleoprotein L like | -0.70 | 5.02E-04 | 2.97E-02 |
| BCL9L | B cell CLL/lymphoma 9 like | -0.70 | 2.81E-04 | 1.99E-02 |
| MPST | mercaptopyruvate sulfurtransferase | -0.70 | 8.84E-04 | 4.45E-02 |
| CCL5 | C-C motif chemokine ligand 5 | -0.70 | 1.06E-04 | 1.06E-02 |
| ITGAL | integrin subunit alpha L | -0.69 | 2.68E-04 | 1.94E-02 |
| PTMS | parathymosin | -0.69 | 2.47E-05 | 3.79E-03 |
| GNG2 | G protein subunit gamma 2 | -0.69 | 2.48E-05 | 3.79E-03 |
| GLB1L2 | galactosidase beta 1 like 2 | -0.68 | 8.49E-06 | 1.72E-03 |
| GALNT3 | polypeptide N-acetylgalactosaminyltransferase 3 | -0.68 | 4.59E-04 | 2.82E-02 |
| CCDC88C | coiled-coil domain containing 88C | -0.68 | 7.96E-04 | 4.10E-02 |
| CDYL2 | chromodomain Y like 2 | -0.68 | 5.70E-04 | 3.24E-02 |
| SH3RF2 | SH3 domain containing ring finger 2 | -0.67 | 1.70E-06 | 5.76E-04 |
| SLC15A4 | solute carrier family 15 member 4 | -0.67 | 7.92E-04 | 4.09E-02 |
| LSS | lanosterol synthase | -0.67 | 1.60E-04 | 1.35E-02 |
| LSP1 | lymphocyte-specific protein 1 | -0.67 | 2.07E-04 | 1.60E-02 |
| PXN | paxillin | -0.66 | 4.02E-04 | 2.61E-02 |
| FCRL6 | Fc receptor like 6 | -0.66 | 1.92E-04 | 1.54E-02 |
| LY6E | lymphocyte antigen 6 family member E | -0.66 | 6.85E-04 | 3.73E-02 |
| AL928654.3 |  | -0.66 | 7.49E-05 | 7.96E-03 |
| SOX13 | SRY-box 13 | -0.66 | 5.82E-04 | 3.29E-02 |
| GALNT10 | polypeptide N-acetylgalactosaminyltransferase 10 | -0.65 | 1.37E-04 | 1.22E-02 |
| PRELID2 | PRELI domain containing 2 | -0.65 | 4.56E-05 | 5.90E-03 |
| NAA50 | N(alpha)-acetyltransferase 50, NatE catalytic subunit | -0.64 | 4.05E-04 | 2.62E-02 |
| MYO9B | myosin IXB | -0.64 | 1.52E-04 | 1.30E-02 |
| NFATC2 | nuclear factor of activated T cells 2 | -0.64 | 4.28E-04 | 2.71E-02 |
| AXIN1 | axin 1 | -0.64 | 4.23E-04 | 2.69E-02 |
| JAG2 | jagged 2 | -0.64 | 7.87E-04 | 4.08E-02 |
| BHLHE40 | basic helix-loop-helix family member e40 | -0.64 | 3.94E-04 | 2.59E-02 |
| ADGRG5 | adhesion G protein-coupled receptor G5 | -0.63 | 1.01E-03 | 4.98E-02 |
| INSIG1 | insulin induced gene 1 | -0.63 | 7.72E-04 | 4.05E-02 |
| SLC20A1 | solute carrier family 20 member 1 | -0.62 | 2.85E-04 | 2.01E-02 |
| RAP2A | RAP2A, member of RAS oncogene family | -0.62 | 2.07E-04 | 1.60E-02 |
| RNF213 | ring finger protein 213 | -0.61 | 1.64E-04 | 1.37E-02 |
| RDH10 | retinol dehydrogenase 10 | -0.61 | 9.23E-04 | 4.61E-02 |
| CCL4 | C-C motif chemokine ligand 4 | -0.61 | 7.15E-05 | 7.77E-03 |
| ZNF683 | zinc finger protein 683 | -0.61 | 6.14E-05 | 7.02E-03 |
| DDN-AS1 | DDN and PRKAG1 antisense RNA 1 | -0.61 | 2.75E-04 | 1.99E-02 |
| PLEKHA2 | pleckstrin homology domain containing A2 | -0.61 | 3.22E-04 | 2.22E-02 |
| PTPRJ | protein tyrosine phosphatase, receptor type J | -0.60 | 1.02E-03 | 5.00E-02 |
| SHISA5 | shisa family member 5 | -0.60 | 7.80E-04 | 4.07E-02 |
| SFT2D2 | SFT2 domain containing 2 | -0.58 | 6.75E-04 | 3.69E-02 |
| CLSTN1 | calsyntenin 1 | -0.58 | 3.37E-04 | 2.31E-02 |
| SLC1A7 | solute carrier family 1 member 7 | -0.58 | 2.06E-05 | 3.38E-03 |
| GPRIN1 | G protein regulated inducer of neurite outgrowth 1 | -0.57 | 8.70E-05 | 8.87E-03 |
| PTP4A2 | protein tyrosine phosphatase type IVA, member 2 | -0.57 | 4.53E-04 | 2.79E-02 |
| HLA-DPA1 | major histocompatibility complex, class II, DP alpha 1 | -0.56 | 8.60E-05 | 8.84E-03 |
| MSN | moesin | -0.55 | 8.20E-04 | 4.21E-02 |
| TMCC3 | transmembrane and coiled-coil domain family 3 | -0.54 | 2.00E-04 | 1.58E-02 |
| FGFBP2 | fibroblast growth factor binding protein 2 | -0.52 | 7.48E-04 | 3.96E-02 |
| GZMB | granzyme B | -0.50 | 6.04E-04 | 3.38E-02 |
| MSC | musculin | -0.50 | 5.21E-04 | 3.05E-02 |
| AC093616.1 | anaphase-promoting complex subunit 1-like | -0.48 | 6.23E-04 | 3.47E-02 |
| MYO3B | myosin IIIB | -0.41 | 1.01E-07 | 4.75E-05 |
| DPY19L1P1 | DPY19L1 pseudogene 1 | -0.37 | 6.92E-04 | 3.76E-02 |
| PROK2 | prokineticin 2 | -0.24 | 1.76E-06 | 5.76E-04 |
| DUSP4 | dual specificity phosphatase 4 | -0.21 | 4.75E-04 | 2.88E-02 |
| GPR27 | G protein-coupled receptor 27 | -0.20 | 1.94E-05 | 3.25E-03 |
| LINC00355 | long intergenic non-protein coding RNA 355 | -0.10 | 3.71E-04 | 2.46E-02 |
| HBB | hemoglobin subunit beta | -0.07 | 1.71E-05 | 2.98E-03 |
| GSTM1 | glutathione S-transferase mu 1 | 0.07 | 2.85E-06 | 7.98E-04 |
| PDCD6IPP2 | PDCD6IP pseudogene 2 | 0.10 | 4.09E-04 | 2.64E-02 |
| C21orf33 | chromosome 21 open reading frame 33 | 0.20 | 8.96E-04 | 4.50E-02 |
| CCR9 | C-C motif chemokine receptor 9 | 0.29 | 4.96E-04 | 2.96E-02 |
| ADAMTS1 | ADAM metallopeptidase with thrombospondin type 1 motif 1 | 0.30 | 3.58E-04 | 2.40E-02 |
| RP11-65I12.1 |  | 0.37 | 8.07E-06 | 1.67E-03 |
| ARPIN | actin related protein 2/3 complex inhibitor | 0.43 | 8.61E-05 | 8.84E-03 |
| SIGLEC7 | sialic acid binding Ig like lectin 7 | 0.45 | 5.38E-04 | 3.12E-02 |
| PTPN20 | protein tyrosine phosphatase, non-receptor type 20 | 0.49 | 9.90E-04 | 4.90E-02 |
| AL034550.2 | uncharacterized LOC101929698 | 0.54 | 7.82E-04 | 4.07E-02 |
| IGFBP7 | insulin like growth factor binding protein 7 | 0.58 | 2.01E-04 | 1.58E-02 |
| ZNF154 | zinc finger protein 154 | 0.59 | 5.56E-04 | 3.19E-02 |
| AP005131.6 |  | 0.61 | 3.42E-04 | 2.33E-02 |
| ICAM2 | intercellular adhesion molecule 2 | 0.62 | 8.70E-04 | 4.42E-02 |
| UXS1 | UDP-glucuronate decarboxylase 1 | 0.62 | 7.39E-04 | 3.93E-02 |
| ZMAT1 | zinc finger matrin-type 1 | 0.63 | 5.02E-04 | 2.97E-02 |
| DPY19L2 | dpy-19 like 2 | 0.63 | 3.10E-04 | 2.16E-02 |
| TXK | TXK tyrosine kinase | 0.65 | 4.41E-04 | 2.76E-02 |
| RNF165 | ring finger protein 165 | 0.66 | 6.98E-04 | 3.76E-02 |
| CHPT1 | choline phosphotransferase 1 | 0.67 | 2.46E-04 | 1.84E-02 |
| STRBP | spermatid perinuclear RNA binding protein | 0.67 | 2.53E-04 | 1.87E-02 |
| DGKA | diacylglycerol kinase alpha | 0.68 | 5.14E-04 | 3.02E-02 |
| FAT4 | FAT atypical cadherin 4 | 0.68 | 1.27E-04 | 1.17E-02 |
| B4GALT6 | beta-1,4-galactosyltransferase 6 | 0.69 | 1.14E-04 | 1.11E-02 |
| DIP2C | disco interacting protein 2 homolog C | 0.69 | 2.81E-04 | 1.99E-02 |
| TLE1 | transducin like enhancer of split 1 | 0.70 | 6.97E-04 | 3.76E-02 |
| PAX8-AS1 | PAX8 antisense RNA 1 | 0.70 | 1.22E-09 | 1.15E-06 |
| LAPTM4B | lysosomal protein transmembrane 4 beta | 0.70 | 1.68E-04 | 1.39E-02 |
| NSUN5P1 | NOP2/Sun RNA methyltransferase family member 5 pseudogene 1 | 0.72 | 3.21E-04 | 2.22E-02 |
| SH2D1B | SH2 domain containing 1B | 0.72 | 3.02E-06 | 8.03E-04 |
| SSBP2 | single stranded DNA binding protein 2 | 0.72 | 7.33E-04 | 3.91E-02 |
| SH3YL1 | SH3 and SYLF domain containing 1 | 0.72 | 3.54E-04 | 2.39E-02 |
| TRDC | T cell receptor delta constant | 0.72 | 1.16E-05 | 2.22E-03 |
| CD27 | CD27 molecule | 0.73 | 2.64E-04 | 1.93E-02 |
| RP11-28F1.2 |  | 0.73 | 4.78E-04 | 2.89E-02 |
| CASP10 | caspase 10 | 0.73 | 9.73E-04 | 4.83E-02 |
| GSDMB | gasdermin B | 0.74 | 1.19E-04 | 1.14E-02 |
| ZFYVE9 | zinc finger FYVE-type containing 9 | 0.74 | 7.32E-05 | 7.84E-03 |
| ZNF204P | zinc finger protein 204, pseudogene | 0.75 | 3.55E-04 | 2.39E-02 |
| SATB1-AS1 | SATB1 antisense RNA 1 | 0.76 | 7.14E-05 | 7.77E-03 |
| ZNF542P | zinc finger protein 542, pseudogene | 0.76 | 4.15E-05 | 5.59E-03 |
| HIPK2 | homeodomain interacting protein kinase 2 | 0.76 | 1.20E-04 | 1.14E-02 |
| SLC7A6 | solute carrier family 7 member 6 | 0.77 | 1.08E-04 | 1.08E-02 |
| TMIGD2 | transmembrane and immunoglobulin domain containing 2 | 0.77 | 1.72E-05 | 2.98E-03 |
| LGALS9B | galectin 9B | 0.77 | 4.92E-05 | 5.99E-03 |
| PDE4DIP | phosphodiesterase 4D interacting protein | 0.78 | 4.20E-04 | 2.69E-02 |
| CHMP7 | charged multivesicular body protein 7 | 0.78 | 3.99E-05 | 5.43E-03 |
| ZNF781 | zinc finger protein 781 | 0.78 | 8.84E-04 | 4.45E-02 |
| AKAP7 | A-kinase anchoring protein 7 | 0.79 | 2.09E-04 | 1.61E-02 |
| ZNF793 | zinc finger protein 793 | 0.79 | 3.46E-05 | 4.88E-03 |
| KLHL6 | kelch like family member 6 | 0.79 | 3.64E-05 | 5.09E-03 |
| ZFP28 | ZFP28 zinc finger protein | 0.80 | 1.85E-04 | 1.51E-02 |
| LINGO2 | leucine rich repeat and Ig domain containing 2 | 0.80 | 1.40E-04 | 1.22E-02 |
| TBC1D4 | TBC1 domain family member 4 | 0.81 | 7.01E-05 | 7.77E-03 |
| ZNF717 | zinc finger protein 717 | 0.81 | 1.21E-04 | 1.14E-02 |
| GSTM2 | glutathione S-transferase mu 2 | 0.82 | 1.26E-04 | 1.17E-02 |
| JAML | junction adhesion molecule like | 0.82 | 1.86E-04 | 1.51E-02 |
| DHX32 | DEAH-box helicase 32 (putative) | 0.82 | 4.25E-04 | 2.70E-02 |
| CLN5 | CLN5, intracellular trafficking protein | 0.82 | 2.54E-04 | 1.87E-02 |
| AP000977.1 | uncharacterized LOC105369536 | 0.84 | 2.28E-04 | 1.73E-02 |
| LAT2 | linker for activation of T cells family member 2 | 0.84 | 1.24E-04 | 1.16E-02 |
| FAM19A1 | family with sequence similarity 19 member A1, C-C motif chemokine like | 0.85 | 1.30E-04 | 1.19E-02 |
| LINC00402 | long intergenic non-protein coding RNA 402 | 0.85 | 4.70E-04 | 2.87E-02 |
| CHST2 | carbohydrate sulfotransferase 2 | 0.85 | 1.41E-04 | 1.23E-02 |
| IL7R | interleukin 7 receptor | 0.86 | 4.70E-05 | 5.90E-03 |
| RPL5P13 | ribosomal protein L5 pseudogene 13 | 0.87 | 2.60E-05 | 3.93E-03 |
| SYNJ2 | synaptojanin 2 | 0.88 | 3.01E-04 | 2.10E-02 |
| TRBV30 | T cell receptor beta variable 30 (gene/pseudogene) | 0.89 | 1.06E-05 | 2.06E-03 |
| LINC00402 | long intergenic non-protein coding RNA 402 | 0.89 | 4.75E-05 | 5.90E-03 |
| AC241952.1 | phosphodiesterase 4D interacting protein-like | 0.89 | 2.07E-04 | 1.60E-02 |
| COLQ | collagen like tail subunit of asymmetric acetylcholinesterase | 0.89 | 1.81E-06 | 5.82E-04 |
| CAMSAP2 | calmodulin regulated spectrin associated protein family member 2 | 0.90 | 3.42E-04 | 2.33E-02 |
| CRYBG3 | crystallin beta-gamma domain containing 3 | 0.90 | 1.39E-04 | 1.22E-02 |
| GABPB1-AS1 | GABPB1 antisense RNA 1 | 0.93 | 2.29E-04 | 1.73E-02 |
| AC012636.1 | uncharacterized LOC101929215 | 0.93 | 1.24E-04 | 1.16E-02 |
| MBOAT1 | membrane bound O-acyltransferase domain containing 1 | 0.94 | 7.69E-04 | 4.04E-02 |
| TNFRSF10D | TNF receptor superfamily member 10d | 0.96 | 1.60E-05 | 2.85E-03 |
| FAM153A | family with sequence similarity 153 member A | 0.97 | 1.88E-07 | 8.34E-05 |
| KLRB1 | killer cell lectin like receptor B1 | 0.98 | 6.84E-09 | 5.16E-06 |
| AL139415.1 |  | 1.00 | 6.05E-06 | 1.31E-03 |
| AC037198.1 |  | 1.01 | 2.80E-04 | 1.99E-02 |
| EPHA1 | EPH receptor A1 | 1.02 | 8.53E-06 | 1.72E-03 |
| SPART | spartin | 1.02 | 2.65E-07 | 1.14E-04 |
| DGKK | diacylglycerol kinase kappa | 1.02 | 1.16E-04 | 1.12E-02 |
| PRSS21 | serine protease 21 | 1.03 | 4.00E-04 | 2.61E-02 |
| CA6 | carbonic anhydrase 6 | 1.04 | 7.79E-05 | 8.16E-03 |
| CLUAP1 | clusterin associated protein 1 | 1.04 | 5.19E-06 | 1.23E-03 |
| IL6R | interleukin 6 receptor | 1.04 | 5.22E-06 | 1.23E-03 |
| HMCN1 | hemicentin 1 | 1.05 | 3.81E-04 | 2.51E-02 |
| CD200R1 | CD200 receptor 1 | 1.06 | 1.65E-04 | 1.37E-02 |
| EPHA1-AS1 | EPHA1 antisense RNA 1 | 1.07 | 4.91E-06 | 1.20E-03 |
| KLF13 | Kruppel like factor 13 | 1.09 | 3.95E-05 | 5.43E-03 |
| VLDLR | very low density lipoprotein receptor | 1.10 | 7.67E-04 | 4.04E-02 |
| ACSL6 | acyl-CoA synthetase long chain family member 6 | 1.10 | 4.26E-08 | 2.57E-05 |
| APP | amyloid beta precursor protein | 1.11 | 5.38E-08 | 3.03E-05 |
| TMEM176A | transmembrane protein 176A | 1.12 | 1.02E-06 | 3.57E-04 |
| CLIC5 | chloride intracellular channel 5 | 1.14 | 2.97E-05 | 4.34E-03 |
| TMTC1 | transmembrane and tetratricopeptide repeat containing 1 | 1.15 | 7.03E-04 | 3.78E-02 |
| SPECC1 | sperm antigen with calponin homology and coiled-coil domains 1 | 1.16 | 3.02E-06 | 8.03E-04 |
| TRAV5 | T cell receptor alpha variable 5 | 1.17 | 4.48E-05 | 5.88E-03 |
| XKR9 | XK related 9 | 1.20 | 4.74E-06 | 1.19E-03 |
| AKR1C3 | aldo-keto reductase family 1 member C3 | 1.28 | 1.73E-06 | 5.76E-04 |
| COX10-AS1 | COX10 antisense RNA 1 | 1.29 | 1.96E-04 | 1.57E-02 |
| TMEM176B | transmembrane protein 176B | 1.39 | 1.89E-09 | 1.68E-06 |
| GCSAM | germinal center associated signaling and motility | 1.43 | 6.31E-13 | 1.90E-09 |
| KLRF1 | killer cell lectin like receptor F1 | 1.60 | 8.92E-15 | 6.73E-11 |
| TRBV28 | T cell receptor beta variable 28 | 1.64 | 4.56E-10 | 5.73E-07 |
