# Supplementary material for: T-cell transcriptomics from peripheral blood highlights differences between polymyositis and dermatomyositis patients
Source: Arthritis Res Ther. 2018 Aug 29;20:188. doi: 10.1186/s13075-018-1688-7 (PMC6116372; doi:10.1186/s13075-018-1688-7)
Supplement: Supplementary file 5 — Gene Ontology biological processes for the differentially expressed genes in CD8+ T cells of PM and DM patients. Table S10 shows the genes mapped to the enriched GO biological processes for the differentially expressed genes in CD8+ T cells of PM and DM patients. (DOCX 17 kb) [file 13075_2018_1688_MOESM5_ESM.docx]

**Table S10**

The genes mapped to the enriched GO biological processes for the differentially expressed genes in CD8+ T cells of PM and DM patients.

| **GO biological process complete** | **Genes** |
| --- | --- |
| lymphocyte migration (GO:0072676) | ADAM8, CCL5, TBX21, MYO1G, CCL4 |
| regulation of T cell differentiation (GO:0045580) | ZNF683, TNFSF9, SMAD7, ADAM8, NFATC2, TGFB1, TBX21, SOX13, CD27 |
| regulation of lymphocyte differentiation (GO:0045619) | ZNF683, TNFSF9, SMAD7, ADAM8, NFATC2, TGFB1, TBX21, SOX13, CD27 |
| myeloid leukocyte migration (GO:0097529) | ITGB2, IL6R, CCL5, MCOLN2, JAML, CCL4 |
| response to transforming growth factor beta (GO:0071559) | SMAD7, RUNX3, PDGFD, TGFB1, LTBP4, TGFBR3, PXN |
| regulation of leukocyte differentiation (GO:1902105) | ZNF683, TNFSF9, SMAD7, ADAM8, NFATC2, TGFB1, TBX21, TLR3, SOX13, CD27 |
| regulation of T cell activation (GO:0050863) | ZNF683, TNFSF9, SMAD7, ADAM8, NFATC2, TGFB1, TMIGD2, LAG3, CCL5, TBX21, SOX13, CD27 |
| leukocyte migration (GO:0050900) | ITGB2, IL6R, ITGAL, ADAM8, SLC7A6, TGFB1, CCL5, TBX21, MCOLN2, JAML, TNFRSF10D, MYO1G, CCL4 |
| positive regulation of GTPase activity (GO:0043547) | DNMBP, ADAP1, EPHA1, RAP1GAP2, RGS9, CCL5, TBC1D4, ARHGAP10, CCL4, GNAO1, SIPA1, RAPGEF1, LLGL2 |
| positive regulation of cell adhesion (GO:0045785) | FLNA, TNFSF9, SMAD7, EPHA1, ADAM8, PTPRJ, TGFB1, TMIGD2, CCL5, PLEKHA2, CD27, ADGRG1 |
| positive regulation of MAPK cascade (GO:0043410) | ADRB2, IL6R, EPHA1, MAPKAPK2, ADAM8, ZEB2, PROK2, PDGFD, TGFB1, CCL5, TNFRSF10D, TLR3, CD27, CCL4, RAPGEF1 |
| regulation of GTPase activity (GO:0043087) | DNMBP, ADAP1, EPHA1, RAP1GAP2, RGS9, CCL5, TBC1D4, ARHGAP10, CCL4, GNAO1, SIPA1, RAPGEF1, LLGL2 |
| regulation of cell activation (GO:0050865) | LDLR, ZNF683, ITGB2, TNFSF9, SMAD7, ADAM8, NFATC2, TGFB1, TMIGD2, LAG3, CCL5, TBX21, SOX13, TXK, CD27 |
| regulation of leukocyte activation (GO:0002694) | LDLR, ZNF683, ITGB2, TNFSF9, SMAD7, ADAM8, NFATC2, TGFB1, TMIGD2, LAG3, CCL5, TBX21, SOX13, CD27 |
| transmembrane receptor protein tyrosine kinase signaling pathway (GO:0007169) | ADRB2, EPHA1, SH2D2A, MAPKAPK2, PTPRJ, MATK, PDGFD, DGKQ, TGFB1, GALNT3, TXK, PXN, RAPGEF1 |
| regulation of MAPK cascade (GO:0043408) | ADRB2, IL6R, RAP2A, EPHA1, MAPKAPK2, ADAM8, ZEB2, PTPRJ, PROK2, PDGFD, TGFB1, CCL5, DUSP8, TNFRSF10D, TLR3, CD27, CCL4, RAPGEF1 |
| regulation of cell adhesion (GO:0030155) | FLNA, TNFSF9, SMAD7, EPHA1, ADAM8, PTPRJ, TGFB1, TMIGD2, LAG3, CCL5, TBX21, PLEKHA2, CD27, ADGRG1, SIPA1 |
| enzyme linked receptor protein signaling pathway (GO:0007167) | ADRB2, SMAD7, EPHA1, SH2D2A, MAPKAPK2, PTPRJ, MATK, PDGFD, DGKQ, TGFB1, GALNT3, LTBP4, TGFBR3, TXK, PXN, RAPGEF1 |
| cell migration (GO:0016477) | FLNA, ITGB2, IL6R, ITGAL, ADAM8, ZEB2, SLC7A6, NFATC2, CCL5, TBX21, MCOLN2, TGFBR3, JAML, TNFRSF10D, MYO1G, PXN, CCL4, ADGRG1 |
| positive regulation of immune system process (GO:0002684) | ZNF683, KLHL6, ITGB2, TNFSF9, IL6R, MAPKAPK2, ADAM8, PTPRJ, PDGFD, NFATC2, TGFB1, TMIGD2, MUC16, LAG3, CCL5, TBX21, TLR3, MYO1G, TXK, CD27, CCL4, RAPGEF1 |
| regulation of immune system process (GO:0002682) | ZNF683, LDLR, KLHL6, ITGB2, TNFSF9, IL6R, SMAD7, PAX5, MAPKAPK2, ITGAL, ADAM8, PTPRJ, PDGFD, NFATC2, TGFB1, TMIGD2, MUC16, LAG3, CCL5, TBX21, JAML, IFNLR1, TLR3, MYO1G, SOX13, TXK, GCSAM, CD27, CCL4, KLF13 |
| positive regulation of intracellular signal transduction (GO:1902533) | ADRB2, FLNA, IL6R, EPHA1, MAPKAPK2, ADAM8, ZEB2, PTPRJ, PROK2, PDGFD, TGFB1, CCL5, CASP10, TNFRSF10D, TLR3, SLC20A1, CD27, CCL4, ADGRG1, RAPGEF1 |
| regulation of immune response (GO:0050776) | ZNF683, KLHL6, ITGB2, SMAD7, PAX5, MAPKAPK2, ITGAL, ADAM8, PTPRJ, NFATC2, TGFB1, MUC16, LAG3, CCL5, TBX21, JAML, TLR3, MYO1G, TXK, GCSAM, RAPGEF1 |
| positive regulation of catalytic activity (GO:0043085) | ADRB2, DNMBP, IL6R, ADAP1, EPHA1, MAPKAPK2, ADAM8, ZEB2, PROK2, PDGFD, RAP1GAP2, DGKQ, TGFB1, RGS9, CCL5, TBC1D4, CASP10, TLR3, ARHGAP10, TXK, CCL4, GNAO1, SIPA1, RAPGEF1, LLGL2 |
| immune response (GO:0006955) | ZNF683, GZMB, KLHL6, ITGB2, TNFSF9, IL6R, CRIP1, PAX5, ITGAL, ADAM8, PTPRJ, MATK, TGFB1, NBEAL2, TMIGD2, CTSW, CCL5, CST7, TBKBP1, FRMPD3, MCOLN2, SLC15A4, TGFBR3, TNFRSF10D, IFNLR1, TLR3, MYO1G, TXK, CD27, CCL4, GZMH |
| cell surface receptor signaling pathway (GO:0007166) | ADRB2, ZNF683, FLNA, KLHL6, ITGB2, TNFSF9, IL6R, ADAP1, SMAD7, EPHA1, SH2D2A, ADGRG5, MAPKAPK2, GNG2, ITGAL, PTPRJ, MATK, PDGFD, DGKQ, NFATC2, TGFB1, MUC16, GALNT3, LAG3, LTBP4, CCL5, CASP10, TGFBR3, TNFRSF10D, IFNLR1, TLR3, MYO1G, TXK, CD27, PXN, CCL4, GNAO1, ADGRG1, FAM53B, RAPGEF1 |
| positive regulation of signal transduction (GO:0009967) | ADRB2, FLNA, GZMB, IL6R, EPHA1, SH2D2A, MAPKAPK2, ADAM8, ZEB2, PTPRJ, PROK2, PDGFD, TGFB1, CCL5, CASP10, TGFBR3, TNFRSF10D, TLR3, SLC20A1, TXK, CD27, GPR27, CCL4, ADGRG1, FAM53B, RAPGEF1 |
| positive regulation of molecular function (GO:0044093) | ADRB2, DNMBP, ITGB2, IL6R, ADAP1, EPHA1, MAPKAPK2, ADAM8, ZEB2, PROK2, PDGFD, RAP1GAP2, DGKQ, TGFB1, RGS9, CCL5, TBC1D4, CASP10, CLN5, AKAP7, TLR3, ARHGAP10, TXK, CCL4, GNAO1, SIPA1, RAPGEF1, LLGL2 |
| regulation of intracellular signal transduction (GO:1902531) | ADRB2, FLNA, DNMBP, IL6R, RAP2A, ADAP1, EPHA1, SESN2, MAPKAPK2, ADAM8, ZEB2, PTPRJ, PROK2, PDGFD, RAP1GAP2, DGKQ, TGFB1, CCL5, CASP10, AKAP7, DUSP8, TNFRSF10D, TLR3, SLC20A1, ARHGAP10, CD27, CCL4, ADGRG1, SIPA1, RAPGEF1 |
| immune system process (GO:0002376) | ZNF683, GZMB, KLHL6, ITGB2, TNFSF9, IL6R, RUNX3, SSBP3, CRIP1, GAB3, PAX5, MAPKAPK2, ITGAL, ADAM8, PTPRJ, MATK, SLC7A6, NFATC2, TGFB1, NBEAL2, TMIGD2, MUC16, LAG3, CTSW, CCL5, TBX21, CST7, TBKBP1, FRMPD3, MCOLN2, SLC15A4, TGFBR3, JAML, TNFRSF10D, IFNLR1, TLR3, MYO1G, TXK, PTMS, CD27, CCL4, GZMH |
| regulation of multicellular organismal development (GO:2000026) | ADRB2, ZNF683, LDLR, FLNA, ITGB2, TNFSF9, RAP2A, SMAD7, EPHA1, CAMSAP2, CASZ1, ADAM8, ZEB2, PROK2, PDGFD, RAP1GAP2, NFATC2, PLA2G16, TGFB1, TMIGD2, TBX21, TGFBR3, TLR3, SOX13, CD27, S1PR5, KLF13, ADGRG1, RAPGEF1 |
| positive regulation of response to stimulus (GO:0048584) | ADRB2, ZNF683, LDLR, FLNA, GZMB, KLHL6, ITGB2, IL6R, EPHA1, SH2D2A, MAPKAPK2, ADAM8, ZEB2, PTPRJ, PROK2, PDGFD, NFATC2, TGFB1, MUC16, LAG3, CCL5, TBX21, CASP10, TGFBR3, TNFRSF10D, TLR3, MYO1G, SLC20A1, TXK, CD27, GPR27, CCL4, ADGRG1, FAM53B, RAPGEF1 |
| regulation of catalytic activity (GO:0050790) | ADRB2, SH3RF2, DNMBP, IL6R, ADAP1, SMAD7, EPHA1, SESN2, MAPKAPK2, ADAM8, ZEB2, PTPRJ, PROK2, PDGFD, RAP1GAP2, DGKQ, MIDN, TGFB1, RGS9, CCL5, CST7, TBC1D4, CASP10, DUSP8, TNFRSF10D, TLR3, PPP2R2B, ARHGAP10, TXK, CD27, CCL4, GNAO1, SIPA1, RAPGEF1, LLGL2 |
| regulation of signal transduction (GO:0009966) | ADRB2, FLNA, DNMBP, GZMB, TNFSF9, IL6R, RAP2A, ADAP1, SMAD7, EPHA1, SESN2, SH2D2A, PAX5, MAPKAPK2, INSIG1, ADAM8, ZEB2, RHBDF2, PTPRJ, PROK2, PDGFD, RAP1GAP2, DGKQ, TGFB1, RGS9, LTBP4, CCL5, CASP10, AKAP7, DUSP8, TGFBR3, TNFRSF10D, TLR3, SLC20A1, ARHGAP10, TXK, GCSAM, CD27, GPR27, CCL4, ADGRG1, SIPA1, FAM53B, RAPGEF1, LLGL2 |
| regulation of developmental process (GO:0050793) | ADRB2, ZNF683, LDLR, FLNA, ITGB2, TNFSF9, IL6R, RAP2A, SMAD7, EPHA1, RUNX3, SSBP3, CAMSAP2, CASZ1, INSIG1, ADAM8, ZEB2, PROK2, PDGFD, RAP1GAP2, NFATC2, PLA2G16, TGFB1, TMIGD2, LTBP4, TBX21, CACNA2D2, TGFBR3, TLR3, SOX13, CD27, S1PR5, KLF13, ADGRG1, RAPGEF1 |
| regulation of signaling (GO:0023051) | ADRB2, FLNA, DNMBP, GZMB, TNFSF9, IL6R, RAP2A, ADAP1, RAB11FIP5, SMAD7, EPHA1, SESN2, SH2D2A, PAX5, MAPKAPK2, INSIG1, ADAM8, ZEB2, RHBDF2, PTPRJ, PROK2, PDGFD, RAP1GAP2, ATP1A3, DGKQ, MIDN, TGFB1, RGS9, LTBP4, CCL5, CASP10, CACNA2D2, AKAP7, DUSP8, TGFBR3, TNFRSF10D, TLR3, SLC20A1, ARHGAP10, TXK, GCSAM, CD27, GPR27, PXN, CCL4, SLC2A1, ADGRG1, SIPA1, FAM53B, RAPGEF1, LLGL2 |
| regulation of cell communication (GO:0010646) | ADRB2, FLNA, DNMBP, GZMB, TNFSF9, IL6R, RAP2A, ADAP1, RAB11FIP5, SMAD7, EPHA1, SESN2, SH2D2A, PAX5, MAPKAPK2, INSIG1, ADAM8, ZEB2, RHBDF2, PTPRJ, PROK2, PDGFD, RAP1GAP2, DGKQ, MIDN, TGFB1, RGS9, LTBP4, CCL5, CASP10, CACNA2D2, AKAP7, DUSP8, TGFBR3, TNFRSF10D, TLR3, SLC20A1, ARHGAP10, TXK, GCSAM, CD27, GPR27, PXN, CCL4, SLC2A1, ADGRG1, SIPA1, FAM53B, RAPGEF1, LLGL2 |
| regulation of response to stimulus (GO:0048583) | ADRB2, ZNF683, LDLR, FLNA, DNMBP, GZMB, KLHL6, ITGB2, TNFSF9, IL6R, RAP2A, ADAP1, SMAD7, EPHA1, SESN2, SH2D2A, PAX5, MAPKAPK2, ITGAL, INSIG1, ADAM8, ZEB2, RHBDF2, PTPRJ, PROK2, PDGFD, RAP1GAP2, DGKQ, NFATC2, TGFB1, MUC16, LAG3, RGS9, LTBP4, CCL5, TBX21, CASP10, AKAP7, DUSP8, TGFBR3, JAML, TNFRSF10D, IFNLR1, TLR3, MYO1G, SLC20A1, ARHGAP10, TXK, GCSAM, CD27, GPR27, PXN, CCL4, ADGRG1, SIPA1, FAM53B, RAPGEF1, LLGL2 |
| regulation of multicellular organismal process (GO:0051239) | ADRB2, ZNF683, LDLR, FLNA, ITGB2, TNFSF9, IL6R, RAP2A, RAP11FIP5, SMAD7, EPHA1, CAMSAP2, CASZ1, MAPKAPK2, ADAM8, ZEB2, PROK2, PDGFD, RAP1GAP2, ATP1A3, NFATC2, PLA2G16, TGFB1, TMIGD2, LAG3, TBX21, LAPTM4B, CACNA2D2, MCOLN2, TGFBR3, TLR3, SOX13, TXK, CD27, S1PR5, GNAO1, KLF13, ADGRG1, RAPGEF1 |
| regulation of molecular function (GO:0065009) | ADRB2, SH3RF2, FLNA, DNMBP, ITGB2, TNFSF9, IL6R, ADAP1, SMAD7, EPHA1, SESN2, MAPKAPK2, ADAM8, ZEB2, PTPRJ, PROK2, PDGFD, RAP1GAP2, DGKQ, MIDN, TGFB1, RGS9, CCL5, CST7, TBC1D4, CASP10, CLN5, BHLHE40, AKAP7, DUSP8, TGFBR3, TNFRSF10D, TLR3, PPP2R2B, ARHGAP10, TXK, CD27, CCL4, GNAO1, SIPA1, RAPGEF1, LLGL2 |
| signaling (GO:0023052) | ADRB2, ZNF683, FLNA, DNMBP, GZMB, KLHL6, ITGB2, TNFSF9, IL6R, RAP2A, GPR153, ADAP1, RAB11FIP5, SMAD7, EPHA1, SESN2, SH2D2A, CRIP1, ADGRG5, MAPKAPK2, GNG2, ITGAL, INSIG1, PTPRJ, PROK2, MATK, PDGFD, ATP1A3, DGKQ, NFATC2, TGFB1, TMIGD2, MUC16, GALNT3, LAG3, RGS9, LTBP4, CCL5, DGKA, CASP10, SYTL3, CACNA2D2, AKAP7, TGFBR3, TNFRSF10D, IFNLR1, TLR3, MYO1G, SLC20A1, ARHGAP10, TXK, CD27, S1PR5, GPR27, PXN, CCL4, GNAO1, FGFBP2, ADGRG1, SIPA1, FAM53B, RAPGEF1, GZMH |
| cell communication (GO:0007154) | ADRB2, ZNF683, FLNA, DNMBP, GZMB, KLHL6, ITGB2, TNFSF9, IL6R, RAP2A, GPR153, ADAP1, RAB11FIP5, SMAD7, EPHA1, SESN2, SH2D2A, CRIP1, ADGRG5, MAPKAPK2, GNG2, ITGAL, INSIG1, PTPRJ, PROK2, MATK, PDGFD, ATP1A3, DGKQ, NFATC2, TGFB1, TMIGD2, MUC16, GALNT3, LAG3, RGS9, LTBP4, CCL5, DGKA, CASP10, SYTL3, AKAP7, TGFBR3, TNFRSF10D, IFNLR1, TLR3, MYO1G, SLC20A1, ARHGAP10, TXK, CD27, S1PR5, GPR27, PXN, CCL4, GNAO1, SLC2A1, FGFBP2, ADGRG1, SIPA1, FAM53B, RAPGEF1, GZMH |
