# Supplementary material for: T-cell transcriptomics from peripheral blood highlights differences between polymyositis and dermatomyositis patients
Source: Arthritis Res Ther. 2018 Aug 29;20:188. doi: 10.1186/s13075-018-1688-7 (PMC6116372; doi:10.1186/s13075-018-1688-7)
Supplement: Supplementary file 6 — Differentially expressed genes in CD4+ T cells of HLA-DRB1*03-positive and -negative myositis patients. Table S11 and S12 provide differentially expressed genes for CD4+ T cells of HLA-DRB1*03-positive and -negative myositis patients at analytical stage 1 (including potential outliers) and analytical stage 2 (excluding potential outliers), respectively. (DOCX 16 kb) [file 13075_2018_1688_MOESM6_ESM.docx]

**Table S11**

Differentially expressed genes in CD4+ T cells of *HLA-DRB1**03 positive and negative myositis patients. Genes with a positive FC are higher expressed in *HLA-DRB1**03 positive patients and genes with a negative FC are higher expressed in *HLA-DRB1**03 negative patients. P-values were estimated by the Wald test in DESeq2 adjusted for gender, age group, diagnosis, and RIN value. A FDR threshold of 5% based on the method of Benjamini-Hochberg was used to identify significant differentially expressed genes. Abbreviations: FC: Fold Change.

| **Gene symbol** | **Gene name** | **Log2FC** | **P-value** | **P-adjusted** |
| --- | --- | --- | --- | --- |
| TRGC2 | T cell receptor gamma constant 2 | -0.98 | 2.35E-09 | 1.55E-05 |
| CTSW | cathepsin W | -0.87 | 3.22E-15 | 3.19E-11 |
| HPCAL4 | hippocalcin like 4 | -0.67 | 7.37E-06 | 2.08E-02 |
| TRGV2 | T cell receptor gamma variable 2 | -0.66 | 2.20E-05 | 4.82E-02 |
| ZNF683 | zinc finger protein 683 | -0.65 | 7.92E-08 | 3.92E-04 |
| GOLGA8B | golgin A8 family member B | -0.53 | 1.89E-05 | 4.66E-02 |
| SYNM | synemin | -0.42 | 4.44E-07 | 1.76E-03 |
| PI4KAP1 | phosphatidylinositol 4-kinase alpha pseudogene 1 | 0.52 | 2.44E-05 | 4.82E-02 |

**Table S12**

Differentially expressed genes in CD4+ T cells of *HLA-DRB1**03 positive and negative myositis patients excluding potential outliers. Genes with a positive FC are higher expressed in *HLA-DRB1**03 positive patients and genes with a negative FC are higher expressed in *HLA-DRB1**03 negative patients. P-values were estimated by the Wald test in DESeq2 adjusted for gender, age group, diagnosis, and RIN value. A FDR threshold of 5% based on the method of Benjamini-Hochberg was used to identify significant differentially expressed genes. Abbreviations: FC: Fold Change.

| **Gene symbol** | **Gene name** | **Log2FC** | **P-value** | **P-adjusted** |
| --- | --- | --- | --- | --- |
| TRGC2 | T cell receptor gamma constant 2 | -0.69 | 1.11E-07 | 8.98E-04 |
| PTGDR | prostaglandin D2 receptor | -0.64 | 1.98E-05 | 3.21E-02 |
| CTSW | cathepsin W | -0.55 | 3.95E-11 | 6.40E-07 |
| HPCAL4 | hippocalcin like 4 | -0.55 | 1.14E-05 | 2.31E-02 |
| ZNF683 | zinc finger protein 683 | -0.53 | 3.09E-07 | 1.67E-03 |
| GOLGA8B | golgin A8 family member B | -0.44 | 4.10E-06 | 9.49E-03 |
| NUAK1 | NUAK family kinase 1 | -0.43 | 3.50E-05 | 4.72E-02 |
| SIK1 | salt inducible kinase 1 | 0.29 | 3.04E-05 | 4.47E-02 |
| TCL1A | T cell leukemia/lymphoma 1A | 0.40 | 1.65E-06 | 5.27E-03 |
| PI4KAP1 | phosphatidylinositol 4-kinase alpha pseudogene 1 | 0.40 | 1.12E-06 | 4.54E-03 |
| PHEX | phosphate regulating endopeptidase homolog X-linked | 0.42 | 1.45E-05 | 2.60E-02 |
| AC135983.2 | WAS protein homology region 2 domain containing 1 pseudogene 1 | 0.53 | 1.95E-06 | 5.27E-03 |
